# Supplementary material for: De novo GTP synthesis is a metabolic vulnerability for the interception of brain metastases
Source: Cell Rep Med. 2024 Oct 4;5(10):101755. doi: 10.1016/j.xcrm.2024.101755 (PMC11513854; doi:10.1016/j.xcrm.2024.101755)
Supplement: Document S1. Figures S1–S5, Tables S1 and S2, Methods S1, and Data S1 [file mmc1.pdf]

Cell Reports Medicine, Volume *5*

Supplemental information

De novo GTP synthesis is a metabolic vulnerability for the interception of brain metastases

Agata M. Kieliszek, Daniel Mobilio, Blessing I. Bassey-Archibong, Jarrod W. Johnson, Mathew L. Piotrowski, Elvin D. de Araujo, Abootaleb Sedighi, Nikoo Aghaei, Laura Escudero, Patrick Ang, William D. Gwynne, Cunjie Zhang, Andrew Quaile, Dillon McKenna, Minomi Subapanditha, Tomas Tokar, Muhammad Vaseem Shaikh, Kui Zhai, Shawn C. Chafe, Patrick T. Gunning, J. Rafael Montenegro-Burke, Chitra Venugopal, Jakob Magolan, and Sheila K. Singh

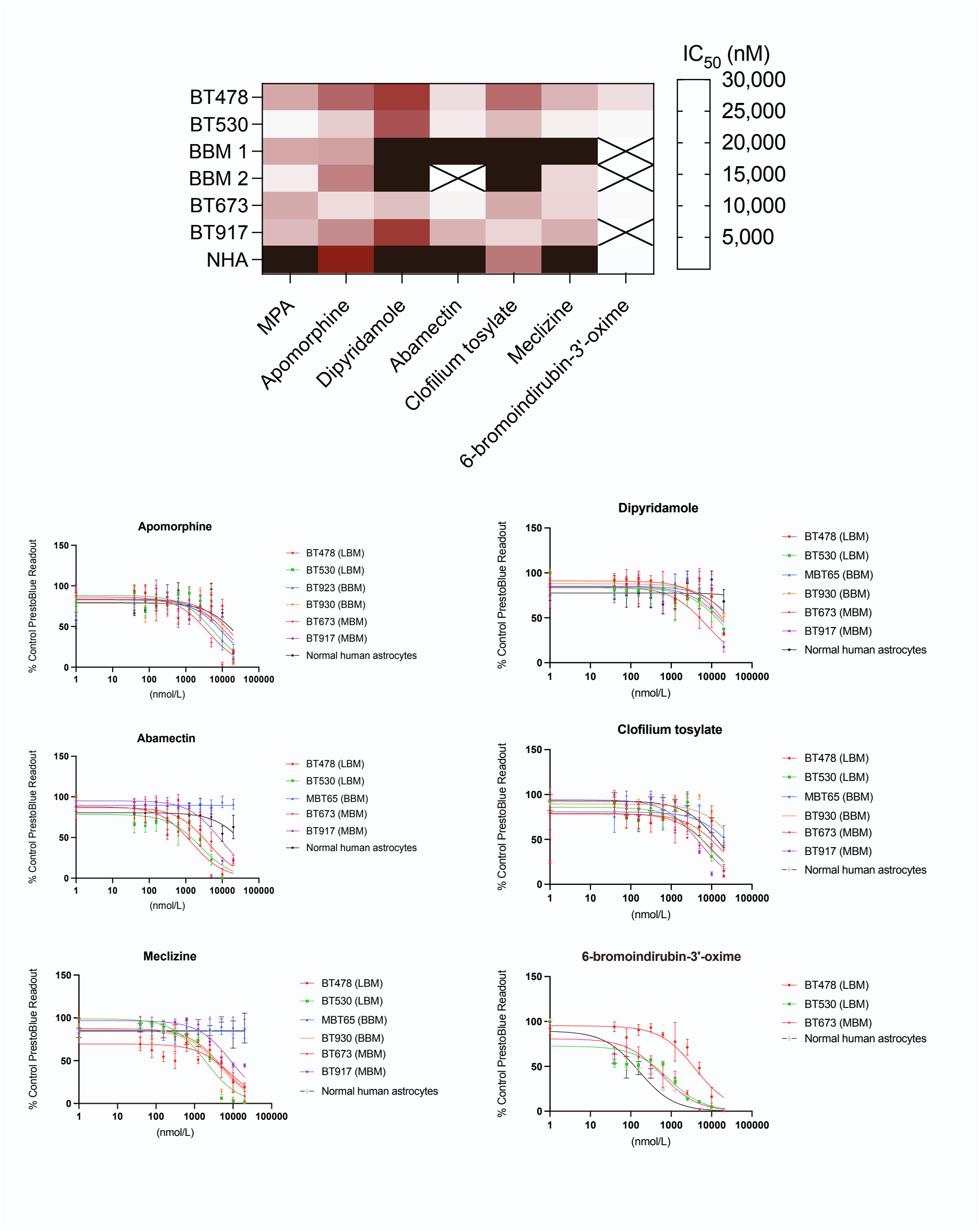

Fig S1. Dose-response curves for effective anti-BMIC compounds, related to Figure 1. Seven compounds shown anti-BMIC activity against a patient-derived lung-BMIC line (BT478) in a preliminary screen of 48 CMap-reveled compounds (*see Fig 1e).* All seven compounds were explored in dose-response assays against multiple BMIC lines and normal human astrocytes. Activity in these assays is summarized in a heat map.

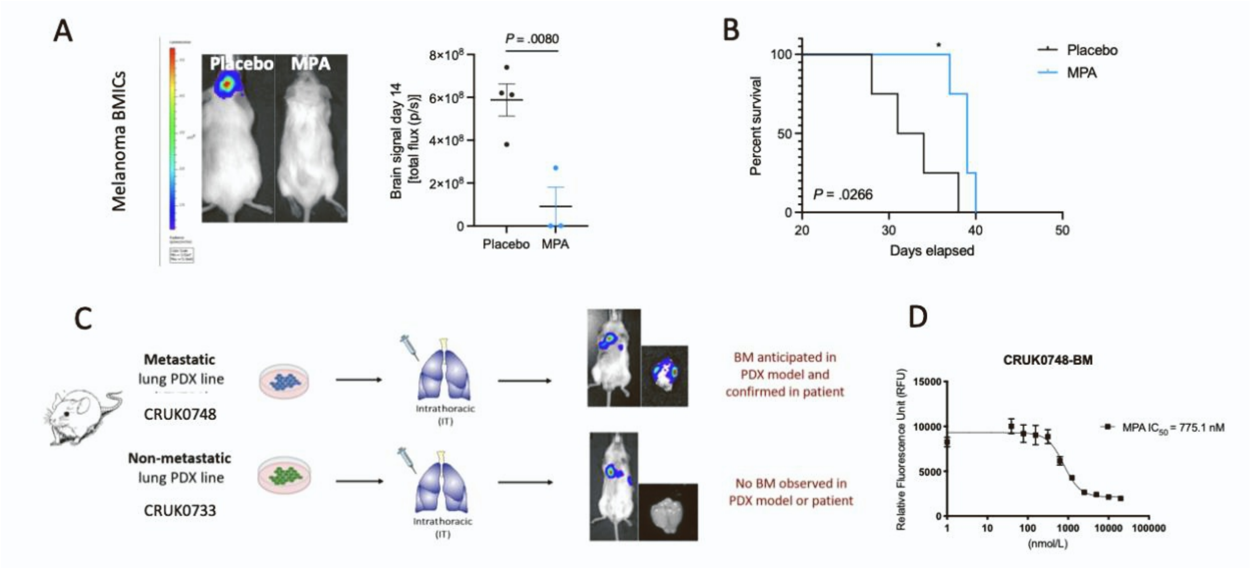

Fig S2. PDX models mimic patient data, related to Figure 3. a, Representative IVIS bioluminescence images of mice 14-days post injection and brain tumor burden comparisons between placebo and MPA groups. IVIS = *In vivo* imaging system. Comparisons were made via a two-tailed unpaired t-test and data are presented as mean ± SED from 4-5 technical replicates. *P* value is indicated. **b** *Kaplan-Meier* survival analysis of placebo and MPA groups following intracranial engraftment of patient-derived BMICs. Comparisons were made via a Log-rank (Mantel-Cox) test, *P* value is indicated**. c**, CRUK0748, a patient- derived lung tumor cell line that metastasized to the brain of the patient it was retrieved from, was confirmed to metastasize to the brains of mice in our animal models following intrathoracic injection. CRUK0733, a patient-derived lung tumor cell line that has not metastasized to the brain of the patient it was retrieved from, did not metastasize in our animal model**. d,** MPA demonstrates a dose-response anti-proliferative effect on BMICs isolated from the brains of mice following CRUK0748 metastasis to the brain**.**

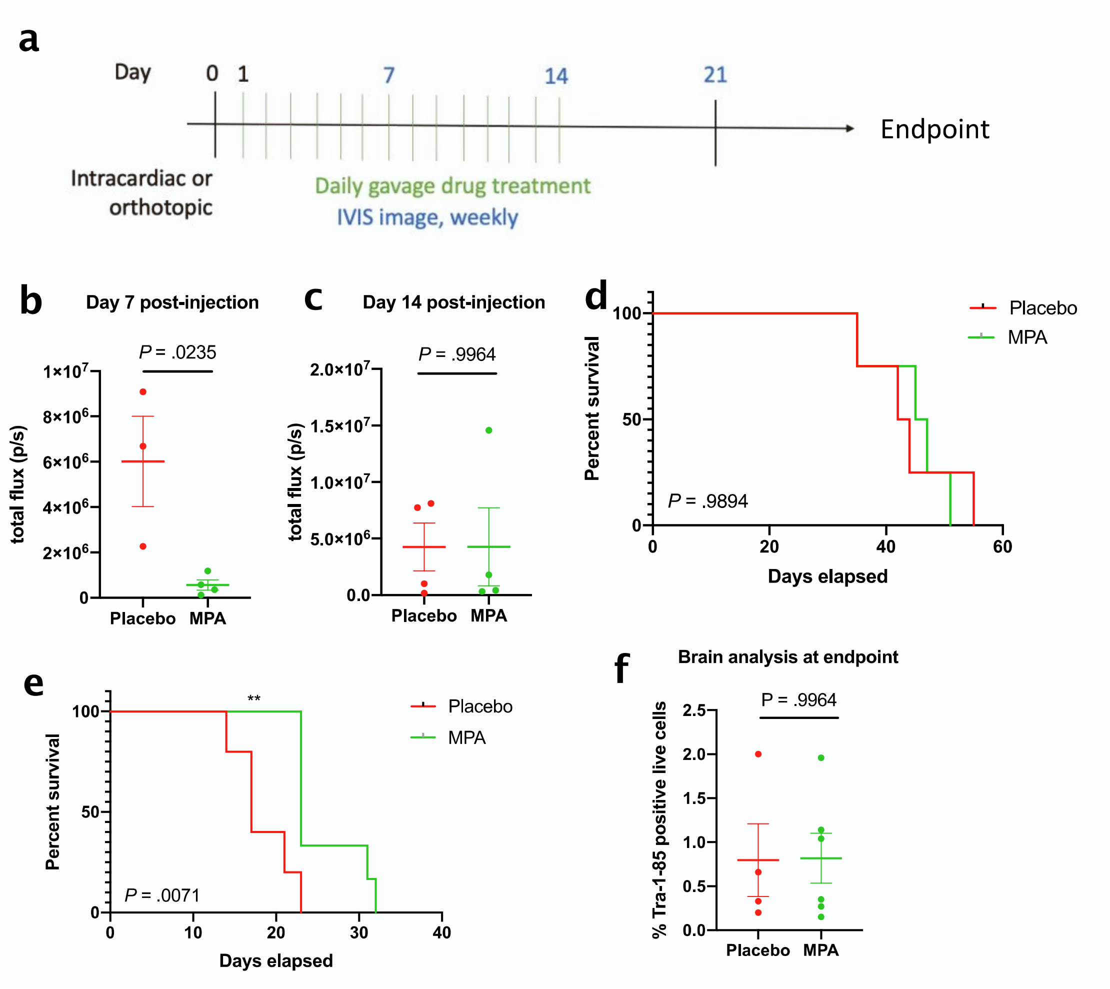

Fig S3. MPA must cross the blood brain barrier to effectively slow the progression of BM over time, related to Figures 3 and 4. a, Mice were either orthotopically (intrathoracically) or intracardiac injected with lung BMICs, followed by daily oral gavage administration of MPA (100 mg/kg) or placebo for 14 days. b, Following intracardiac-injection and subsequent treatment, mice treated with MPA showed a significant decrease in brain tumor burden seven days post-injection by IVIS, (c), no significant reduction in tumor burden following treatment completion (IVIS), and (d) did not experience a survival benefit compared to placebo. e, Following orthotopic injection and subsequent treatment as described In (a), mice treated with MPA showed a significant increase in survival compared to placebo, but (f) had the same number of human cells detected in their brains following humane endpoint by flow cytometry. n.s. = not significant.

Table S1: Bi-directional permeability via MDR1-MDCKII cells models, related to Figure 4. To explore active transport/efflux mechanisms, we employed a MDCK-MDR1 cell monolayer model. Metoprolol is used as a control compound with high permeability and nadolol is used as a control compound with low permeability.

	Mean P_{app} (10^{-6} cm/s) A to B	Mean P_{app} (10^{-6} cm/s) B to A	Efflux Ratio	Rank of P_{app}
**Mycophenolic Acid** (MPA)	26.0	10.0	0.384	High
**Compound 3**	14.8	12.5	0.848	High
**Digoxin** (P-gp substrate)	0.556	11.8	21.2	Low
**Nadolol** (Low permeability)	0.198	Not Detected	-	Low
**Metoprolol** (High Permeability)	29.5	Not Detected	-	High

Table S2: Brain homogenate binding, related Figure 4. Mouse brain homogenate was treated with each compound followed by equilibrium dialysis and mass spectrometry to determine the fraction of bound (65.9%) and un-bound drug (34.1%) for MPA. The mass of Compound **3** could not be detected following completion of the assay, and a proper bound/unbound fraction could not be determined.

Brain homogenate binding

	% Unbound	% Bound
**Mycophenolic Acid** (MPA)	34.1 ± 3.0	65.9 ± 3.8
**Compound 3**	Not Recovered	Not Recovered
**Propranolol** (Control Compound with High Binding)	2.3 ± 0.0	97.6 ± 0.3

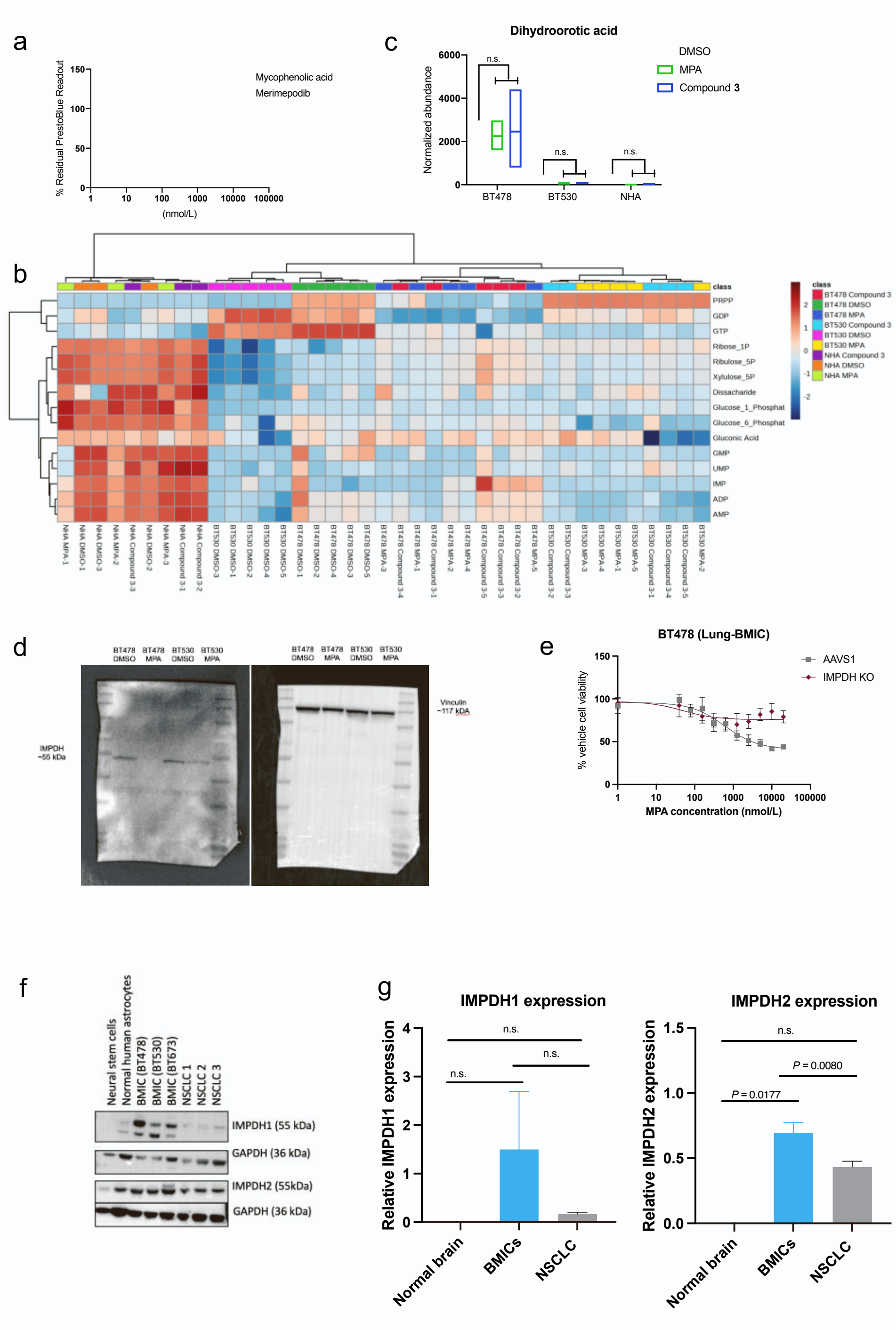

Fig S4. Mechanistic experiments, related to Figure 5. a, Comparative dose-response assay against a patient-derived lung BMIC (BT478) using mycophenolic acid and merimepodib, a selective-IMPDH inhibitor. **b,** LCMS-based metabolomics of the differential polar metabolome of vehicle-, MPA-, or Compound 3 (MLSS2008)-treated BM cells in comparison to normal human astrocytes. Heatmap uses Euclidean distance with Ward's method *via* metaboanalyst. **c**, Box plots depicting relative dihydroorotic acid levels in patient-derived BMIC lines and normal human astrocytes (NHA) with either DMSO-, MPA-, or Compound 3-treatment. N.s. = not significant. **d,** Uncropped blots shown in Figure 5e. **e,** Dose-response assays using AAVS1 and IMPDH KO BT478 cells**. f**, Immunoblot of IMPDH1 and IMPDH2 in normal brain cells (neural stem cells and normal human astrocytes), primary non-small cell lung cancer (NSCLC) cells (CRUK0935, CRUK0883, CRUK0733), and patient-derived BMICs (BT478, BT530, BT673). Cropped from single full blot. **g,** quantification of western blot band intensity is normalized to loading control bands using ImageLab software.

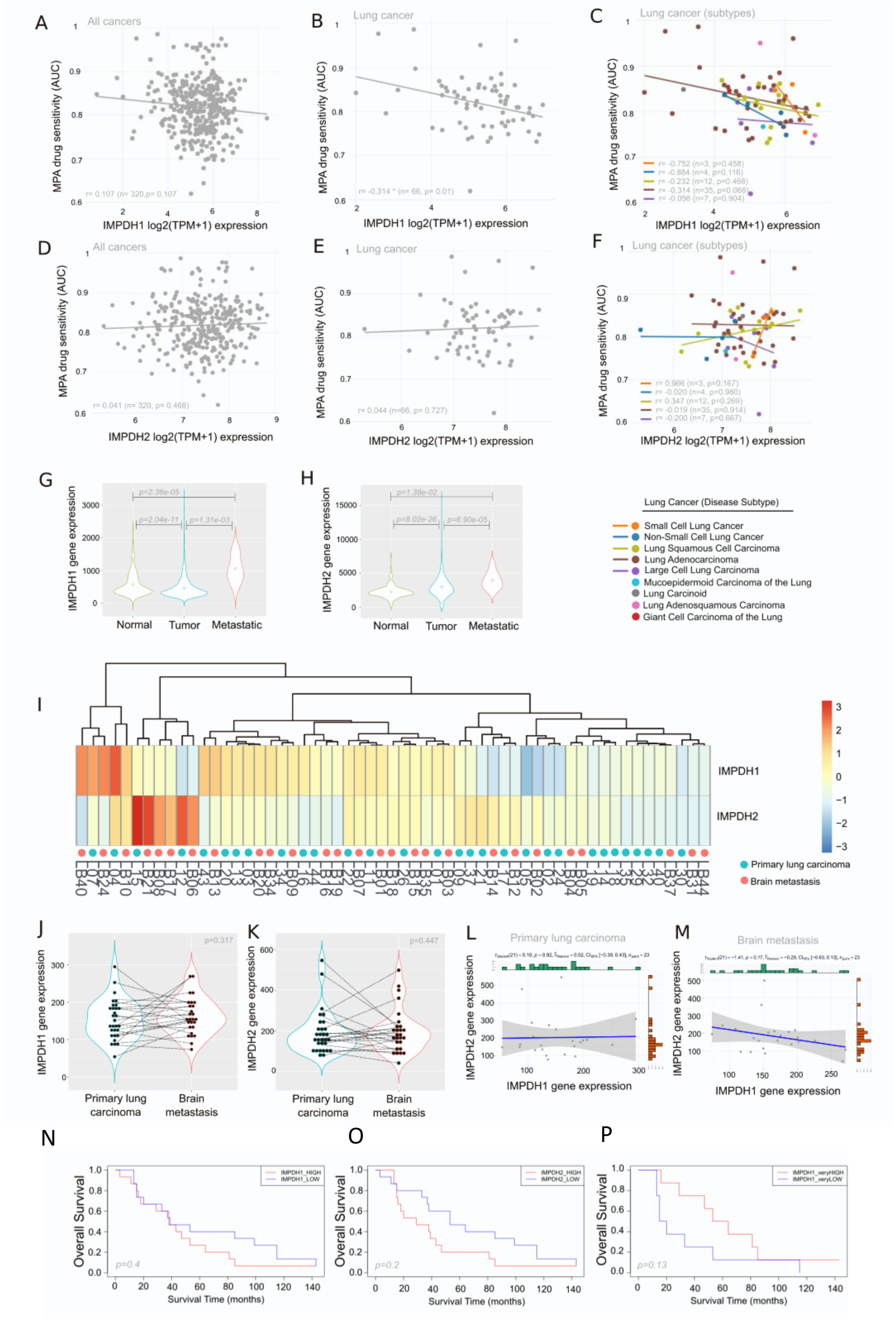

Fig S5. IMPDH as a potential biomarker for lung cancer, related to Figure 5.

(**a-f**) Correlation of mycophenolic acid (MPA) drug sensitivity (area under the dose-response curve (AUC)) based on IMPDH expression across all cancer cell lines (**a**, **d**), lung cancer cell lines (**b**, **e**) and lung cancer cell lines grouped by disease subtype (**c**, **f**) for IMPDH1 (**a-c**) and IMPDH2 (**d-f**) genes. The number of samples (*n*), the Pearson correlation coefficient (r) and p-value (p) are shown. Data was obtained from DepMap portal (Broad Institute) (Drug sensitivity AUC (PRISM repurposing secondary screen); Expression 22Q2 Public, log2(TPM + 1)). (**g-h**) Differential gene expression analysis in normal (n = 391), tumor (n = 1865) and metastatic (n = 8) patient samples (subset for lung tissue) for IMPDH1 (**g**) and IMPDH2 (**h**) gene expression. Krustal Wallis and Dunn test statistical analysis for non-parametric data as shown by TNMplot. Gene chip data were obtained from TNMplot.com. (**i**) Heatmap of IMPDH1 and IMPDH2 gene expression in a cohort of patients with primary lung carcinoma (NSCLC) (n = 30) that developed brain metastasis (n = 27). Scale (Z-score by gene). (**j-k**) Differential expression of paired primary lung carcinoma and brain metastasis patient samples (n = 23) for (**j**) IMPDH1 and (**k**) IMPDH2. Shapiro-Wilk normality test and Paired t-test were performed. (**l-m**) Correlation of IMPDH1 with IMPDH2 gene expression in primary lung carcinoma (n = 30) (**l**) and brain metastasis (n = 27) (**m**) patient samples. The number of samples (*n*), the Pearson correlation coefficient (r) and p-value (p) are shown. (**i-m**) Data was obtained from the processed (Q3 method normalization) GEO dataset (GSE200563). Data acquisition, analysis and visualisation performed using R version 4.1.2 and the following packages: GEOquery, pheatmap, ggplot2 and ggstatsplot. (**n-p**) Kaplan−Meier curves showing the overall survival probability (from primary cancer diagnosis to death) in a cohort of 30 patients with lung carcinoma that developed brain metastasis, for primary tumor IMPDH1 high vs low expression (median cut-off value: 144.72) (**n**), for IMPDH2 high vs low expression (median cut-off value: 162.75) (**o**), and for IMPDH1 very high vs very low expression (1st and 3rd Quartiles cut-off values: <116.19 and >185.31) (**p**). The Cox regression model for survival analysis was used, p-values (p) are shown. Data was obtained from the processed (Q3 method normalization) GEO dataset (GSE200563). Data acquisition, analysis and visualisation performed using R version 4.1.2 and the following packages: GEOquery, survival, and ggplot2.

Methods S1. Chemical Syntheses, related to Figure 4.

(*E*)-6-(4-*tert*-Butyldimethylsilyloxy-6-methoxy-7-methyl-3-oxo-1,3-dihydroisobenzofuran-5-yl)-4-methylhex-4-enoic acid (S1)

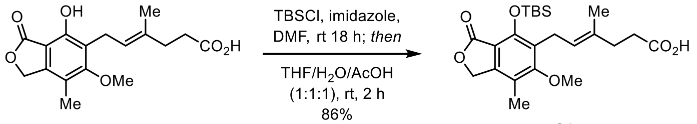

mycophenolic acid (MPA)
[24280-93-1]

S1
[1451181-22-8]

As a variation of the procedure developed by Cholewinski et al.,[1] DMF (20 mL, 0.3 M) was added under an atmosphere of argon to a 100 mL round bottom flask containing mycophenolic acid (2.0 g, 6.24 mmol, 1 equiv). To the solution was added imidazole (2.76 g, 40.6 mmol, 6.5 equiv) followed by TBSCl (3.76 g, 24.96 mmol, 4.0 equiv) and the reaction mixture was stirred at room temperature for 18 hours. The reaction mixture was diluted with water (42 mL) and EtOAc (30 mL). The layers were separated and the aqueous was extracted with ethyl acetate (30 mL). The combined organics were washed once with 1% HCl and three times with water, dried with Na_2SO_4, and concentrated under reduced pressure to obtain a colourless oil. The oil was then dissolved in a 1:1:1 solvent mixture of THF:H_2O:AcOH (30 mL) and stirred at room temperature for two hours. The reaction was diluted with water and extracted with ethyl acetate. The combined organics were washed with water twice, dried with Na_2SO_4, and concentrated under reduced pressure to obtain a colourless oil which solidified under vacuum. The white solids were vacuum filtered and washed excessively with hexanes to obtain TBS-protected mycophenolic acid **S1** (2.34 g, 5.38 mmol, 86%) as a white solid without the need for further purification. R_f = 0.66 (60:39:1 EtOAc/Hex/AcOH). Mp 129–131 °C (lit.[Error! Bookmark not defined.] 94–96 °C). ^{1}H NMR (400 MHz, $CDCl_3$): d 5.20 (tq, J = 6.4, 1.3 Hz, 1H), 5.06 (s, 2H), 3.73 (s, 3H), 3.38 (d, J = 6.4 Hz, 2H), 2.44−2.36 (m, 2H), 2.33−2.24 (m, 2H), 2.15 (s, 3H), 1.75 (d, J = 1.3 Hz, 3H), 1.02 (s, 9H), 0.24 (s, 6H). ^{13}C NMR (100 MHz, $CDCl_3$) δ 179.1, 169.4, 163.3, 151.9, 146.2, 133.5, 127.7,

124.0, 118.1, 111.8, 67.8, 60.9, 34.2, 32.8, 26.2 (3C), 23.8, 18.9, 16.5, 11.6, −3.4 (2C). LCMS (ESI) *m/z*: 457.2017 calcd for $C_{23}H_{34}O_6SiNa^+$ [M + Na]$^+$; Found 457.2038. Spectral data were consistent with those previously reported.[Error! Bookmark not defined.]

2-(4-Boc-piperazino)ethyl (*E*)-6-(4-*tert*-Butyldimethylsilyloxy-6-methoxy-7-methyl-3-oxo-1,3-dihydroisobenzofuran-5-yl)-4-methylhex-4-enoate (S2)

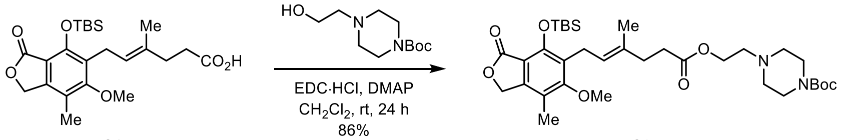

S1 **S2**

To a 10 mL round bottom flask containing TBS-protected mycophenolic acid **S1** (0.200 g, 0.46 mmol, 1 equiv) under an atmosphere of argon was added CH_2Cl_2 (2 mL, 0.23 M). To the solution was added 4-(2-hydroxyethyl)-1-Boc-piperazine (0.12 g, 0.55 mmol, 1.2 equiv), DMAP (0.005 g, 0.046 mmol, 0.1 equiv), and EDC·HCl (0.10 g, 0.55 mmol, 1.2 equiv) and the reaction was stirred for 17 hours. The reaction mixture was concentrated under reduced pressure and diluted with water ethyl acetate. The layers were separated and the aqueous was extracted with ethyl acetate. The combined organics were washed twice with water and brine, dried with Na_2SO_4, and concentrated under reduced pressure. The product was purified by column chromatography (85% EtOAc/Hexanes) providing the ester **S2** as a colourless oil (0.26 g, 0.4 mmol, 86%). R_f = 0.25 (70% EtOAc/Hex). ^1H NMR (400 MHz, $CDCl_3$): d 5.18 (tq, *J* = 6.4, 1.4 Hz, 1H), 5.07 (s, 2H), 4.15 (t, *J* = 5.9 Hz, 2H), 3.74 (s, 3H), 3.42−3.37 (m, 6H), 2.58 (t, *J* = 5.9 Hz, 2H), 2.45−2.34 (m, 6H), 2.31−2.24 (m, 2H), 2.15 (s, 3H), 1.75 (d, *J* = 1.4 Hz, 3H), 1.45 (s, 9H), 1.03 (s, 9H), 0.24 (s, 6H). ^{13}C NMR (100 MHz, $CDCl_3$): d 173.2, 169.2, 163.2, 154.7, 151.7, 146.1, 133.7, 127.6, 123.6, 118.0, 111.6, 79.6, 67.6, 61.6, 60.7, 56.7, 53.1 (2C), 44.1 (br), 43.0 (br), 34.4, 33.0, 28.4 (3C), 26.1 (3C), 18.7, 16.4, 11.4, −3.5 (2C). LCMS (ESI) *m/z*: 647.3722 calcd for $C_{34}H_{55}N_2O_8Si^+$ [M + H]$^+$; Found 647.3696.

Mycophenolic acid, 2-(4-Boc-piperazino)ethyl ester (2)

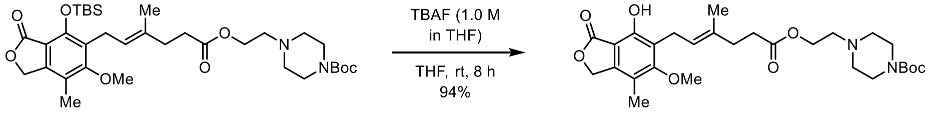

S2 **2**

To a 5 mL round bottom flask containing **S2** (0.075 g, 0.12 mmol, 1 equiv) under an atmosphere of argon was added THF (0.6 mL, 0.23 M). TBAF as a 1.0 M solution in THF (0.14 mL, 0.14 mmol, 1.2 equiv) was added and the reaction stirred at room temperature for eight hours. The reaction was diluted with water and extracted with ethyl acetate. The combined organics were dried with Na_2SO_4 and concentrated under reduced pressure. The product was purified by column chromatography eluting with 85% EtOAc/Hexanes providing the free phenol **2** as a colourless oil (0.058 g, 0.1 mmol, 94%). R_f = 0.25 (70% EtOAc/Hex). ^1H NMR (400 MHz, $CDCl_3$): d 7.96 (brs, 1H), 5.0 (dq, *J* = 6.9, 1.4 Hz, 1H), 5.17 (s, 2H), 4.12 (t, *J* = 5.8 Hz, 2H), 3.75 (s, 3H), 3.44−3.38 (m, 4H), 3.36 (d, *J* = 6.9 Hz, 2H), 2.58 (t, *J* = 5.8 Hz, 2H), 2.47−2.37 (m, 6H), 2.31−2.24 (m, 2H), 2.13 (s, 3H), 1.78 (d, *J* = 1.4 Hz, 3H), 1.44 (s, 9H). ^{13}C NMR (100 MHz, $CDCl_3$): d 173.3, 172.9, 163.7, 154.8, 153.8, 144.2, 134.1, 123.0, 122.3, 116.7, 106.5, 79.7, 70.1, 62.0, 61.1, 56.8, 53.4 (2C), 44.2 (br), 43.0 (br), 34.8, 33.1, 28.5 (3C), 22.8, 16.2, 11.7. LCMS (ESI) *m/z*: 533.2857 calcd for $C_{28}H_{41}N_2O_8^+$ [M + H]$^+$; Found 533.2870.

2-(1-Pyrrolidinyl)ethyl (*E*)-6-(4-*tert*-Butyldimethylsilyloxy-6-methoxy-7-methyl-3-oxo-1,3-dihydroisobenzofuran-5-yl)-4-methylhex-4-enoate (S3)

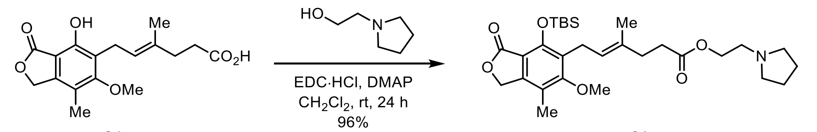

To a 100 mL round bottom flask containing TBS-protected mycophenolic acid **S1** (2.29 g, 5.28 mmol, 1 equiv) under an atmosphere of argon was added CH_2Cl_2 (23 mL, 0.23 M). To the solution, was added *N*-(2-hydroxyethyl)pyrrolidine (0.74 mL, 6.34 mmol, 1.2 equiv), DMAP (0.064 g, 0.53 mmol, 0.1 equiv), and EDC·HCl (1.21 g, 6.34 mmol, 1.2 equiv) and the reaction was stirred for 24 hours. The reaction mixture was concentrated under reduced pressure and diluted with 70 mL water and 70 mL ethyl acetate. The layers were separated and the aqueous was extracted with ethyl acetate. The combined organics were washed with water (2 × 40 mL), brine (2 × 40 mL), dried with Na_2SO_4, and concentrated under reduced pressure providing ester **S3** as a colourless oil (2.70 g, 5.07 mmol, 96%) that was used without any further purification. R_f = 0.23 (4% MeOH/CH_2Cl_2). ^{1}H NMR (400 MHz, $CDCl_3$): d 5.16 (tq, *J* = 5.1, 1.4 Hz, 1H), 5.04 (s, 2H), 4.15 (t, *J* = 5.9 Hz, 2H), 3.71 (s, 3H), 3.35 (d, *J* = 6.3 Hz, 2H), 2.71 (t, *J* = 5.9 Hz, 2H), 2.60−2.57 (brs, 4H), 2.40−2.36 (m, 2H), 2.28−2.24 (m, 2H), 2.13 (s, 3H), 1.82−1.76 (m, 4H), 1.73 (brs, 3H), 1.00 (s, 9H), 0.21 (s, 6H). ^{13}C NMR (100 MHz, $CDCl_3$): d 173.3, 169.2, 163.2, 151.7, 146.1, 133.7, 127.7, 123.6, 118.0, 111.7, 67.7, 62.9, 60.8, 54.6, 54.5, 34.4, 33.0, 26.1, 23.7, 23.5, 18.8, 16.4, 11.5, −3.4 (2C). LCMS (ESI) *m/z*: 532.3089 calcd for $C_{29}H_{46}N_2O_6Si^+$ [M + H]$^+$; Found 532.3111.

Mycophenolic acid, 2-(1-pyrrolidinyl)ethyl ester (3)

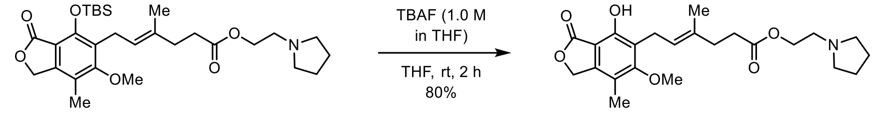

To a 100 mL round bottom flask containing **S3** (2.66 g, 5.0 mmol, 1 equiv) under an atmosphere of argon was added THF (22 mL, 0.23 M). TBAF as a 1.0 M solution in THF (6 mL, 6 mmol, 1.2 equiv) was added and the reaction stirred at room temperature for two hours. The reaction was diluted with water and extracted with ethyl acetate. The combined organics were dried with Na_2SO_4 and concentrated under reduced pressure. The product was purified by column chromatography eluting with 6% MeOH/DCM providing the free phenol **3** as a white solid (1.67 g, 4.0 mmol, 80%). R_f = 0.32 (6% MeOH/CH_2Cl_2). Mp 82–83 °C. ^{1}H NMR (400 MHz, $CDCl_3$): d 7.7−6.7 (brs, 1H), 5.21 (tq, *J* = 6.9, 1.3 Hz, 1H), 5.17 (s, 2H), 4.12 (t, *J* = 5.9 Hz, 2H), 3.75 (s, 3H), 3.37 (d, *J* = 6.9 Hz, 2H), 2.71 (t, *J* = 5.9 Hz, 2H), 2.63−2.54 (m, 4H), 2.44−2.38 (m, 2H), 2.33−2.26 (m, 2H), 2.13 (s, 3H), 1.82−1.75 (m, 7H). ^{13}C NMR (100 MHz, $CDCl_3$): d 173.5, 172.9, 163.7, 154.2, 144.2, 134.0, 123.2, 122.6, 116.4, 106.6, 70.0, 63.6, 61.1, 54.9 (2C), 54.6, 34.8, 33.2, 23.6 (2C), 22.8, 16.2, 11.6. LCMS (ESI) *m/z*: 418.2224 calcd for $C_{23}H_{32}NO_6^+$ [M + H]$^+$; Found 418.2234.

Mycophenolic acid, 4-methylpiperazine amide (4)

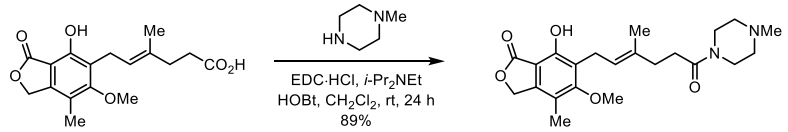

mycophenolic acid (MPA) **4**

Diisopropylethylamine (165 mL, 0.95 mmol) was added to a suspension of mycophenolic acid (100 mg, 0.31 mmol), EDC·HCl (90 mg, 0.47 mmol), HOBt (63 mg, 0.47 mmol), and *N*-methylpiperidine (38 mg, 0.38 mmol) in CH_2Cl_2 (6 mL). After stirring the reaction mixture overnight at rt for 24 h, it was diluted with CH_2Cl_2 (20 mL), washed with saturated Na_2CO_3 (20 mL), dried over Na_2SO_4, and concentrated under reduced pressure. Flash chromatography (0→15% MeOH/CH_2Cl_2) provided amide **4** as a pale yellow oil (112 mg, 0.28 mmol, 89%). ^{1}H NMR (400 MHz, $CDCl_3$): d 5.22 (dd, *J* = 6.8, 1.3 Hz, 2H), 5.20 (s, 2H), 3.76 (s, 3H), 3.63−3.56 (m, 2H), 3.48−3.42 (m, 2H), 3.39 (d, *J* = 6.8 Hz, 2H), 2.43−2.26 (m, 8H), 2.29 (s, 3H), 2.15 (s, 3H), 1.82 (d, *J* = 1.3 Hz, 3H). ^{13}C NMR (100 MHz, $CDCl_3$): d 173.1, 171.3, 163.8, 153.8, 144.1, 135.0, 122.5, 122.4, 116.9, 106.6, 70.2, 61.2, 55.3, 54.9, 46.2, 45.6, 41.6, 35.2, 32.1, 22.8, 16.6, 11.7.

Mycophenolic acid, 3-(azetidin-1-yl)propyl amide (5)

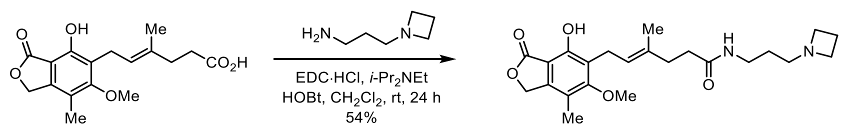

mycophenolic acid (MPA) **5**

Diisopropylethylamine (165 mL, 0.95 mmol) was added to a suspension of mycophenolic acid (100 mg, 0.31 mmol), EDC·HCl (90 mg, 0.47 mmol), HOBt (63 mg, 0.47 mmol), and 3-(azetidin-1-yl)propylamine (42 mg, 0.37 mmol) in CH_2Cl_2 (6 mL). After stirring the reaction mixture overnight at rt for 24 h, it was diluted with CH_2Cl_2 (20 mL), washed with saturated Na_2CO_3 (20 mL), dried over Na_2SO_4, and concentrated under reduced pressure. Flash chromatography (0→10% MeOH/CH_2Cl_2) provided amide **5** as a pale yellow oil (70 mg, 0.17 mmol, 54%). ^{1}H NMR (400 MHz, $CDCl_3$): d 7.20 (t, *J* = 6.1 Hz, 1H), 5.21 (tq, *J* = 6.9, 1.5 Hz, 1H), 5.19 (s, 2H), 4.00 (appt, *J* = 7.3 Hz, 4H), 3.75 (s, 3H) 3.37 (d, *J* = 6.9 Hz, 2H), 3.28 (appq, *J* = 6.1 Hz, 2H), 3.06 (appt, *J* = 6.7 Hz, 2H), 2.52 (app pent, *J* = 8.0 Hz, 2H), 2.34−2.24 (m, 4H), 2.14 (s, 3H), 1.84 (pent, *J* = 6.5 Hz, 2H), 1.80 (s, 3H). ^{13}C NMR (100 MHz, $CDCl_3$): d 173.8, 173.0, 163.8, 153.9, 144.3, 134.8, 122.9, 122.4, 116.8, 106.6, 70.1, 61.2, 53.9 (2C), 52.8, 35.9, 35.3, 35.0, 24.2, 22.8, 16.4, 16.2, 11.7.

2-(1-Pyrrolidinyl)ethyl (*E*)-6-(4-*tert*-butyldimethylsilyloxy-6-methoxy-7-methyl-3-oxo-1,3-dihydroisobenzofuran-5-yl)-4-methylhex-4-enoate (S4)

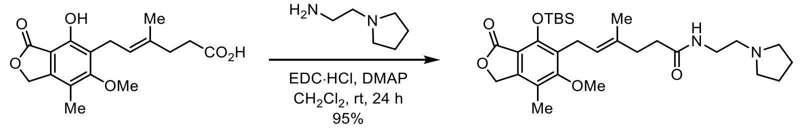

S1 **S4**

To a 10 mL round bottom flask containing TBS-protected mycophenolic acid **S1** (0.20 g, 0.46 mmol, 1 equiv) under an atmosphere of argon was added CH_2Cl_2 (2 mL, 0.23 M). To the solution was added 2-(pyrrolidin-1-yl)ethylamine (0.063 g, 0.55 mmol, 1.2 equiv), DMAP (0.005 g, 0.046 mmol, 0.1 equiv), and EDC·HCl (0.10 g,

0.55 mmol, 1.2 equiv) and the reaction was stirred for 17 hours. The reaction mixture was concentrated under reduced pressure and diluted with water ethyl acetate. The layers were separated and the aqueous was extracted with ethyl acetate. The combined organics were washed twice with water and brine, dried with Na_2SO_4, and concentrated under reduced pressure. The crude was purified by column chromatography (15% $MeOH/CH_2Cl_2$) providing amide **S4** a yellow oil (0.23 g, 0.43 mmol, 95%). R_f = 0.38 (20% $MeOH/CH_2Cl_2$). ^1H NMR (400 MHz, $CDCl_3$): d 7.04 (brs, 1H), 5.17 (brt, J = 6.4 Hz, 1H), 5.06 (s, 2H), 3.73 (s, 3H), 3.46 (appq, J = 5.5 Hz, 2H), 3.36 (d, J = 6.4 Hz, 2H), 3.03−2.92 (m, 4H), 2.89 (appt, J = 5.6 Hz, 2H), 2.35−2.26 (brs, 4H), 2.14 (s, 3H), 2.02−1.92 (m, 4H), 1.75 (d, J = 1.4 Hz, 3H), 1.01 (s, 9H), 0.22 (s, 6H). ^{13}C NMR (100 MHz, $CDCl_3$): d 173.4, 169.4, 163.4, 151.8, 146.2, 134.3, 127.8, 123.5, 118.1, 111.7, 67.8, 60.9, 55.5, 54.3 (2C), 36.6, 35.2 (2C), 26.2 (3C), 23.8, 23.5 (2C), 18.9, 16.5, 11.6, −3.4 (2C). LCMS (ESI) *m/z*: 531.3249 calcd for $C_{29}H_{47}N_2O_5Si^+$ [M + H]$^+$; Found 531.3244.

Mycophenolic acid, 2-(1-pyrrolidinyl)ethyl amide (6)

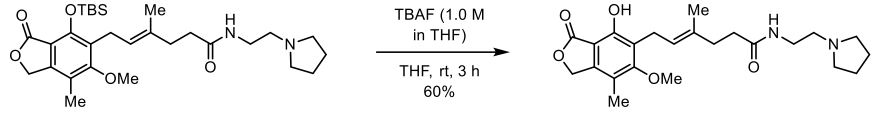

To a 5 mL round bottom flask containing TBS-protected phenol **S4** (0.10 g, 0.19 mmol, 1 equiv) under an atmosphere of argon was added THF (0.94 mL, 0.23 M). TBAF as a 1.0 M solution in THF (0.23 mL, 0.23 mmol, 1.2 equiv) was added and the reaction stirred at room temperature for three hours. The reaction was diluted with water and extracted with ethyl acetate. The combined organics were dried with Na_2SO_4 and concentrated under reduced pressure. The product was purified by column chromatography eluting with 15% $MeOH/CH_2Cl_2$ providing the free phenol **6** as a white solid (0.047 g, 0.11 mmol, 60%). R_f = 0.38 (6% $MeOH/CH_2Cl_2$). ^1H NMR (400 MHz, $CDCl_3$): d 7.60 (brs, 1H), 5.23 (tq, J = 6.9, 1.4 Hz, 1H), 5.19 (s, 2H), 3.75 (s, 3H), 3.58 (appq, J = 5.5 Hz, 2H), 3.37 (d, J = 6.9 Hz, 2H), 3.33−3.10 (brs, 4H), 3.12 (appt, J = 5.5 Hz, 2H), 2.40−2.27 (m, 4H), 2.14 (s, 3H), 2.13−2.05 (m, 4H), 1.81 (d, J = 1.4 Hz, 3H). ^{13}C NMR (175 MHz, $CDCl_3$): d 173.8, 173.0, 163.8, 154.2, 144.3, 134.7, 123.1, 122.5, 116.6, 106.6, 70.1, 61.2, 55.9, 54.5 (2C), 36.0, 35.3, 35.0, 23.4 (2C), 22.8, 16.3, 11.7. LCMS (ESI) *m/z*: 417.2384 calcd for $C_{23}H_{33}N_2O_5^+$ [M + H]$^+$; Found 417.2391.

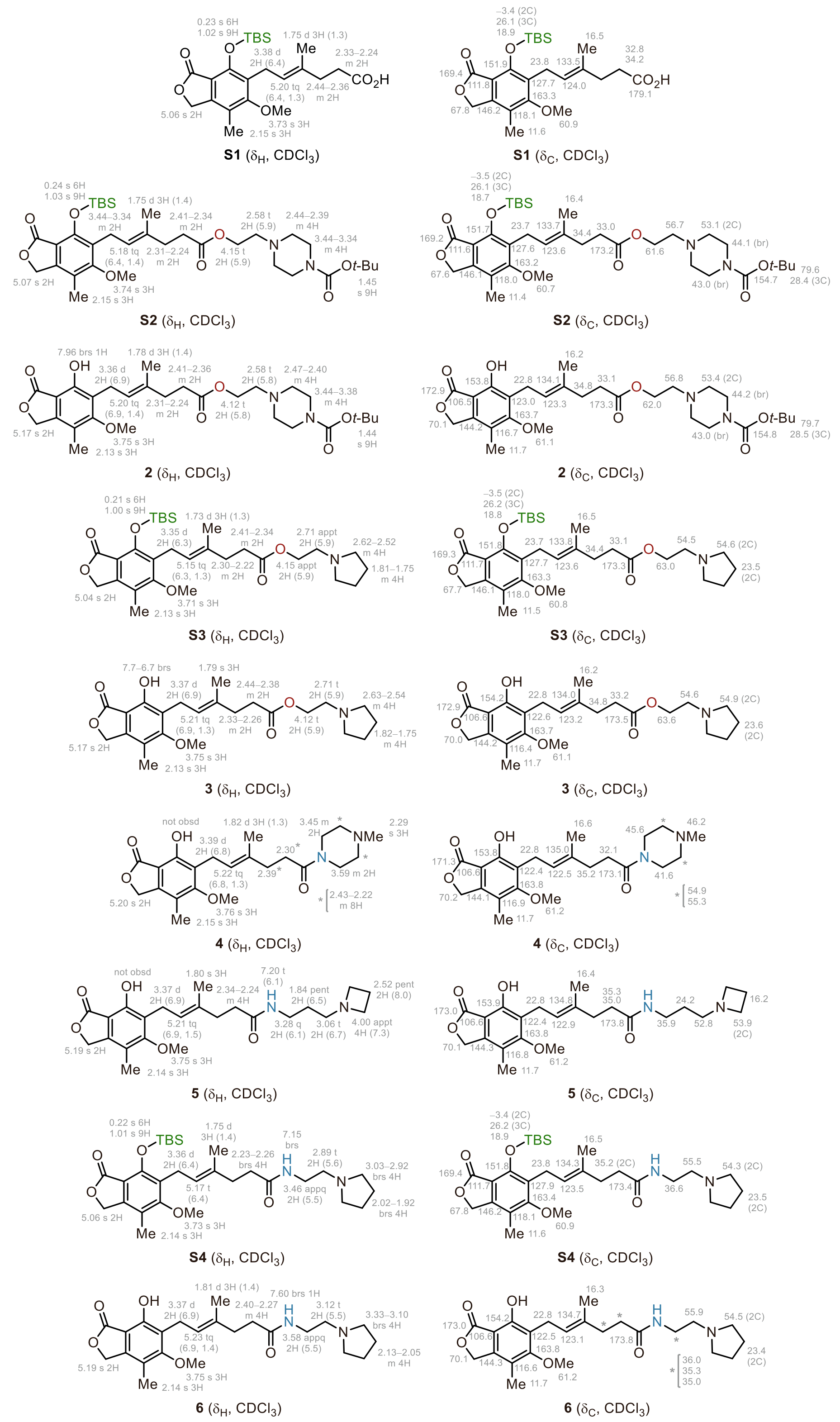

Figure S6. NMR assignments for mycophenolic esters and amides, related to Figure 4.

Data S1. ^{1}H and ^{13}C NMR Spectra, related to Figure 4.

(*E*)-6-(4-TBSO-6-methoxy-7-methyl-3-oxo-1,3-dihydroisobenzofuran-5-yl)-4-methylhex-4-enoic acid (**S1**) (^{1}H NMR; 400 MHz; $CDCl_3$)

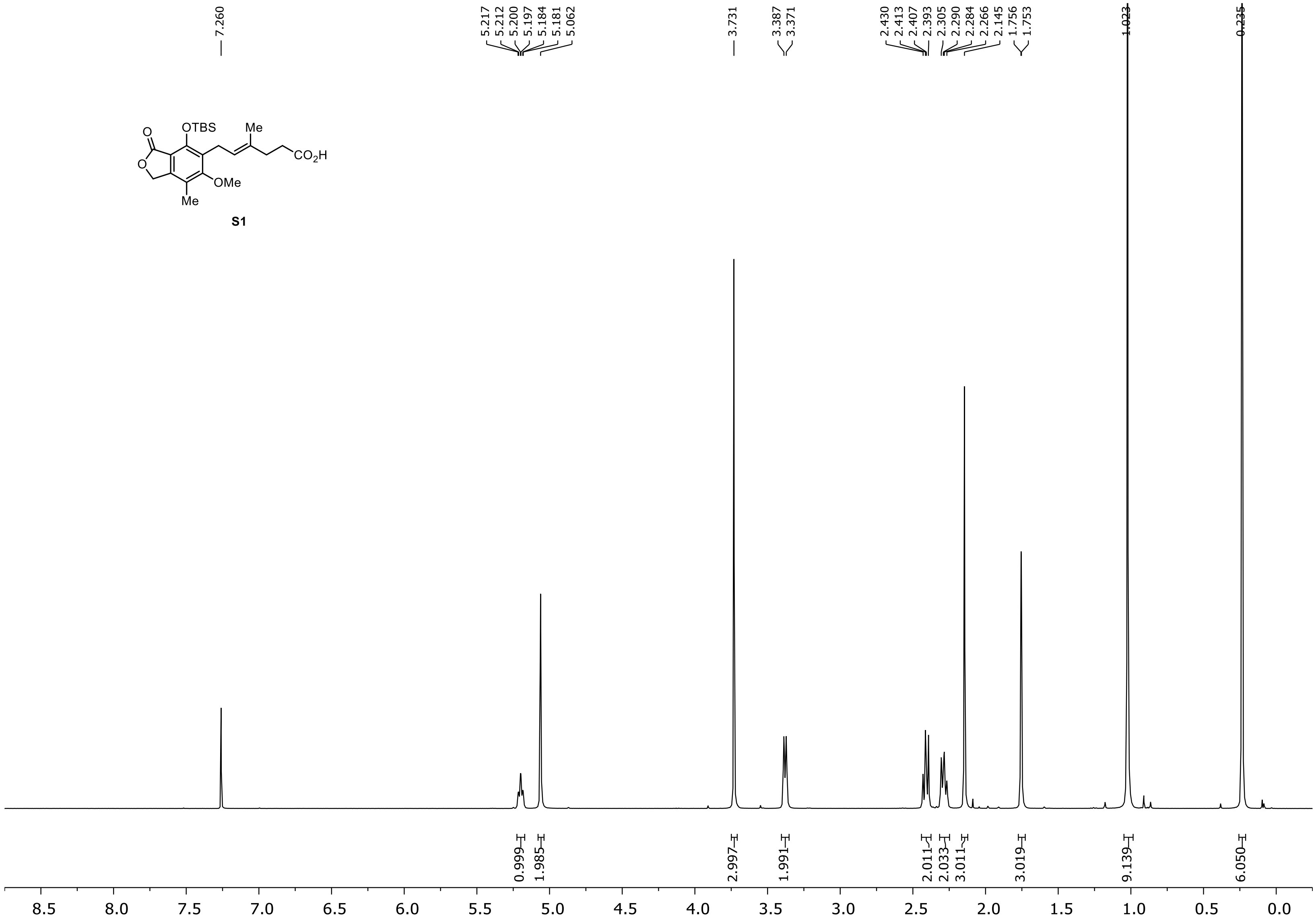

(*E*)-6-(4-*tert*-Butyldimethylsilyloxy-6-methoxy-7-methyl-3-oxo-1,3-dihydroisobenzofuran-5-yl)-4-methylhex-4-enoic acid (**S1**) (^{13}C NMR; 100 MHz; $CDCl_3$)

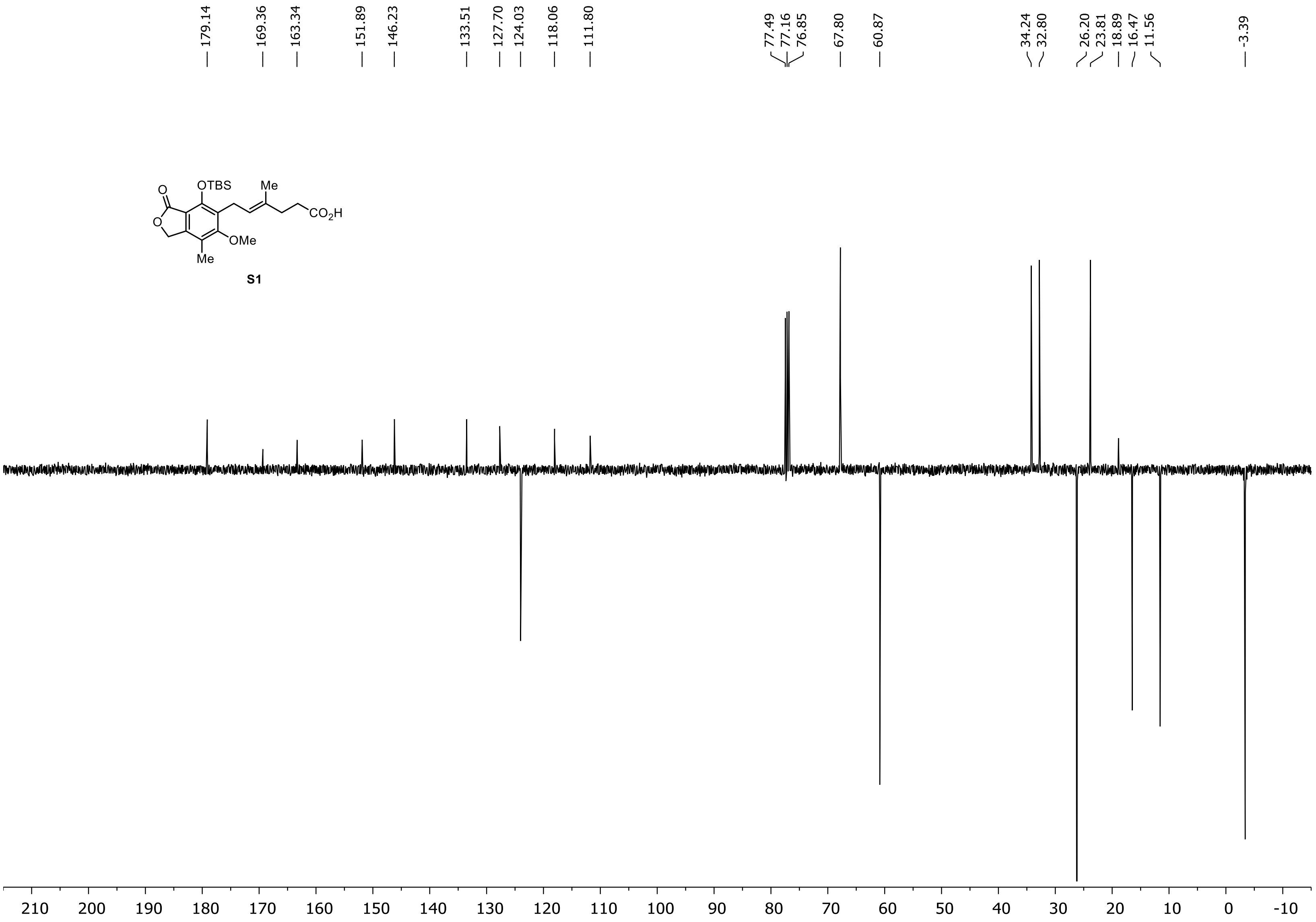

2-(4-Boc-piperazino)ethyl (*E*)-6-(4-*tert*-Butyldimethylsilyloxy-6-methoxy-7-methyl-3-oxo-1,3-dihydroisobenzofuran-5-yl)-4-methylhex-4-enoate (**S2**) (^{1}H NMR; 400 MHz; $CDCl_3$)

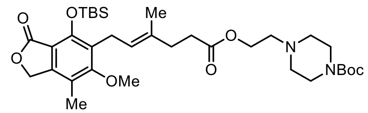

S2

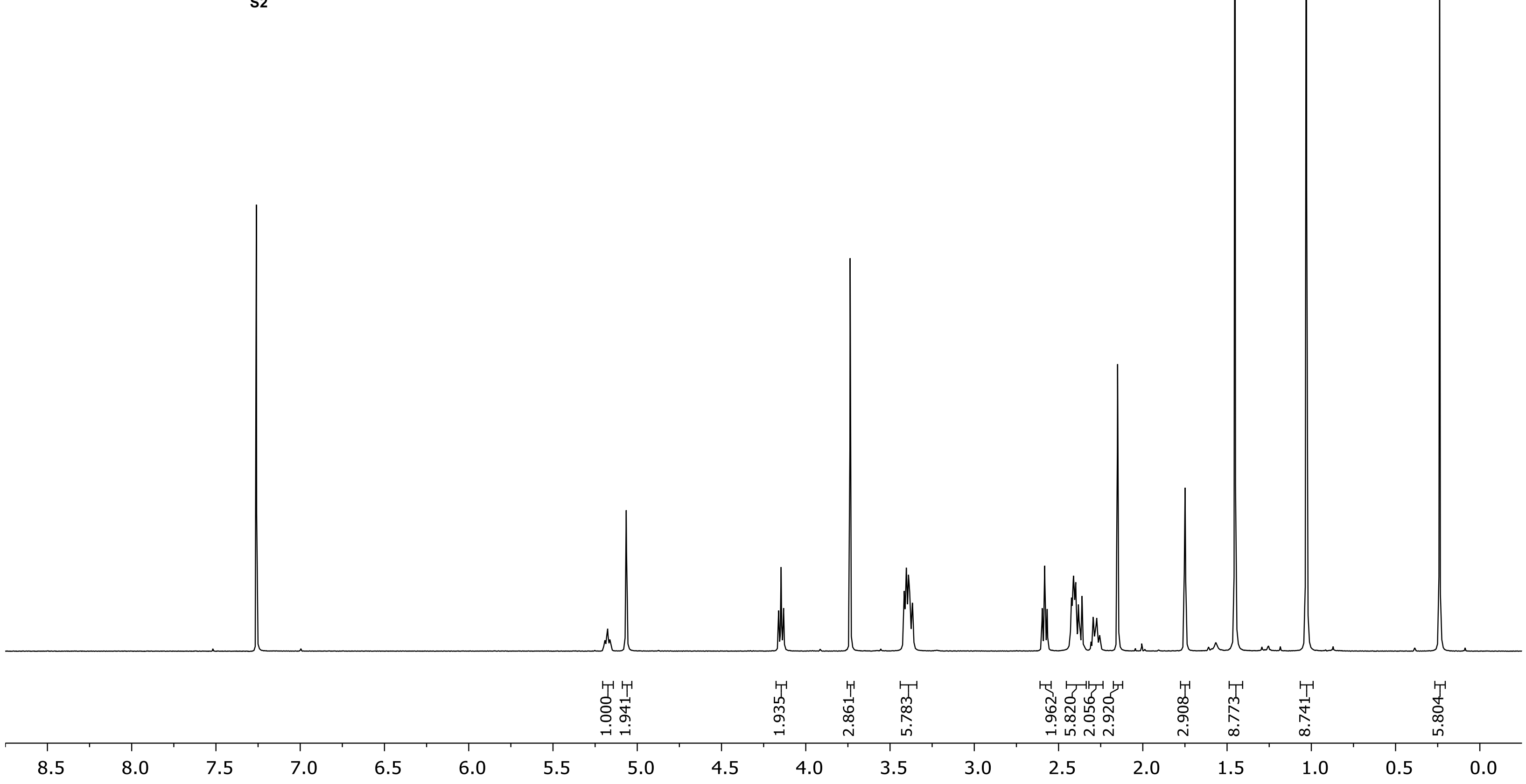

2-(4-Boc-piperazino)ethyl (*E*)-6-(4-*tert*-Butyldimethylsilyloxy-6-methoxy-7-methyl-3-oxo-1,3-dihydroisobenzofuran-5-yl)-4-methylhex-4-enoate (**S2**) (^{13}C NMR; 100 MHz; $CDCl_3$)

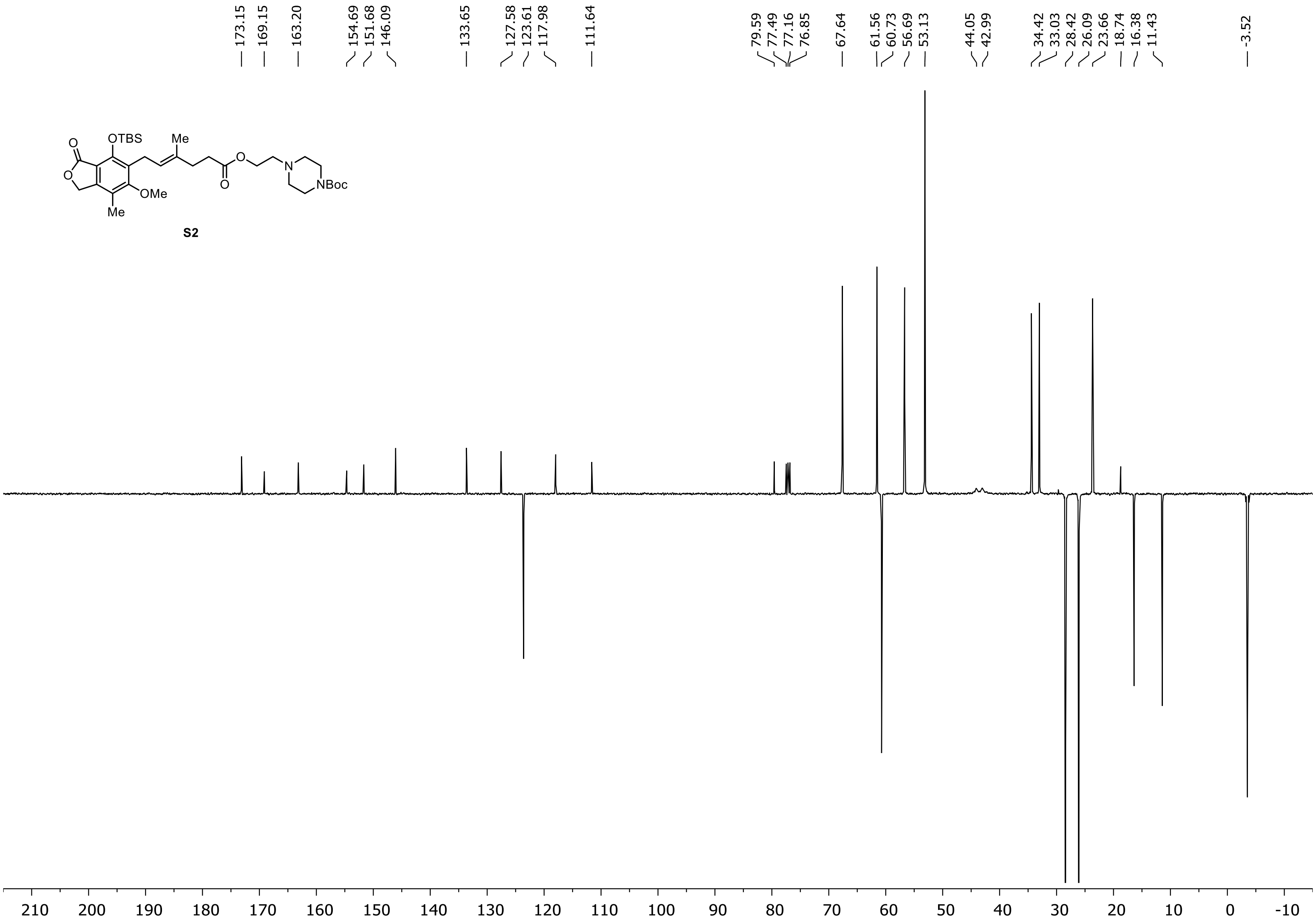

Mycophenolic acid, 2-(4-Boc-piperazino)ethyl ester (**2**) (^{1}H NMR; 400 MHz; $CDCl_3$)

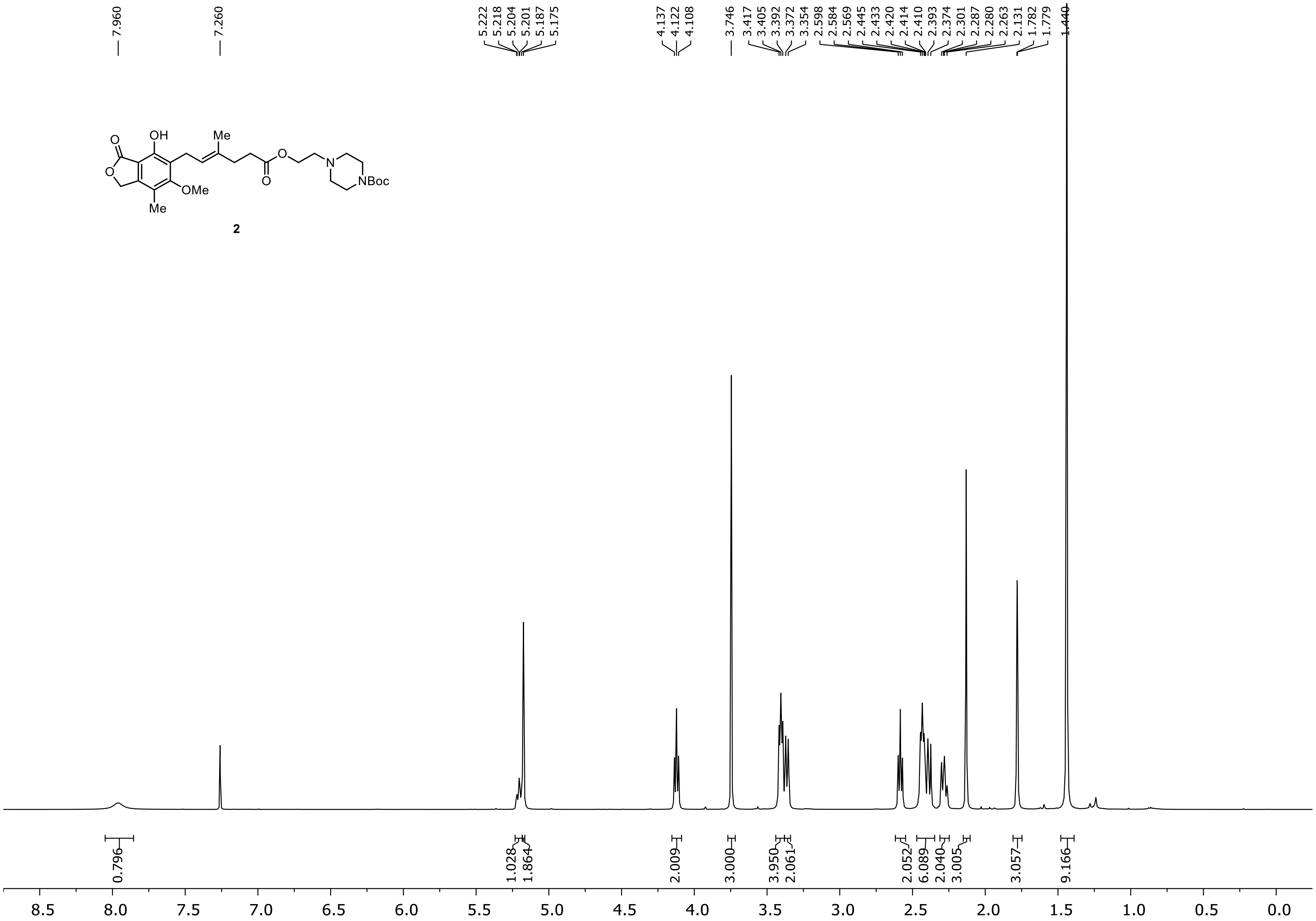

Mycophenolic acid, 2-(4-Boc-piperazino)ethyl ester (**2**) (^{13}C NMR; 100 MHz; $CDCl_3$)

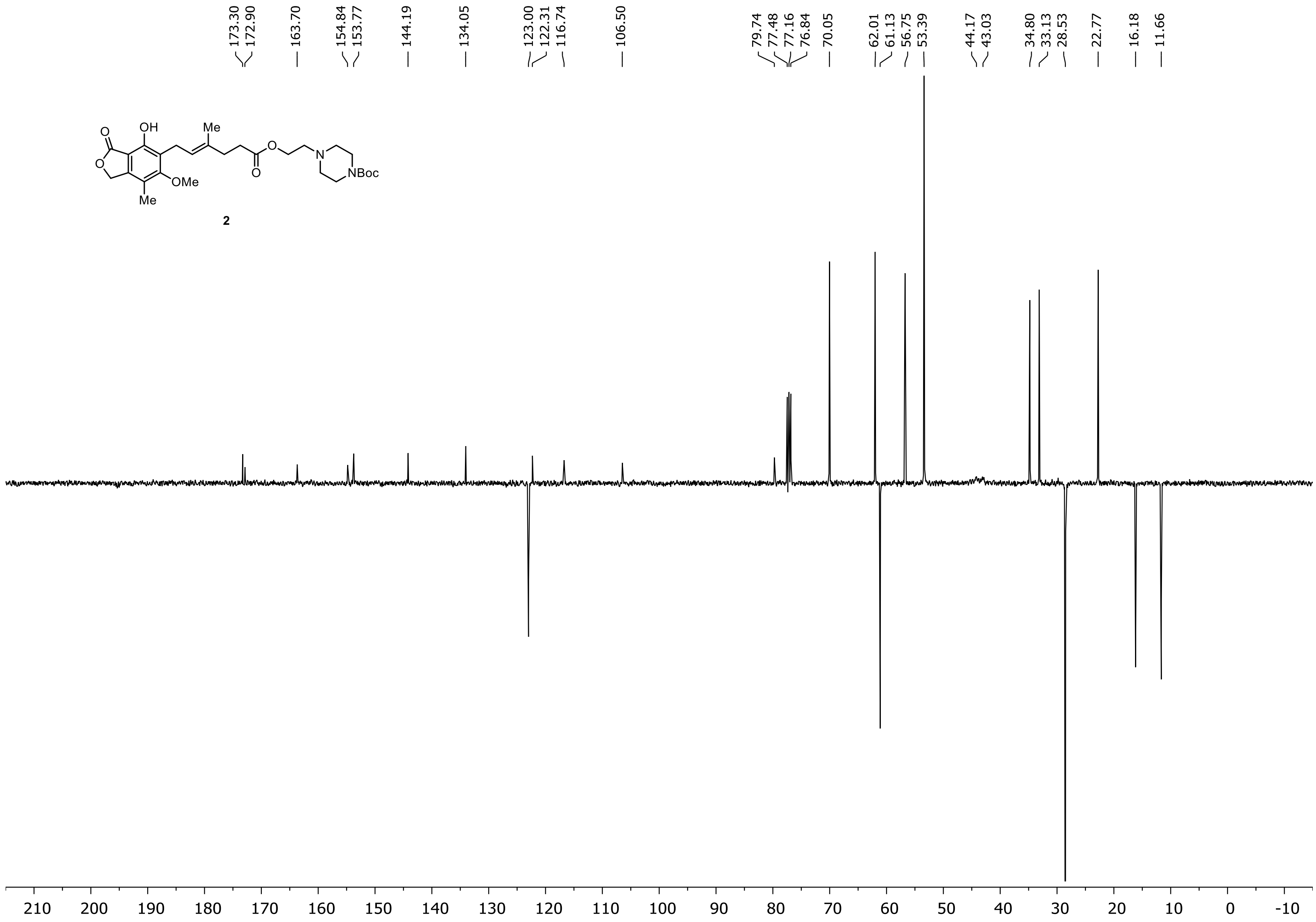

2-(1-Pyrrolidinyl)ethyl (*E*)-6-(4-*tert*-Butyldimethylsilyloxy-6-methoxy-7-methyl-3-oxo-1,3-dihydroisobenzofuran-5-yl)-4-methylhex-4-enoate (**S3**) (^{1}H NMR; 400 MHz; $CDCl_3$)

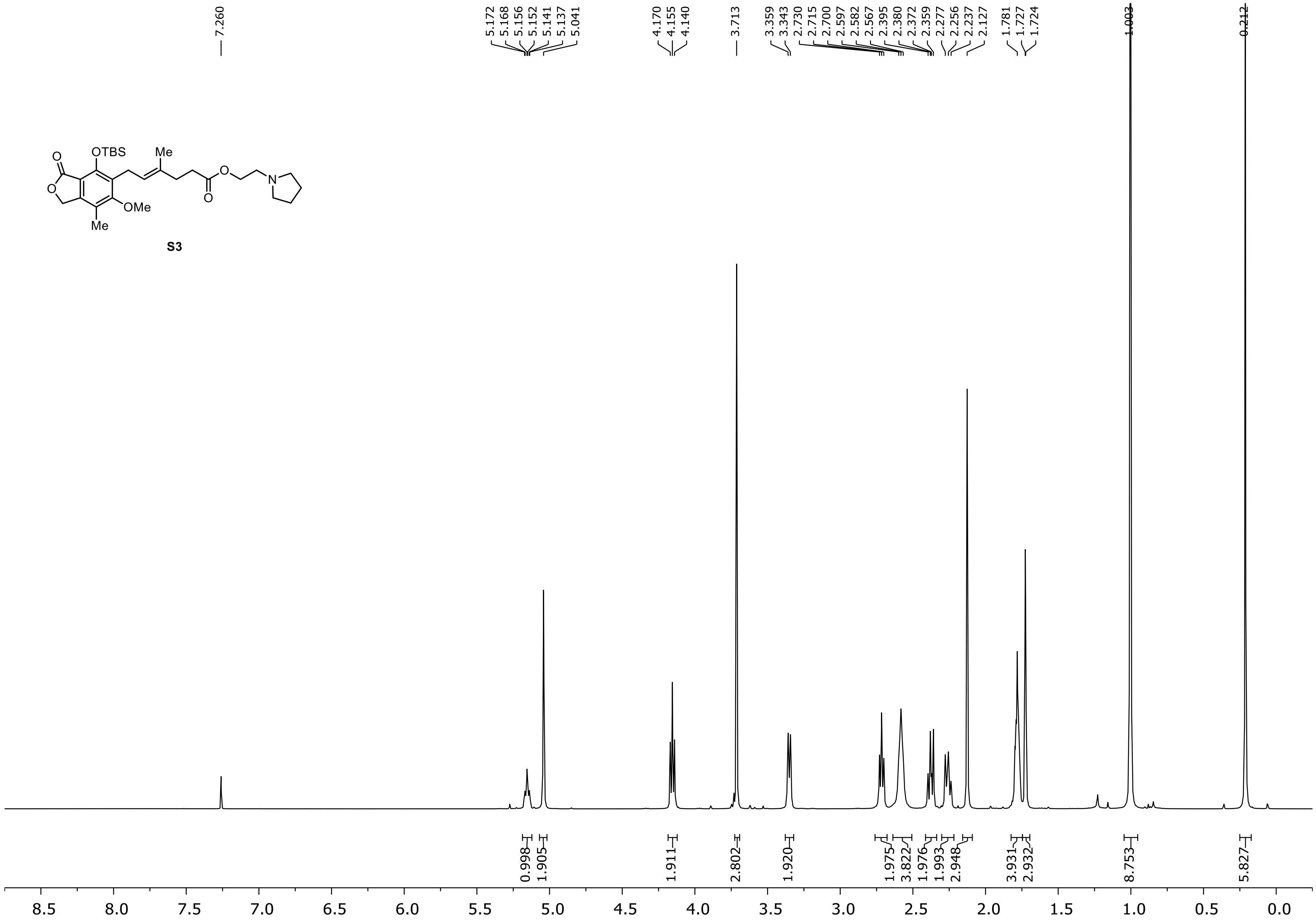

2-(1-Pyrrolidinyl)ethyl (*E*)-6-(4-*tert*-Butyldimethylsilyloxy-6-methoxy-7-methyl-3-oxo-1,3-dihydroisobenzofuran-5-yl)-4-methylhex-4-enoate (**S3**) (^{13}C NMR; 100 MHz; $CDCl_3$)

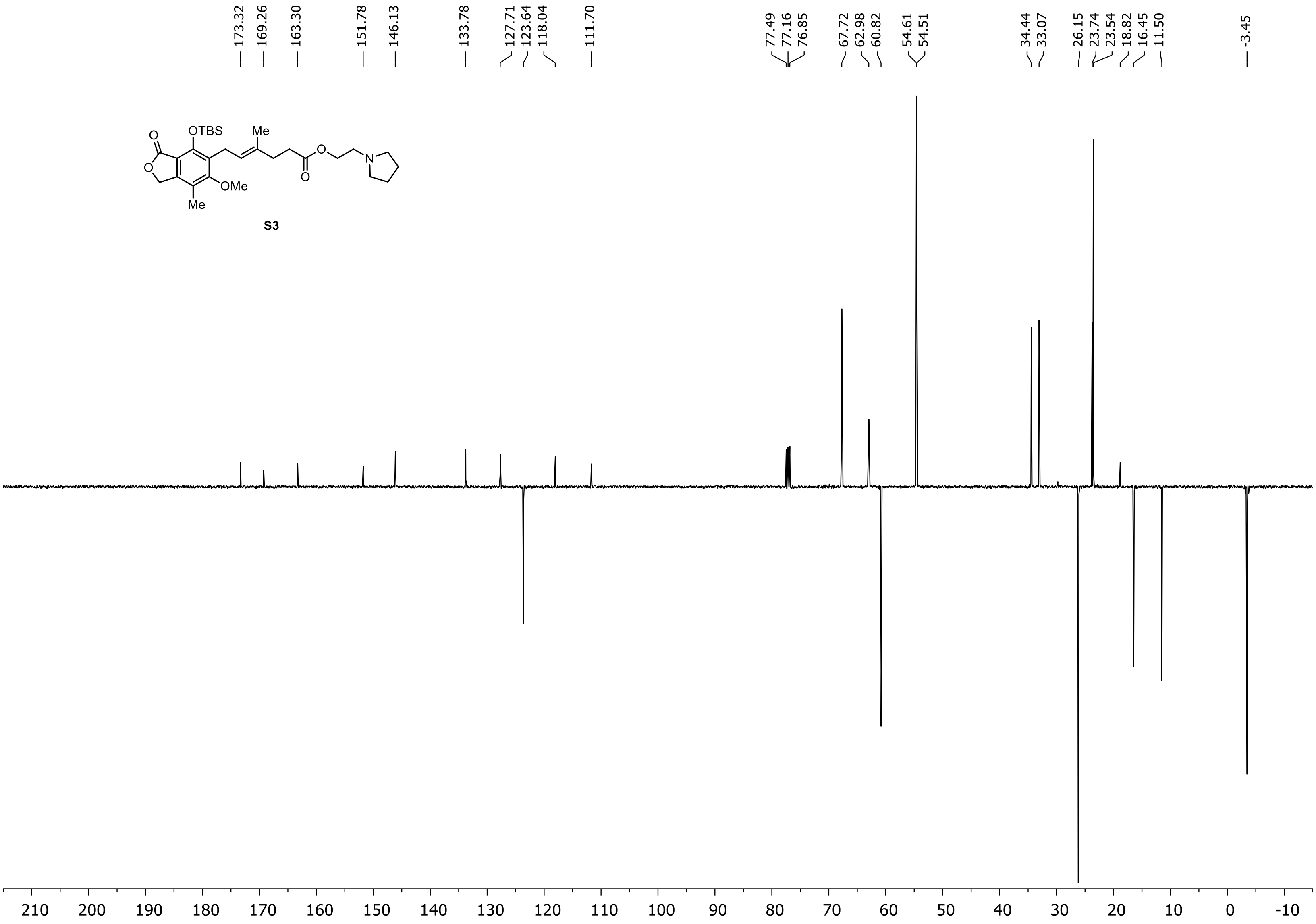

Mycophenolic acid, 2-(1-pyrrolidinyl)ethyl ester (**3**) (^{1}H NMR; 400 MHz; $CDCl_3$)

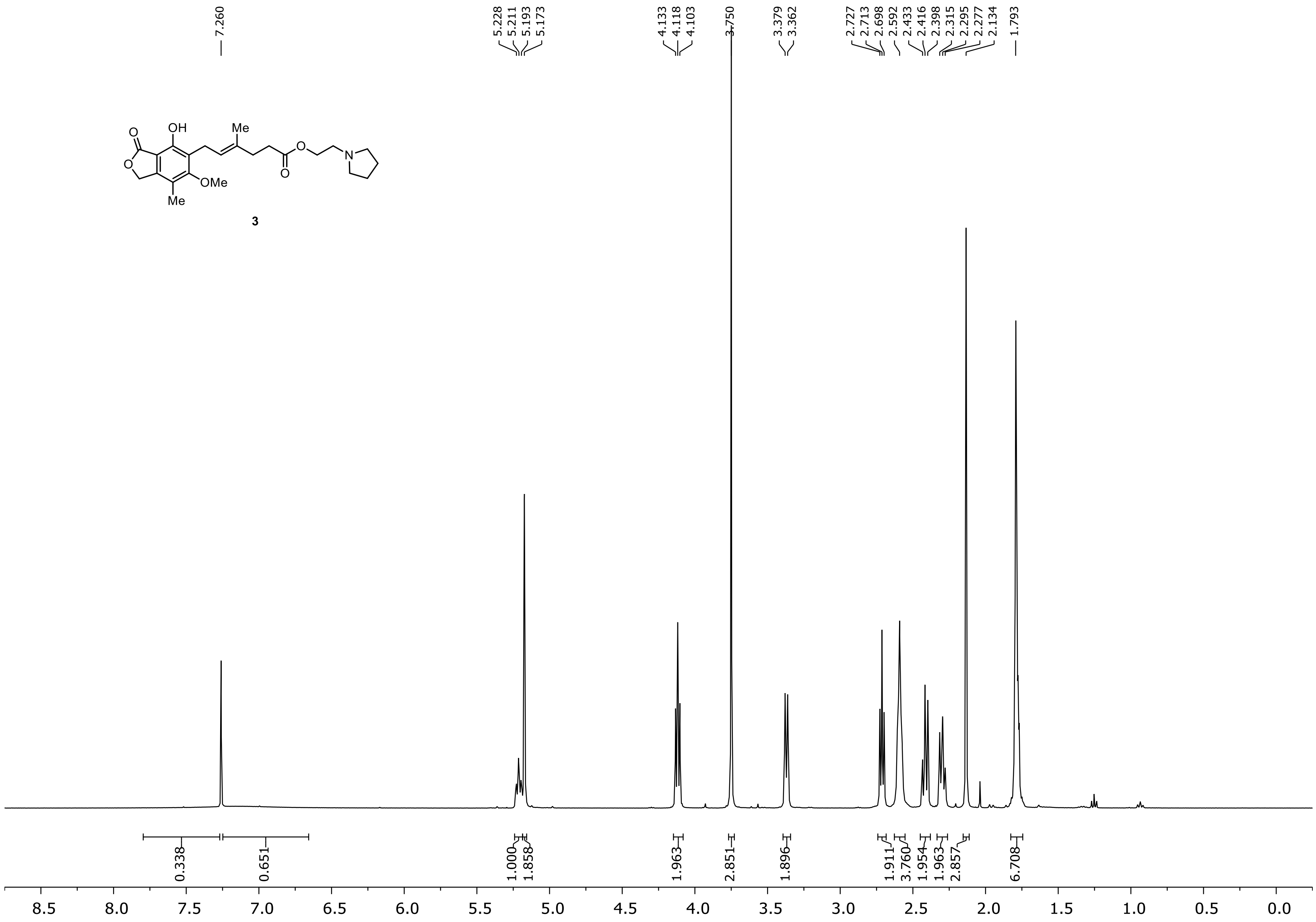

Mycophenolic acid, 2-(1-pyrrolidinyl)ethyl ester (**3**) (^{13}C NMR; 100 MHz; $CDCl_3$)

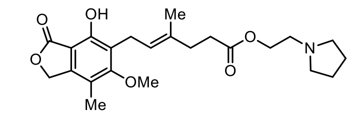

3

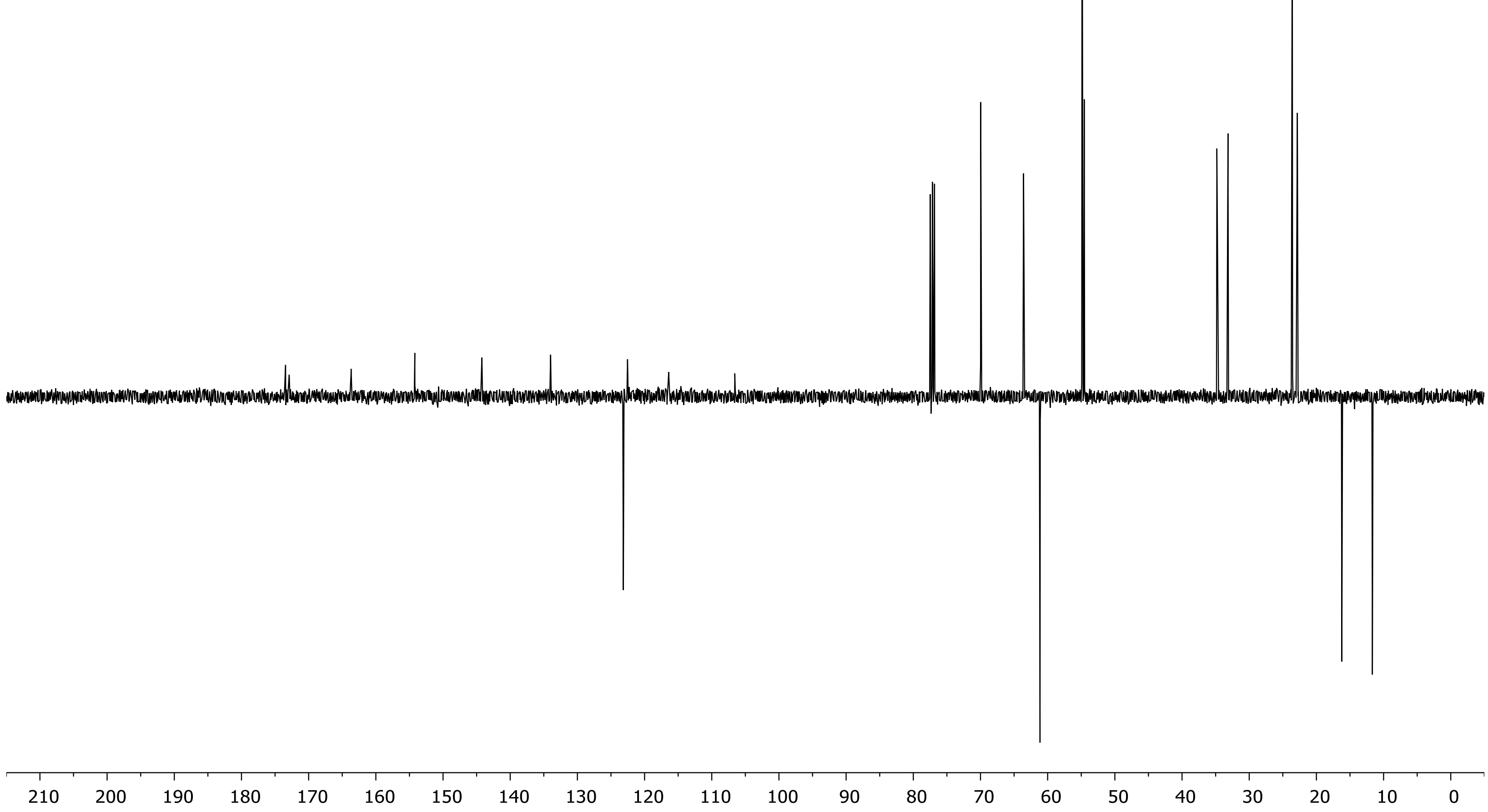

Mycophenolic acid, 4-methylpiperazine amide (**4**) (^{1}H NMR; 400 MHz; $CDCl_3$)

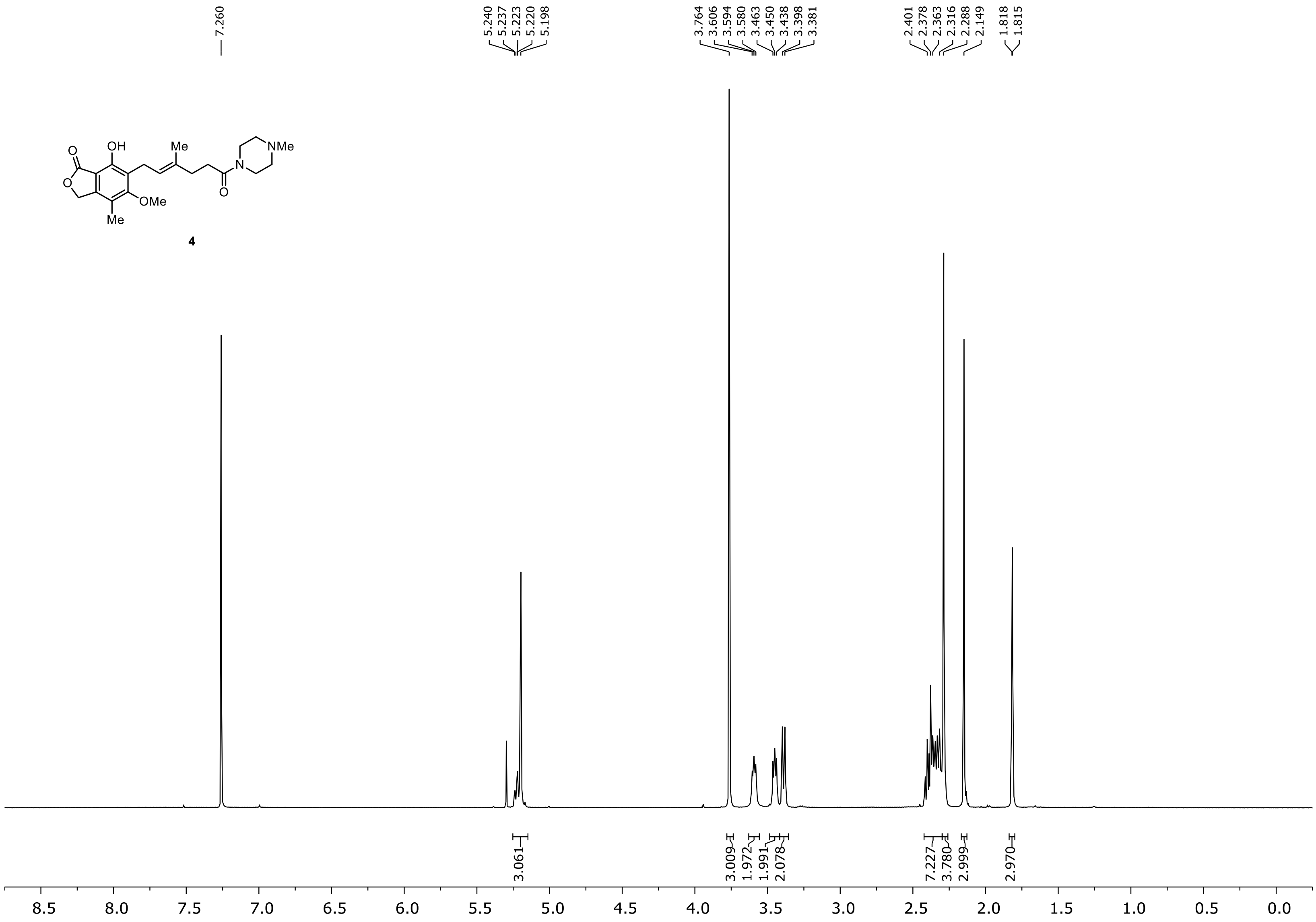

Mycophenolic acid, 4-methylpiperazine amide (**4**) (^{13}C NMR; 100 MHz; $CDCl_3$)

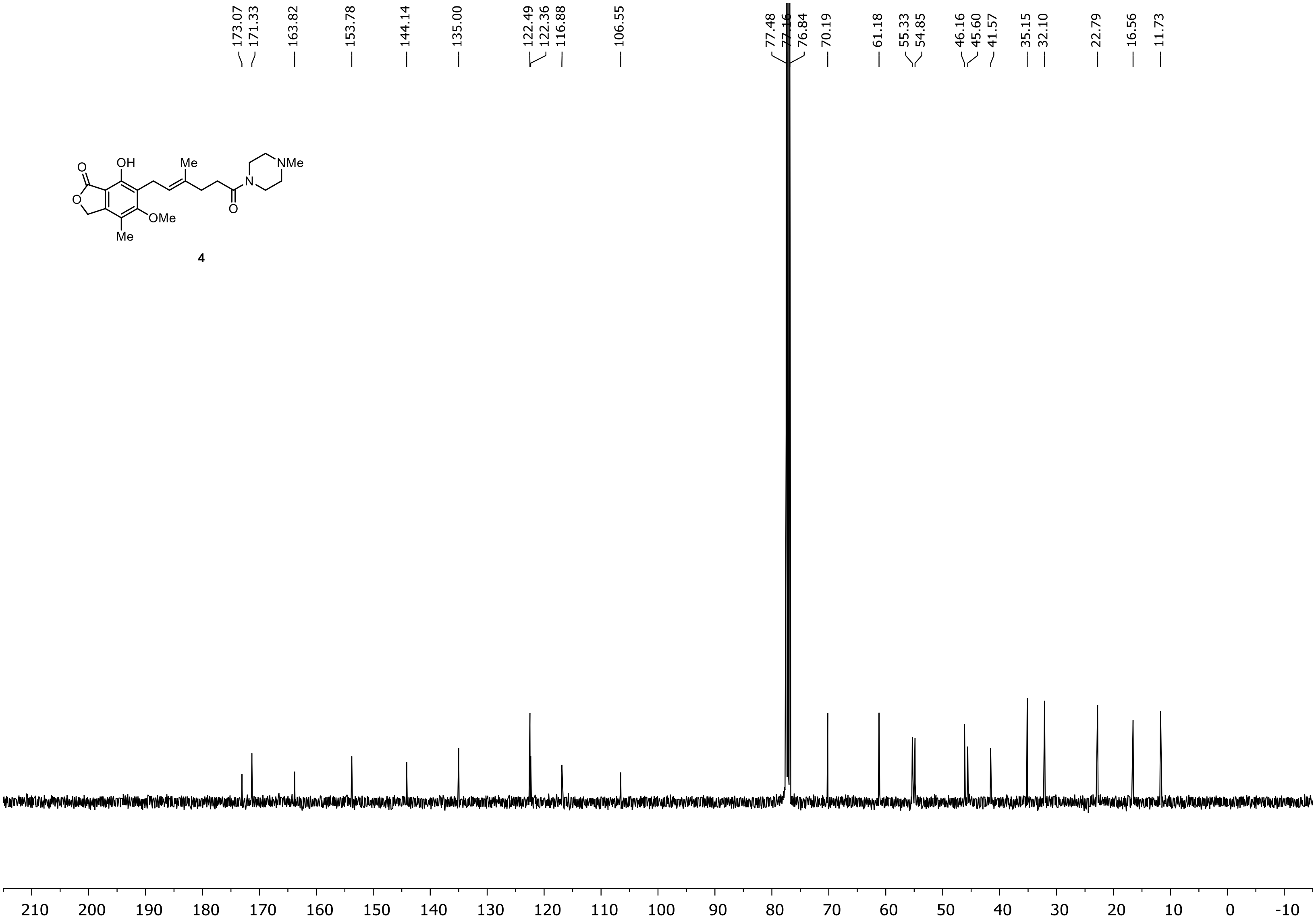

Mycophenolic acid, 4-methylpiperazine amide (**4**) (HSQC; $CDCl_3$)

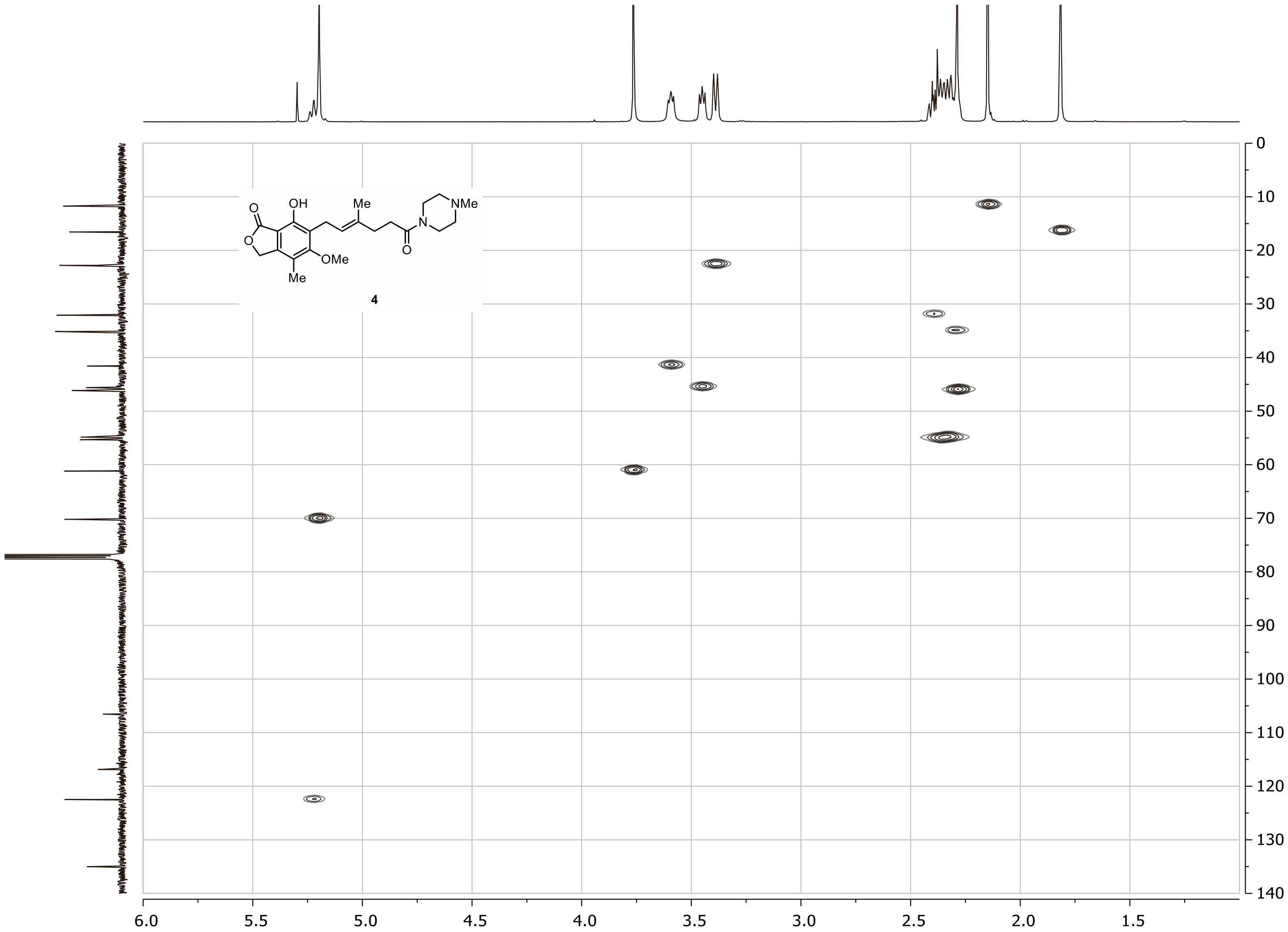

Mycophenolic acid, 4-methylpiperazine amide (**4**) (HMBC; $CDCl_3$)

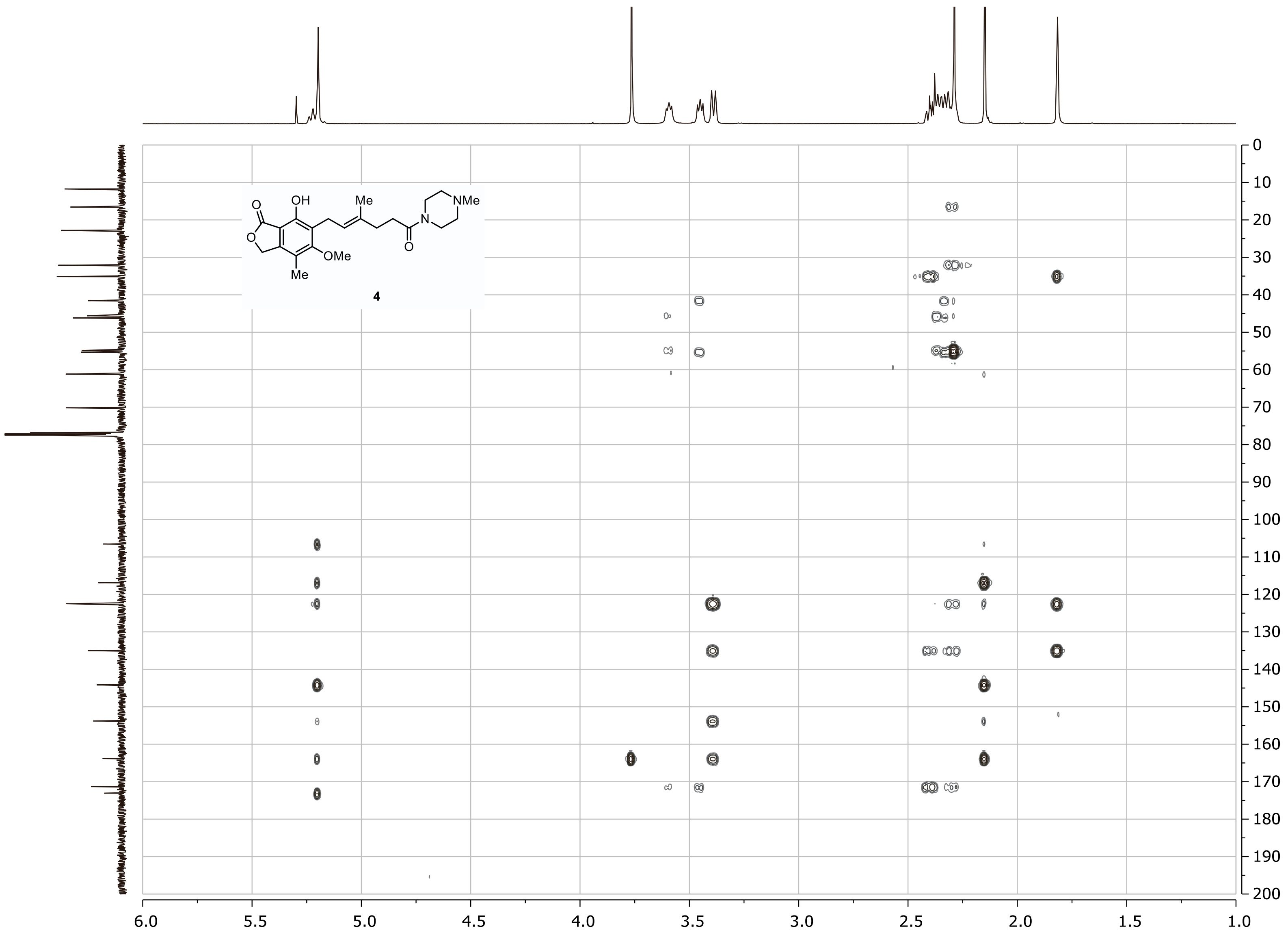

Mycophenolic acid, 3-(azetidin-1-yl)propyl amide (**5**) (^{1}H NMR; 400 MHz; $CDCl_3$)

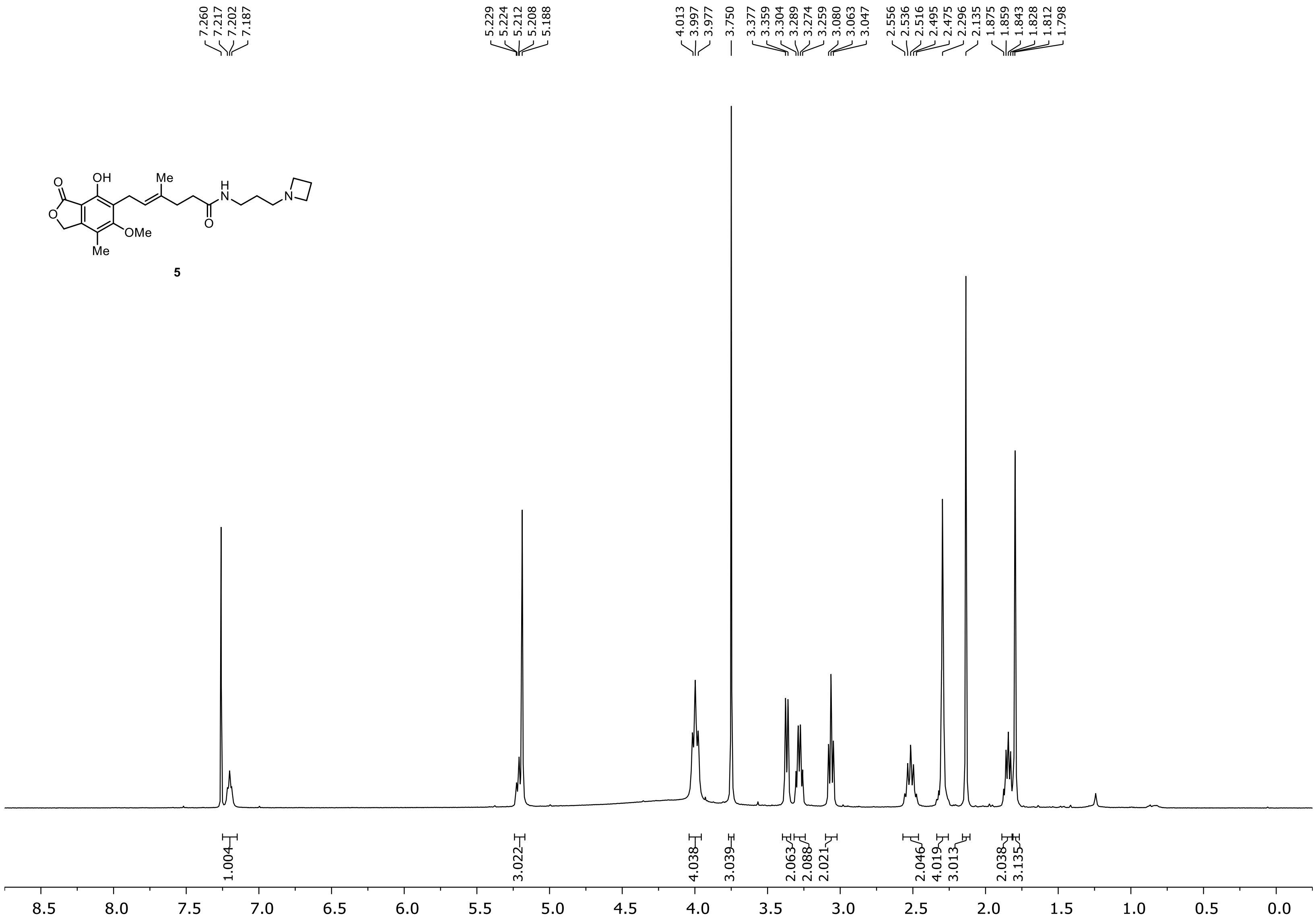

Mycophenolic acid, 3-(azetidin-1-yl)propyl amide (**5**) (^{13}C NMR; 100 MHz; $CDCl_3$)

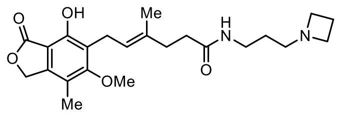

5

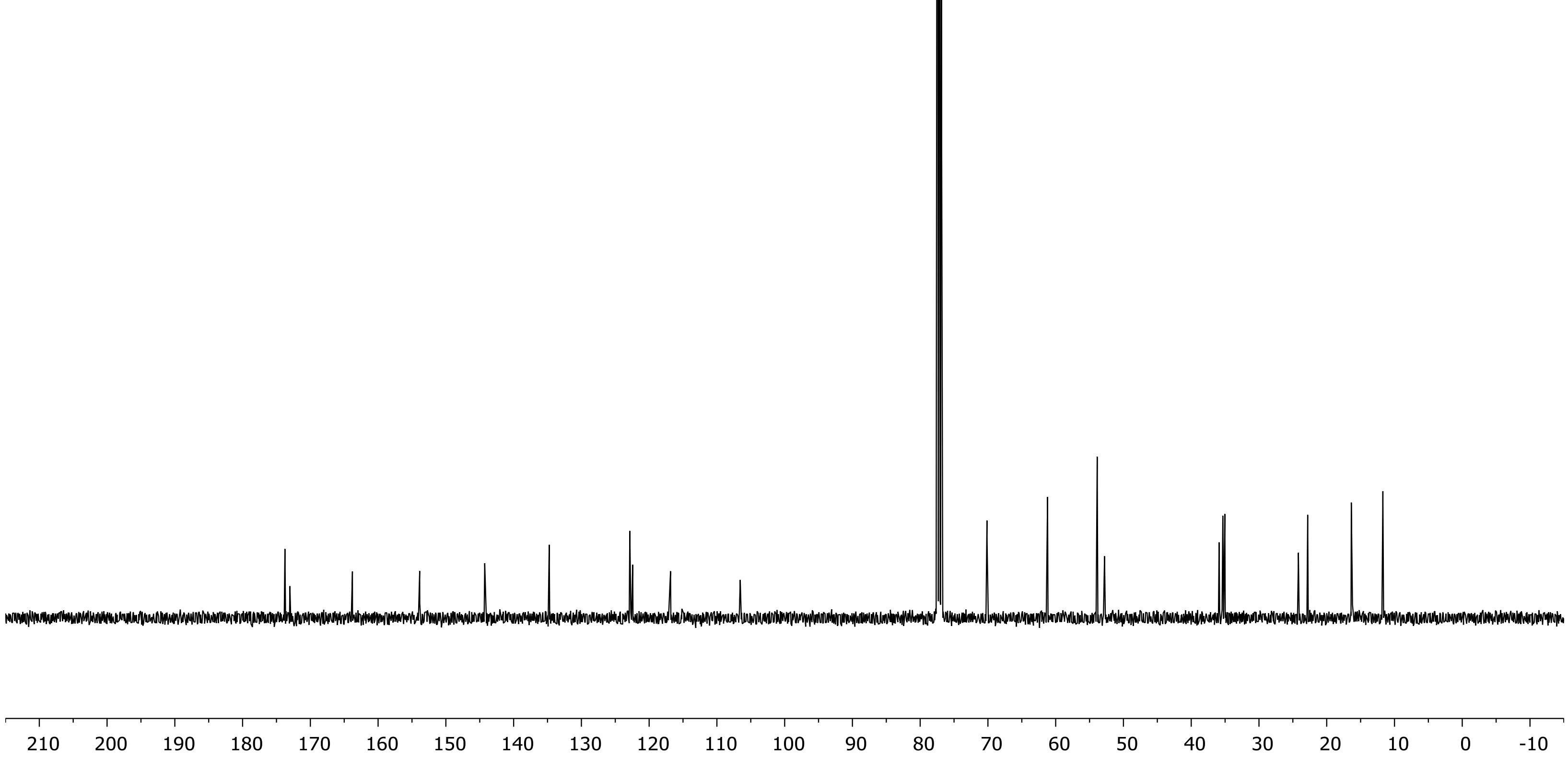

Mycophenolic acid, 3-(azetidin-1-yl)propyl amide (**5**) (HSQC; $CDCl_3$)

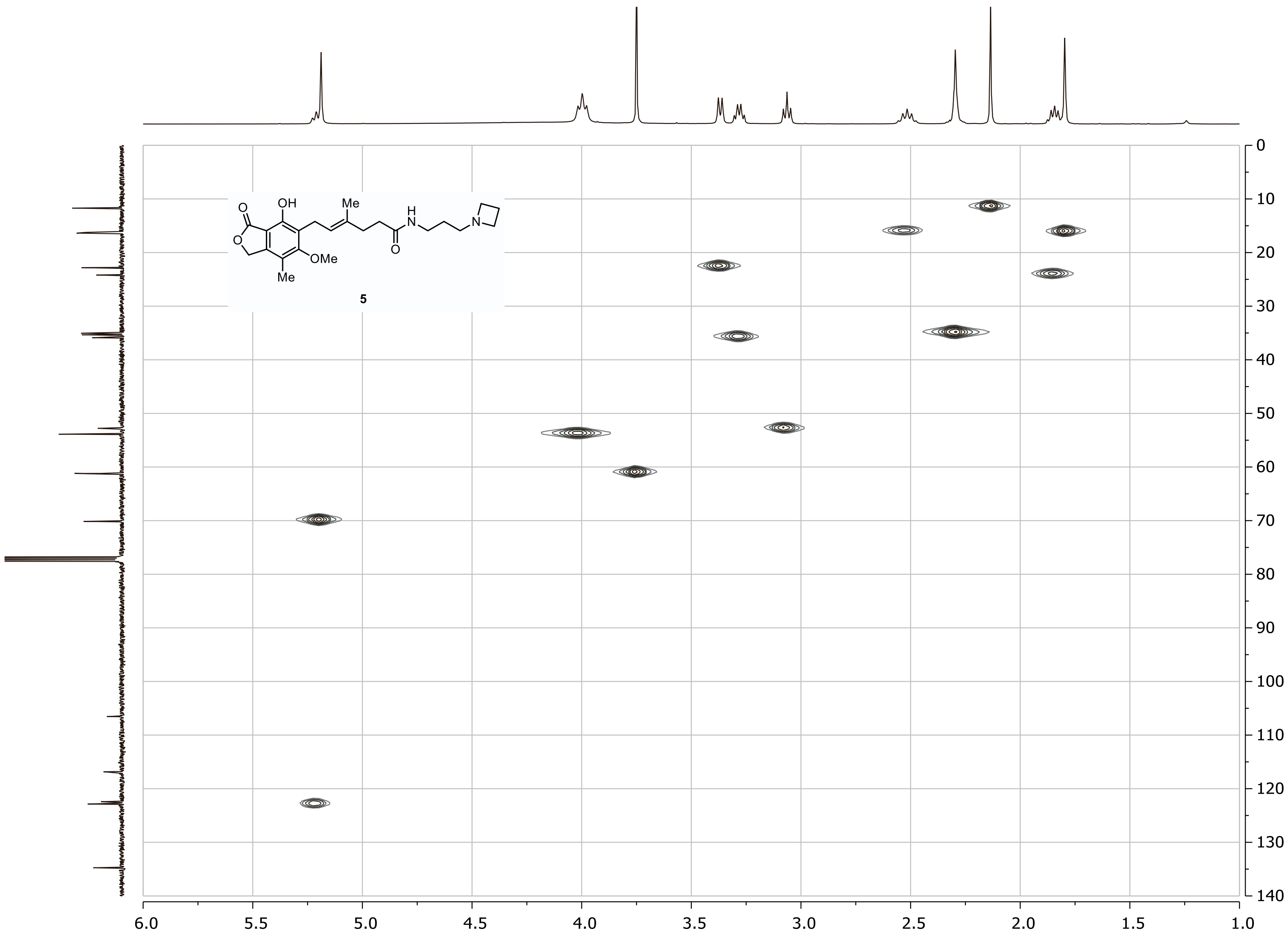

Mycophenolic acid, 3-(azetidin-1-yl)propyl amide (**5**) (HMBC; $CDCl_3$)

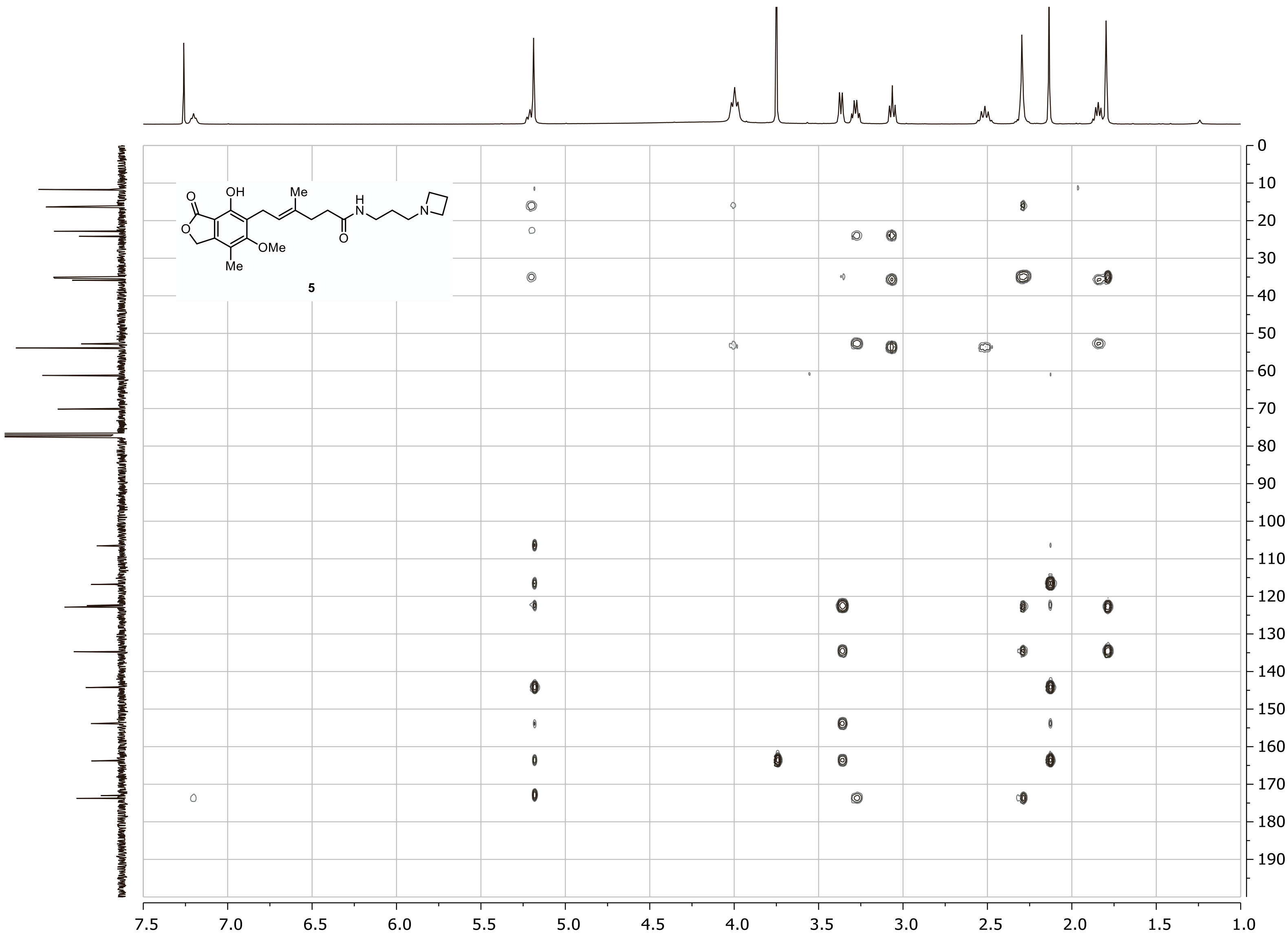

2-(1-Pyrrolidinyl)ethyl (*E*)-6-(4-*tert*-butyldimethylsilyloxy-6-methoxy-7-methyl-3-oxo-1,3-dihydroisobenzofuran-5-yl)-4-methylhex-4-enoate (**S4**) (^{1}H NMR; 400 MHz; $CDCl_3$)

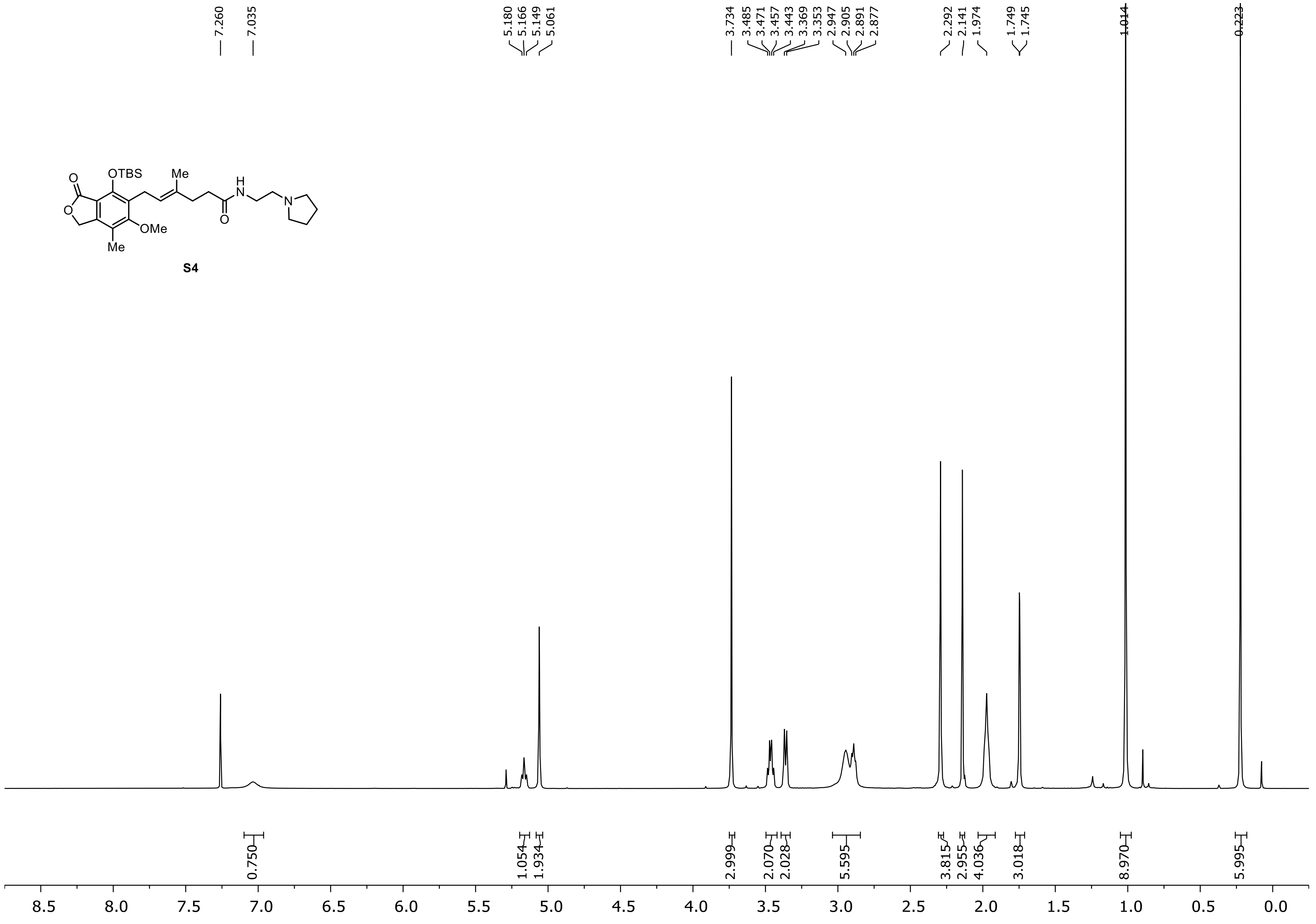

2-(1-Pyrrolidinyl)ethyl (*E*)-6-(4-*tert*-butyldimethylsilyloxy-6-methoxy-7-methyl-3-oxo-1,3-dihydroisobenzofuran-5-yl)-4-methylhex-4-enoate (**S4**) (^{13}C NMR; 100 MHz; $CDCl_3$)

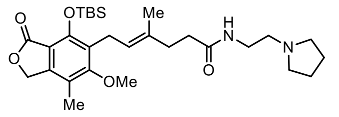

S4

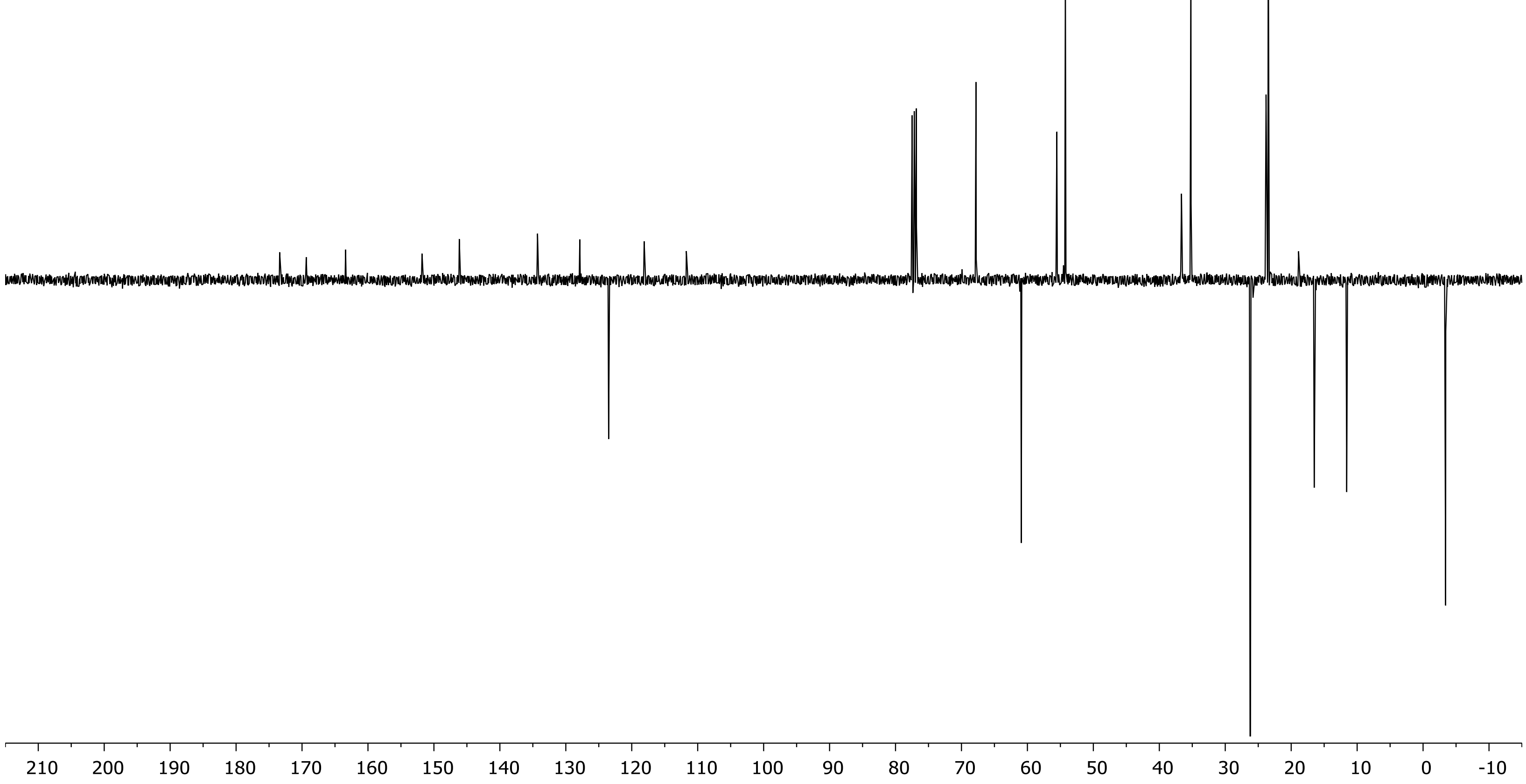

Mycophenolic acid, 2-(1-pyrrolidinyl)ethyl amide (**6**) (^{1}H NMR; 400 MHz; $CDCl_3$)

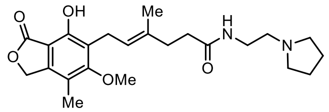

6

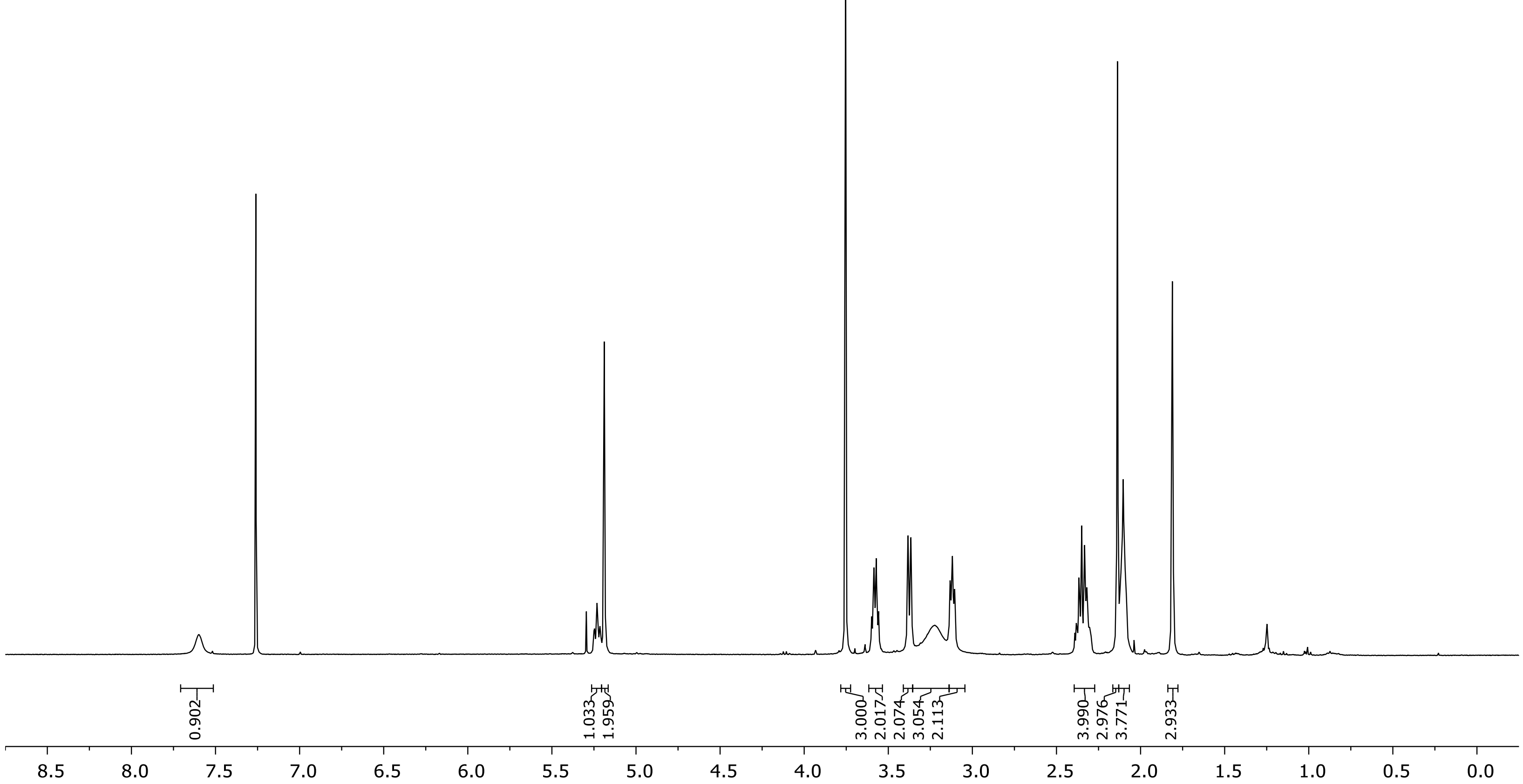

Mycophenolic acid, 2-(1-pyrrolidinyl)ethyl amide (**6**) (^{13}C NMR; 100 MHz; $CDCl_3$)

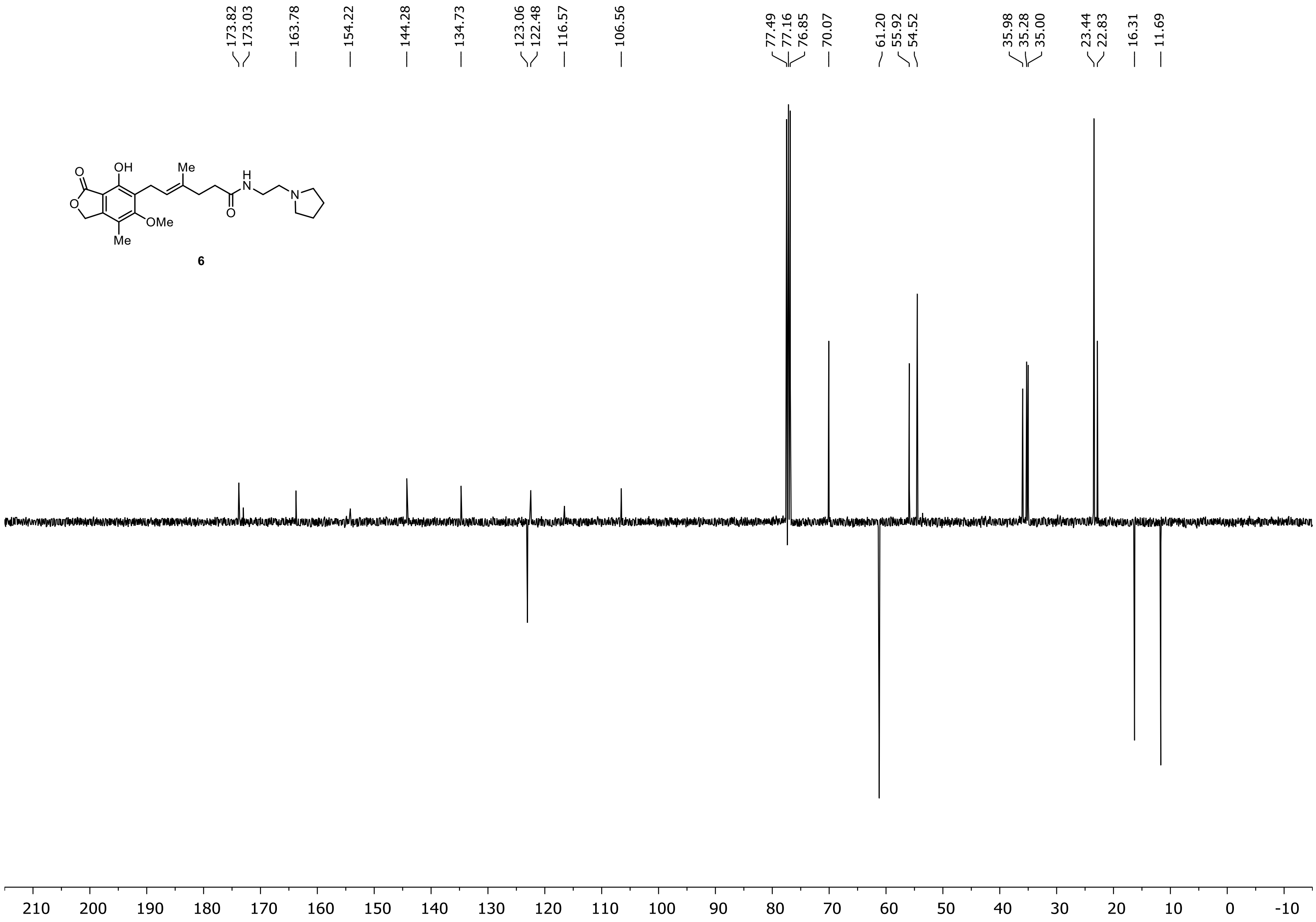

Supplemental References

S1. Cholewiński, G., Iwaszkiewicz-Grześ, D., Prejs, M., Głowacka, A., and Dzierzbicka, K. (2015). Synthesis of the inosine 5′-monophosphate dehydrogenase (IMPDH) inhibitors. J. Enzyme Inhib. Med. Chem. *30*, 550–563. 10.3109/14756366.2014.951349.
