# Supplementary material for: De novo GTP synthesis is a metabolic vulnerability for the interception of brain metastases
Source: Cell Rep Med. 2024 Oct 4;5(10):101755. doi: 10.1016/j.xcrm.2024.101755 (PMC11513854; doi:10.1016/j.xcrm.2024.101755)
Supplement: Document S2. Article plus supplemental information [file mmc6.pdf]

Cell Reports
Medicine

Article

De novo GTP synthesis is a metabolic vulnerability for the interception of brain metastases

Graphical abstract

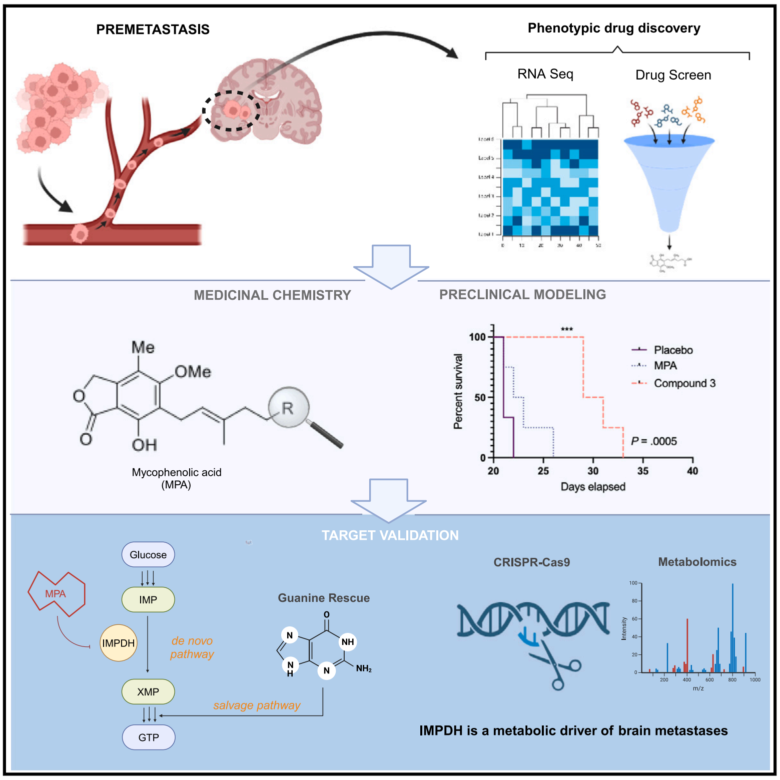

Highlights

- Connectivity Map analysis performed on a pre-metastatic transcriptomic signature
- Drug discovery via phenotypic screen identifies mycophenolic acid
- Targeting IMPDH with mycophenolic acid slows brain metastasis formation
- IMPDH is a potential biomarker for at-risk patients with lung cancer

Authors

Agata M. Kieliszek, Daniel Mobilio, Blessing I. Bassey-Archibong, ..., Chitra Venugopal, Jakob Magolan, Sheila K. Singh

Correspondence

ssingh@mcmaster.ca

In brief

Kieliszek et al. use a phenotypic drug discovery approach to identify IMPDH as a targetable metabolic vulnerability in brain metastasis. Medicinal chemistry efforts suggest that prioritizing the BBB permeability of IMPDH inhibitors enhances their preclinical efficacy. Targeting IMPDH represents an avenue to block brain metastasis formation in at-risk patients with cancer.

Kieliszek et al., 2024, Cell Reports Medicine *5*, 101755
October 15, 2024 © 2024 The Author(s). Published by Elsevier Inc.
https://doi.org/10.1016/j.xcrm.2024.101755

Cell Reports Medicine

CellPress
OPEN ACCESS

Article

De novo GTP synthesis is a metabolic vulnerability for the interception of brain metastases

Agata M. Kieliszek,[1,2] Daniel Mobilio,[1,2] Blessing I. Bassey-Archibong,[1,3] Jarrod W. Johnson,[2] Mathew L. Piotrowski,[2] Elvin D. de Araujo,[4] Abootaleb Sedighi,[4] Nikoo Aghaei,[1,2] Laura Escudero,[1,3] Patrick Ang,[1] William D. Gwynne,[1,3] Cunjie Zhang,[5,6] Andrew Quaile,[5,6] Dillon McKenna,[1,3] Minomi Subapanditha,[3] Tomas Tokar,[7] Muhammad Vaseem Shaikh,[1,3] Kui Zhai,[1,3] Shawn C. Chafe,[1,3] Patrick T. Gunning,[4] J. Rafael Montenegro-Burke,[5,6] Chitra Venugopal,[1,3] Jakob Magolan,[2] and Sheila K. Singh[1,2,3,8,9,*]

[1]Centre for Discovery in Cancer Research, McMaster University, Hamilton, ON, Canada
[2]Department of Biochemistry and Biomedical Sciences, McMaster University, Hamilton, ON, Canada
[3]Department of Surgery, McMaster University, Hamilton, ON, Canada
[4]Centre for Medicinal Chemistry, University of Toronto Mississauga, Mississauga, ON, Canada
[5]Terrence Donnelly Centre for Cellular and Biomolecular Research, University of Toronto, Toronto, ON, Canada
[6]Department of Molecular Genetics, University of Toronto, Toronto, ON, Canada
[7]Krembil Research Institute, University Health Network, Toronto, ON, Canada
[8]X (formerly Twitter): @sheilasinghlab
[9]Lead contact
*Correspondence: ssingh@mcmaster.ca
https://doi.org/10.1016/j.xcrm.2024.101755

SUMMARY

Patients with brain metastases (BM) face a 90% mortality rate within one year of diagnosis and the current standard of care is palliative. Targeting BM-initiating cells (BMICs) is a feasible strategy to treat BM, but druggable targets are limited. Here, we apply Connectivity Map analysis to lung-, breast-, and melanoma-pre-metastatic BMIC gene expression signatures and identify inosine monophosphate dehydrogenase (IMPDH), the rate-limiting enzyme in the *de novo* GTP synthesis pathway, as a target for BM. We show that pharmacological and genetic perturbation of IMPDH attenuates BMIC proliferation *in vitro* and the formation of BM *in vivo.* Metabolomic analyses and CRISPR knockout studies confirm that *de novo* GTP synthesis is a potent metabolic vulnerability in BM. Overall, our work employs a phenotype-guided therapeutic strategy to uncover IMPDH as a relevant target for attenuating BM outgrowth, which may provide an alternative treatment strategy for patients who are otherwise limited to palliation.

INTRODUCTION

Brain metastases (BMs) are ten times more frequent than primary brain tumors,[1] and patients diagnosed with BM face a 90% mortality rate within 4–12 months of their diagnosis.[2] Lung cancer, breast cancer, and melanoma account for 85% of the primary cancers that metastasize to the brain.[1] While the current standard-of-care treatment for BM comprises surgical resection and/or radiation therapy, such therapeutic strategies are palliative, and BM remains ostensibly incurable. Moreover, the incidence of BM is increasing due to better systemic treatment options for primary cancers.[3] While significant progress has been made in understanding the genetic landscape[4–6] in secondary brain tumor formation, there remains a lack of clinically relevant models that can identify therapeutically suitable targets for the treatment of BM.

The bulk of cells within a primary tumor vary in their proliferative, differentiation, and self-renewal capacities, as well as their metastatic capacity. Only 0.01% of metastasizing primary tumor cells are capable of initiating and sustaining a secondary tumor.[7] This cell population is theorized to have inherent stem-like and tumor-initiating properties that drive malignant tumor progression and contribute to drug resistance and relapse.[8–11] We have isolated and characterized this stem-like cell population, termed BM-initiating cells (BMICs), from patient-derived lung-,[12,13] breast-, and melanoma-BM.[14] BMICs evade conventional therapies and migrate away from their primary tumors to the brain to form BM.[15] Therefore, developing therapeutic strategies to prophylactically eradicate BMICs may be a more effective approach than treating existing BM.[9,16]

Using established lung-to-BM models,[12,13] we recently captured a population of lung-BMICs that arrived in the brain and are undetectable by standard imaging techniques, which we termed pre-metastatic BMICs.[13] We applied Connectivity Map (CMap) computational analysis[17] to the transcriptome of pre-metastatic lung-BMICs and identified apomorphine as a candidate compound to block the formation of lung-BM.[13] We have since expanded this model to include breast- and

This is an open access article under the CC BY license (http://creativecommons.org/licenses/by/4.0/).

melanoma-pre-metastatic BMICs and identified a shared gene signature of pre-metastatic BMICs common to all three cohorts.[14]

Here, we utilized this shared pre-metastatic BMIC signature to uncover targetable therapeutic vulnerabilities in lung-, breast-, and melanoma-BMICs by applying CMap analysis. We identified mycophenolic acid (MPA), a known inhibitor of inosine monophosphate dehydrogenase (IMPDH), as a potent suppressor of BMIC activity. Due to MPA's predicted low blood-brain barrier (BBB) permeability, we synthesized a focused library of analogs that are predicted to have greater BBB permeability. Compound **3** possessed superior *in vivo* efficacy against BM compared to MPA by significantly extending survival. Mechanistically, we identified that purine synthesis is a metabolic vulnerability in BM, where pharmacological inhibition of IMPDH slowed the formation of BM. Together, our data show that inhibition of IMPDH should be explored for the treatment and prevention of BM.

RESULTS

CMap analysis of pre-metastatic BMIC gene expression profiles reveals MPA as a selective BMIC inhibitor

Our previous work has led to the successful establishment of preclinical models of lung-, breast-, and melanoma-BM using three different injection routes: (1) orthotopic, (2) intracardiac, and (3) intracranial.[12–14,18] In brief, BM tumors surgically removed from patients with BM are processed and cultured in tumorsphere-enriching media to establish BMIC lines; BMIC lines are subsequently injected into NSG mice via orthotopic (lung, fat pad, or subcutaneous for lung-, breast-, and melanoma-BM, respectively), intracardiac, or intracranial routes.[12–14,18] Most recently, we reported that our orthotopic models of lung-, breast-, and melanoma-BM are able to capture BMICs in their early or "pre-metastatic" stage of BM development where BMICs have seeded the brain but have not yet formed metastatic lesions that are visible by immunohistochemistry.[13,14] We studied the transcriptomic profiles of pre-metastatic lung-, breast-, and melanoma-BMICs by RNA sequencing and found that they are distinct from their BMIC line counterparts (Figures 1A and 1B).[13,14] The term "pre-metastatic BMICs" herein refers to a tumor cell population that has newly arrived in the brain and is undetectable by standard imaging techniques such as MRI or immunohistochemistry.

In this work, we aimed to identify potential druggable targets for BM, by characterizing the transcriptomes of pre-metastatic lung-, breast-, and melanoma-BMICs (see Figure 1A).[13,14] We identified 3,951 genes that are commonly differentially expressed in the pre-metastatic cohort of cells compared to BMICs isolated from established patient tumors (Figure 1C).[14] We input the shared gene signature of pre-metastatic BMICs (defined by the commonly shared gene signature from BMICs of all three primary tumor cohorts) as a query signature for computational CMap[17] analysis to identify compounds that evoke opposing transcriptional changes (see Figure 1A). The goal of using CMap is to generate testable hypotheses about drugs that have not yet been characterized in certain disease contexts, where a query gene signature (i.e., our pre-metastatic gene signature) is compared against a reference database containing signatures representing a change in cellular state in response to a drug, gene, disease, or other perturbation. 380 compounds were suggested by CMap to affect our pre-metastatic signature, of which 194 were predicted to have an "opposing" effect. Only 48 candidate compounds met our established probability value cutoff of 0.05[17] (Figure 1D). To validate these findings, we screened all 48 compounds for their capacity to affect the viability of a patient-derived lung-BMIC line (BT478) at a concentration of 10 μM (Figure 1E). We identified several compounds belonging to diverse chemical families that significantly inhibited BMIC viability. One such compound, apomorphine, has previously been reported by our group to block lung-BM,[13] thus validating our target discovery pipeline.

We fully evaluated the effective compounds based on their prior reports in cancer treatment and potential toxicity for their current indication(s). One of the most important criteria in selecting a lead compound of interest was synthetic tractability since we could not find sufficient evidence of BBB permeability[19,20] for the majority of the compounds and realized that the compound(s) would most likely need to be chemically modified to enhance BBB penetration. We selected the natural product MPA as the lead compound for further study because it possessed a relatively high anti-BMIC activity against multiple patient-derived BMIC lines from lung-, breast-, and melanoma-BM below its clinically relevant plasma C_{max} concentration of 10 μM[21] (Figures 2A and S1), while remaining nontoxic to normal brain cells (Figure 2B) at the same concentrations. Importantly, its chemical structure was observed to be easily manipulated compared to the other CMap hits. Furthermore, MPA inhibits BMIC proliferation over time (Figure 2C) and significantly reduces the frequency of stemness in BMIC lines in both limiting dilution assays (Figure 2D) and clonogenic sphere formation assays (Figure 2E), which are *in vitro* surrogate measures for stem cell self-renewal.[22,23] This suggests that MPA is targeting the stem-like properties of BMICs that are presumed to drive their tumor-initiating properties, which allow them to evade conventional therapies.[15] Finally, using an *in vitro* wound healing assay, we determined that MPA inhibits the migration of patient-derived BMICs (Figures 2F and 2G, Videos S1, S2, S3, and S4), suggesting that MPA targets phenotypes relevant to the metastatic tumor initiation cascade.[24,25]

To the best of our knowledge, neither MPA nor its known target, IMPDH, has been previously implicated in BM. Notably, MPA is predicted to have low BBB penetrance based on *in silico* analyses and has not been previously considered for preventing brain cancer. Nonetheless, due to its selectivity toward BMIC inhibition over normal brain cell controls *in vitro* and the lack of targeted therapies for BM that can extend patient survival, we aimed to utilize MPA as a tool compound to uncover targets for BM research while developing BBB-permeable analogs of MPA to confirm the target's therapeutic relevance preclinically.

MPA slows BM progression in an *in vitro* pre-treatment PDX model

We next determined whether MPA can impact the ability of BMICs to recapitulate BM *in vivo* using our established BM patient-derived xenograft (PDX) models.[14] We decided to focus primarily on lung-BM for our *in vivo* experiments because they

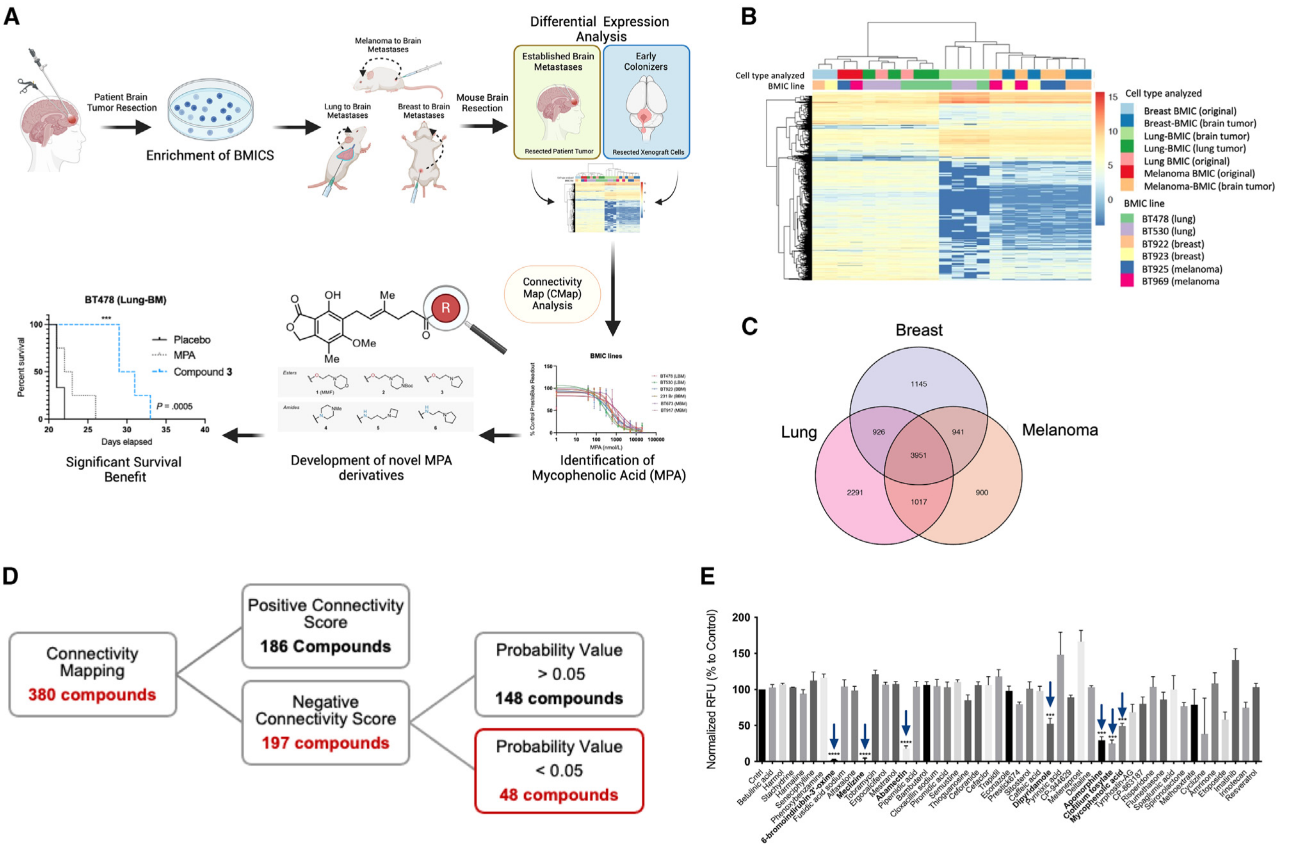

Figure 1. Phenotypic screen identifies anti-BMIC compounds

(A) Schematic of phenotypic screen pipeline. Transcriptomes of BMICs isolated from established patient BM samples were compared to the same BMICs isolated from the brains of mice following orthotopic transplantation (i.e., early colonizers of metastatic spread). Created with BioRender.com.

(B) RNA sequencing revealed a unique molecular and genetic profile suggestive of deregulation during the pre-metastatic stage of BM from all three cohorts. "Original" denotes BMIC samples collected prior to xenograft injection.

(C) Venn diagram of 3,951 genes being commonly differentially expressed during pre-metastasis.

(D) Schematic of selection criteria that led to 48 compounds being chosen for preliminary drug screen and evaluated against an in-house patient-derived lung-BMIC line at 10 μM for 72 h using a PrestoBlue readout.

(E) 48 CMap compounds evaluated against an in-house patient-derived lung-BMIC line; seven compounds significantly decreased the viability to lung-BMICs (BT478) compared to vehicle control after a 72-h incubation period (blue arrows refer to compounds shown in Figure 1D; $n = 3$; ***, $p < 0.001$; ****, $p < 0.0001$). See also Figure S1. Comparisons of cell viability were made via a two-tailed unpaired t test and data are presented as mean ± SD from 3 technical replicates.

account for over 50% of all BM cases. To assess whether MPA treatment would affect brain tumor formation while keeping in mind that MPA's BBB penetrance is poor, BMICs were pre--treated *in vivo* with either MPA (at its 80% maximal inhibitory concentration; IC_{80}) or a placebo. A clonogenic secondary sphere formation assay (Figure 3A) showed that MPA-treated BMICs do not regain sphere-forming capability following MPA removal from the culture media, suggesting that MPA's effect on BMICs is either irreversible[26,27] or that MPA is targeting an important pathway for BM formation that warrants further investigation (Figure 3B). Thereafter, following *in vitro* pre-treatment of BMICs with MPA or DMSO control, equal numbers of viable tumor cells were orthotopically engrafted into immune-compromised mice (Figure 3C). Mice engrafted with MPA-treated BMICs showed a significantly reduced brain tumor burden 2 weeks post- injection (Figures 3D and 3E) and survived significantly longer (Figure 3F) than mice injected with placebo-treated BMICs. This phenotype was also recapitulated in a melanoma-BM PDX model (Figures S2A and S2B).

To obtain a clinically relevant correlate to the data aforementioned, we acquired two patient-derived lung adenocarcinoma samples: one that was derived from a patient who developed BM following their primary lung tumor diagnosis (CRUK0748), and one that was derived from a patient who, to-date, has not developed BM (CRUK0733). BM-initiating capacity was confirmed in our PDX models (Figure S1C). We harvested the tagged CRUK0748 cells from the brains of mice and cultured them in stem cell-enrichment media conditions to derive a CRUK0748-BM line. MPA was tested against CRUK0748-BM in a dose-response assay, which confirmed that MPA inhibits BMIC proliferation within the primary CRUK0748 lung sample (Figure S1D).

Once we saw that MPA affected BM initiation in the brain following *in vivo* pre-treatment (see Figures 3A–3D), we set out to examine whether MPA could inhibit metastasis during the

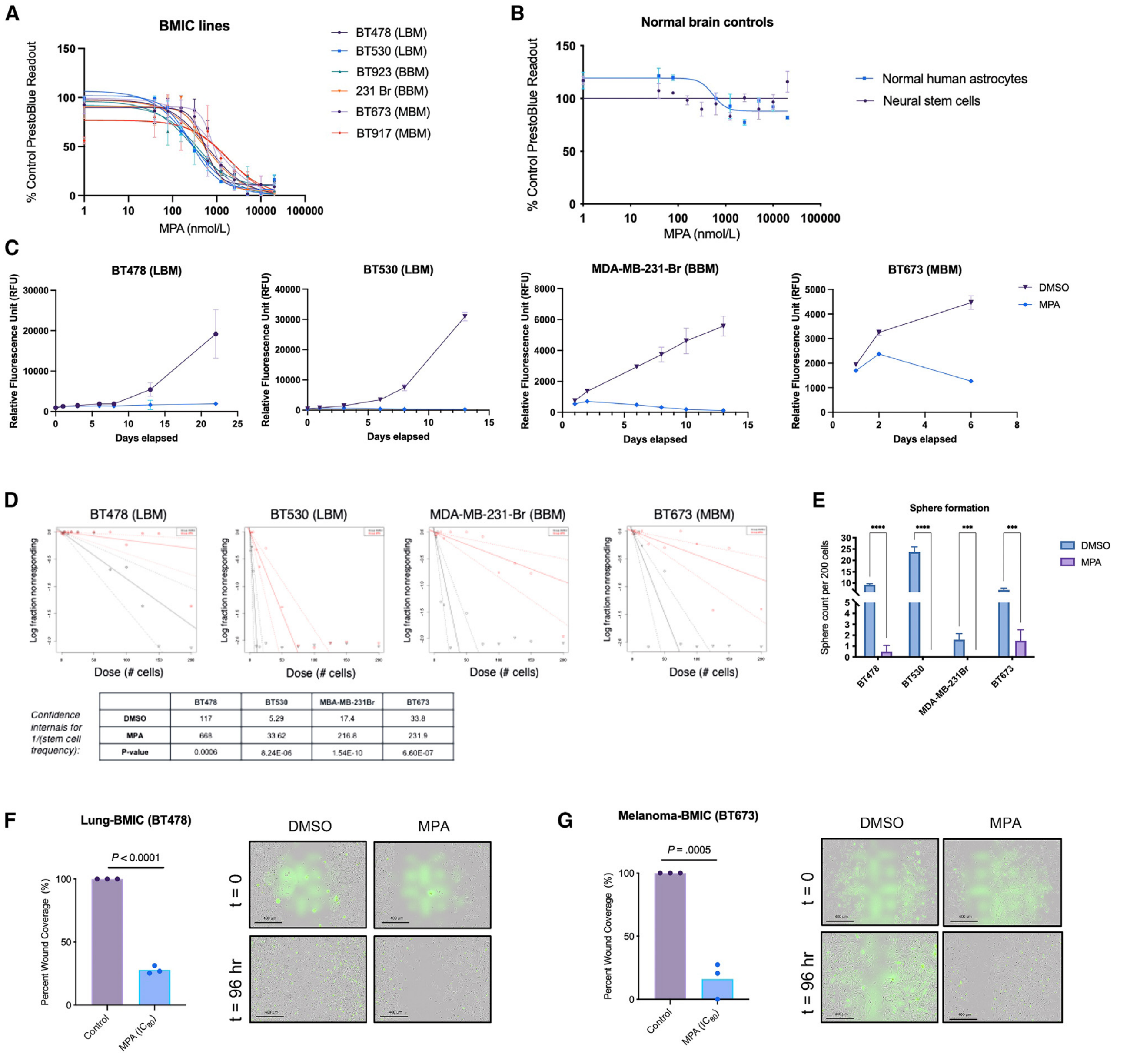

	BT478	BT530	MBA-MB-231Br	BT673
DMSO	117	5.29	17.4	33.8
MPA	668	33.62	216.8	231.9
P-value	0.0006	8.24E-06	1.54E-10	6.60E-07

Figure 2. MPA is a selective anti-BMIC inhibitor

(A) Dose-response curves of multiple lung-BM (LBM), breast-BM (BBM), and melanoma-BM (MBM) cells, and (B) control normal brain cells after a 72-h treatment with MPA. PrestoBlue readout is normalized to vehicle-treated cells. Data are presented as mean ± SD from 4 technical replicates.

(C) Assessment of cell viability of patient-derived BMICs treated with MPA or its vehicle. PrestoBlue readout is normalized to vehicle-treated cells, $p < 0.0001$.

(D) Limiting dilution analysis regression curves of patient-derived BMICs after a 6-day treatment with MPA or its vehicle: plotted using the extreme limiting dilution program (available from: http://bioinf.wehi.au/software/elda/).

(E) Quantification of tumor spheres formed by patient-derived BMICs after 72-h treatment with MPA or its vehicle. Sphere count normalized to vehicle-treated cells, $p < 0.0001$.

(F and G) Percent wound closure of DMSO control vs. MPA-treated cells expressed as an average of replicates ($n = 3$) and images are taken at 10× magnification. Wound closure (represented by dotted white line) is measured using ImageJ on Incucyte-derived images, p values are indicated. Scale bars are 400 μm. See also Videos S1 S2, S3, and S4. Comparisons of cell viability, sphere formation, and wound closure were made via a two-tailed unpaired t test and data are presented as mean ± SD from 3 to 4 technical replicates. SYTOX green nucleic acid stain indicates cell death.

earlier stages of the metastatic cascade. To determine whether MPA slows the spread of metastasizing BMICs from a primary tumor to the brain, we pre-treated primary CRUK0748 lung tumor cells *in vitro* with either MPA or vehicle control before injecting equal numbers of live cells into the intrathoracic cavity of mice (see Figure 3A). Since mice succumb to their primary tumor burden prior to the formation of a macroscopic brain tumor, we collected all brains at the humane endpoint and sorted the cells

CellPress
OPEN ACCESS

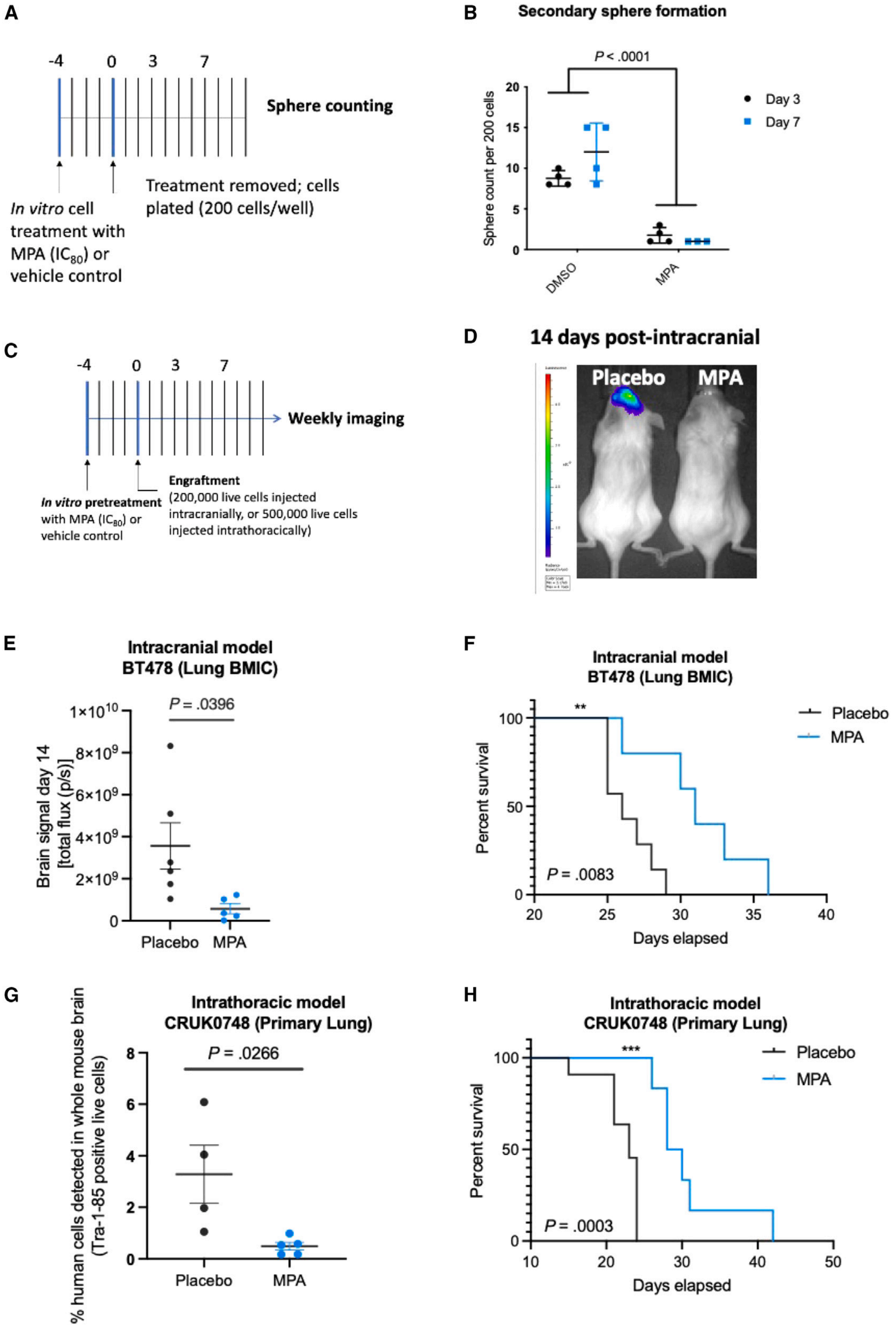

(legend on next page)

for the human cell marker TRA-1-85 by flow cytometry to assess MPA activity on metastasis. Mice orthotopically injected with cells that were pre-treated with MPA had significantly fewer TRA-1-85-positive human cells detected in their brains at the humane endpoint (Figure 3H). Mice injected with MPA-treated cells also experienced a significant increase in median survival time of 6 days compared to the mice injected with vehicle-treated cells (Figure 3H). Taken together, the data obtained from these *in vivo* studies demonstrate that metastatic brain tumor formation is significantly slowed following a short *in vitro* pre-treatment of BMICs with MPA.

BBB penetrance is essential for a BM preventative therapy

We next wondered whether a drug used to slow down, or block, BM needed to be BBB penetrant and aimed to uncover whether MPA could target pre-metastatic BMICs in the circulation in a more clinically relevant *in vivo* treatment model. To this end, we intracardiacally injected mice with patient-derived lung-BMICs and began treating them daily with either MPA or placebo by oral gavage (Figure S3A). MPA-treated mice had a significantly reduced brain tumor burden 7 days post-injection (Figure S3B), suggesting that the BMICs were being effectively targeted outside of the brain cavity. However, a difference in brain tumor burden was no longer observed 14 days post-injection (Figure S3C), and all mice reached the humane endpoint at a similar time point, regardless of treatment (Figure S3D). These results suggest that MPA targets peripheral, but not central, BMICs due to its limitation of crossing the BBB.

Next, we injected mice orthotopically with the primary lung CRUK0748 cell line and treated them daily as described earlier (see Figure S3A*)*. In this model, MPA-treated mice survived significantly longer than their placebo-treated counterparts, suggesting that MPA slowed the growth of their primary lung tumors (Figure S3E). Once the humane endpoint was reached, there was no significant difference in the number of human cells detected in mouse brains as determined by flow cytometry (Figure S3F). While this contradicts our results from our pre-treatment model, we speculate that BMICs are not targeted by MPA treatment once they penetrate the BBB to seed the brain. These data suggest that BBB penetrance is an important limitation to MPA's ability to slow BM and therefore an essential property of a potential anti-BM therapy.

Design, synthesis, and *in vivo* validation of BBB-permeable MPA derivatives

We hypothesized that the poor BBB penetration of MPA is most likely due to the carboxylic acid functionality (pK_a ~5), since it is well known that CNS-active drugs are usually neutral or basic (pK_a 7.5–10.5).[19,20] A common strategy for improving the permeability of carboxylic acids across membranes is via masking the charge through ester prodrugs, which have improved lipophilicity and are cleaved by cellular esterases *in vivo*.[28] The 2-morpholinoethyl ester of MPA, which is called mycophenolate mofetil or MMF (**1**), was developed to improve the oral availability of MPA and has been in clinical use since 1995.[29–31] Here, we synthesized a series of MPA derivatives (Figure 4A and supplementary files) and tested their BBB permeability, with side chains bearing tertiary amines of the appropriate basicity to improve both permeability and solubility. We sought to compare the activities of the esterase-cleavable esters (**1**–**3**) with more metabolically stable amide derivatives (**4**–**6**), since MPA amides have been reported as IMPDH inhibitors previously,[32,33] albeit never designed to prioritize BBB permeability.

We evaluated each analog's selectivity for BMICs using patient-derived lung-BMIC lines (BT478) and neural stem cells as a normal brain cell control (Figure 4B). One of the analogs, Compound **3**, was selected as the superior MPA analog for *in vivo* preclinical study because it retained MPA's therapeutic window in its selectivity for BMICs compared to normal brain cells (Figure 4C) and was suggested to be BBB penetrant using the *in vitro* parallel artificial membrane permeability assay (PAMPA,[34,35] Figure 4D). To explore active transport/efflux mechanisms, we used an MDCK-MDR1 cell monolayer model to show that both MPA and Compound **3** have a high permeability for entering the brain and are not significant substrates for P-glycoprotein (ABCB1), which is known to play a key role in limiting small molecules from entering the brain (Table S1).[36,37] Additionally, the similar efflux ratio results between MPA and Compound **3** in MDCK-MDR1 assays suggest that the ester linkage does not substantially alter the active transport mechanisms but most likely masks the negative charge to facilitate passive uptake. Finally, to explore if there is differential partitioning of either Compound **3** or MPA in brain homogenate, we explored free and bound drug concentrations. The composition of plasma and brain is quite different (>20-fold more lipids in the brain, >2-fold more protein in the plasma), and mouse brain homogenate was treated with each compound followed by

Figure 3. Short exposure of BMICs to MPA slows BM progression in mice
(A) Schematic of clonogenic secondary sphere formation assay.
(B) Quantification of secondary tumor sphere formation by patient-derived BMICs following the removal of MPA (IC_{80} treatment for 72 h) or its vehicle from the culture media. Comparisons were made via a two-tailed unpaired t test and data are presented as mean ± SD from 4 to 5 technical replicates. *p* value is indicated.
(C) Schematic of experimental timeline. BMICs or primary lung cancer cells were pre-treated with MPA or its vehicle *in vivo* (due to MPA's predicted poor BBB penetrance) for 4 days followed by either intracranial or intrathoracic engraftment into immunocompromised mice, respectively.
(D) Representative IVIS bioluminescence images of mice 14-day post injection and (E) brain tumor burden comparisons between placebo and MPA groups. IVIS, *in vivo* imaging system. Comparisons were made via a two-tailed unpaired t test and data are presented as mean ± SD from 4 to 5 technical replicates. *p* value is indicated.
(F) *Kaplan-Meier* survival analysis of placebo and MPA groups following intracranial engraftment of patient-derived BMICs. Comparisons were made via a log rank (Mantel-Cox*)* test, *p* value is indicated.
(G) Quantification of human cells in mouse brains by flow cytometry at the humane endpoint. Comparisons were made via a two-tailed unpaired t test and data are presented as mean ± SD from 4 to 5 technical replicates. *p* value is indicated.
(H) *Kaplan-Meier* survival analysis of vehicle and MPA groups following intrathoracic engraftment of patient-derived metastatic lung tumor cells. Comparisons were made via a log rank (Mantel-Cox) test, *p* value is indicated.

CellPress
OPEN ACCESS

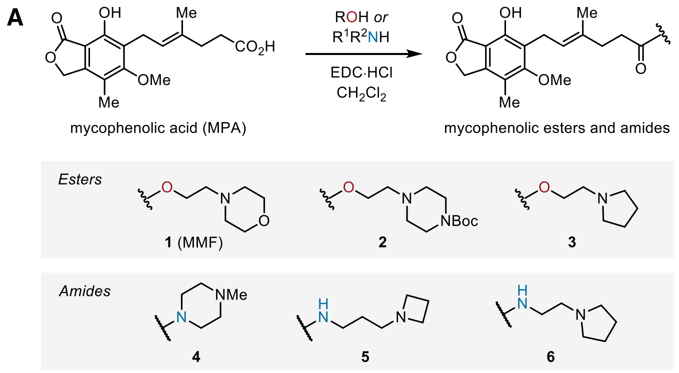

B

MPA analogue	Lung-BM IC50 (μM)	Neural stem cell IC50 (μM)
MPA	3.61	N/A
MMF (**1**)	48.0	31.2
**2**	17.1	353
**3**	15.3	N/A
**4**	N/A	493
**5**	N/A	182
**6**	24.7	70.8

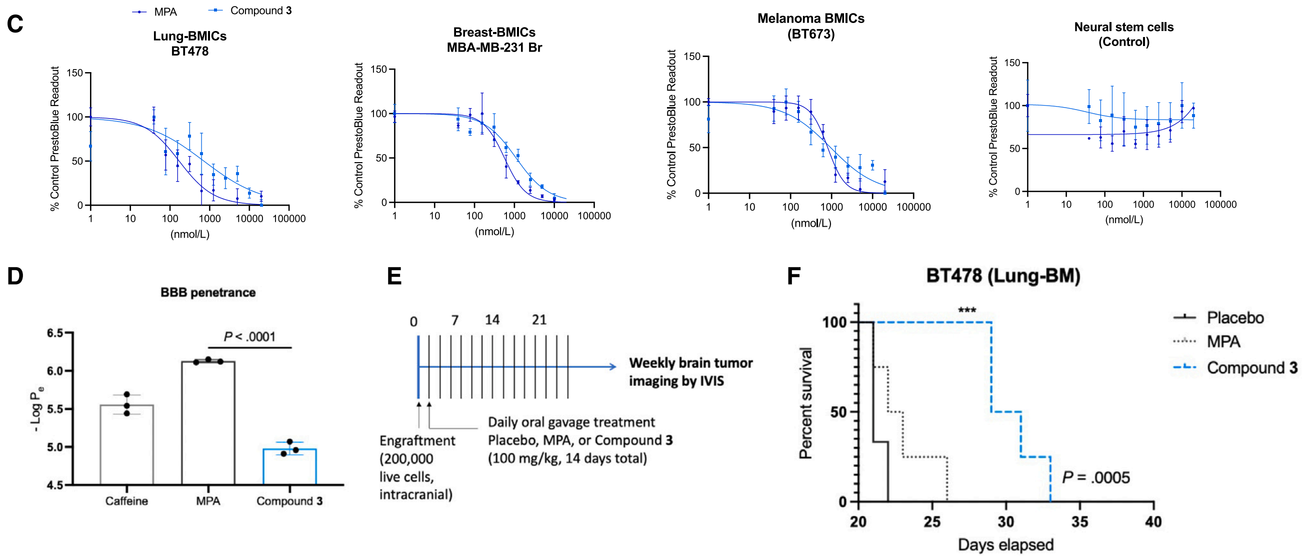

Figure 4. Increasing MPA's brain penetrance enhances its anti-BM phenotype *in vivo*

(A) Summary of the design and synthesis strategy for MPA analogs. See supplementary information for chemical syntheses.

(B) A summary of each analog's activity following dose-response assays. N/A, not applicable.

(C) Dose-response curves of a lung-BMIC line (BT478), breast-BMIC line (MDA-MB-231 Br), melanoma-BMIC line (BT673), and neural stem control cells following a 72-h treatment with MPA or Compound **3**. PrestoBlue readout is normalized to vehicle-treated cells and data are presented as mean ± SD from 4 technical replicates.

(D) Brain penetrance was evaluated *in vitro* using the parallel artificial membrane permeability (PAMPA) assay. Permeability coefficients ($-\text{LogP}_e$) > 6 indicate low CNS permeability, whereas $-\text{LogP}_e$ < 6 indicate high CNS permeability. Caffeine was used as a BBB-permeable control. Comparisons were made via a two-tailed unpaired t test and data are presented as mean ± SD from 3 technical replicates. *p* value is indicated.

(E) Schematic of treatment regimen. Mice were treated daily by oral gavage using either MPA, Compound **3**, or placebo.

(F) *Kaplan-Meier* survival analysis of control-, MPA-, and Compound **3**-treated mice. Log rank test, *p* value is indicated (n.s., not significant [MPA], ***$p = 0.0005$ [Compound **3**]).

equilibrium dialysis and mass spectrometry to determine the fraction of bound (65.9%) and unbound drug (34.1%) for MPA (Table S2). This is a relatively high fraction of unbound drug, consistent with the efficacy of the MPA observed. However, the mass of Compound **3** could not be detected following completion of the assay, and a proper bound/unbound fraction could not be determined. This suggests that Compound **3** is metabolized within the brain homogenate, consistent with our hypothesis of creating a prodrug, and subsequently, likely abides by the bound/unbound ratios determined for MPA.

To examine whether Compound **3**'s suggested BBB permeability leads to superior anti-tumor activity compared to MPA, we intracranially injected mice with lung-BMICs and, 24 h later, began treating the mice daily by oral gavage with either vehicle, MPA, or Compound **3** (Figure 4E). The cells were injected intracranially to ensure that any survival benefit would be due to the compounds crossing the BBB and targeting BMICs in the brain. Mice treated with Compound **3** showed a significant survival advantage in this model compared to both MPA and vehicle-treated groups, whereas there was no survival advantage for MPA-treated mice compared to vehicle (Figure 4F). This suggests that enhancing the BBB permeability of MPA can be done without sacrificing its biological selectivity toward BMICs over normal brain cells and indicates that a compound that

can still reach the tumor cells after brain colonization is critical for longer lasting effects.

Mechanistic studies suggest IMPDH activity as a targetable vulnerability in BMICs

In parallel to our preclinical studies described earlier, we set out to identify whether MPA's known target, IMPDH, is the relevant target in the context of its anticancer mechanism of action. IMPDH is the first rate-limiting enzyme in *de novo* GTP synthesis (Figure 5A). It is an established druggable target known to be upregulated in highly proliferating cells.[38] To confirm whether IMPDH is relevant in MPA's efficacy against BMICs, we initially confirmed that a structurally distinct and selective IMPDH-inhibitor, merimepodib,[39] displays a similar dose-response effect against our patient-derived lung-BMICs (Figure S4A).

Highly proliferative cells rely on *de novo* GTP synthesis to keep up with their high metabolic demands, whereas BMICs, similarly to other cancer stem-like cells, are characterized by a slower rate of proliferation.[40] Therefore, to understand why slowly proliferating BMICs are vulnerable to perturbations in *de novo* GTP synthesis, we sought to elucidate their metabolic landscape. We used liquid chromatography-mass spectrometry-based metabolomics profiling to map the differential polar metabolome of vehicle- or MPA or Compound **3**-treated BM cells in comparison to normal human astrocytes (Figure S4B). Consistent with on-target IMPDH inhibition, GDP and GTP levels were significantly reduced with both MPA and Compound **3** treatment, (Figure 5B),[41] while aminoimidazole carboxamide ribonucleotide (AICAR), an intermediate in the *de novo* purine synthesis pathway upstream of IMPDH, was shown to accumulate with drug treatment (Figure 5C). This confirms that Compound **3** acts on-target and has the same metabolic profile as MPA for both cell lines tested and further suggests that BMICs cannot rely on the salvage pathway (see Figure 5A) to sufficiently fulfill their GTP pools. Notably, MPA and Compound **3** treatment did not influence the levels of dihydroorotic acid (Figure S4C), a key metabolite in pyrimidine biosynthesis, suggesting that the drug's effect is specific to the purine biosynthesis pathway. Taken together, we have shown that *de novo* GTP synthesis is a critical vulnerability in metastatic brain tumors and that IMPDH is a tractable target within this pathway.

We reasoned that if IMPDH is indeed the target of MPA in this biological context, then exogenous guanine supplementation (i.e., upregulating the nucleotide salvage GTP synthesis pathway, which IMPDH is not involved in, see Figure 5A) should fully rescue MPA and Compound **3**'s anti-BMIC phenotype by stimulating the purine nucleotide salvage pathway. As expected, exogenous guanine supplementation to cell culture media (12 μM) rescued BMIC viability after chemical IMPDH perturbation with both MPA and Compound **3** (Figure 5D). This suggests that BMICs may be reliant on *de novo* GTP synthesis, potentially due to a purine salvage deficiency and/or high glucose availability in the brain (see Figure 5A).[42]

To confirm on-target activity of MPA and Compound **3** against IMPDH, we used two patient-derived lung-BMIC cell lines, BT478 and BT530, to generate *IMPDH* knockout lines, wherein both isoenzymes (IMPDH1 and IMPDH2) are knocked out. In parallel, we knocked out the safe-harbor locus, *AAVS1*, as a control cell line. Knockouts were validated by western blot (Figures 5E and S4D). Loss of *IMPDH* led to tumor cell death within 1 week of knockout (Figure 5F). *IMPDH* knockout cell lines exhibited a significantly reduced sphere formation capacity compared to *AAVS1* control cell lines within 72 h (Figure 5G). Furthermore, both MPA and Compound **3** lose their anti-BMIC activity following *IMPDH* knockout (Figure S4E). Taken together, these data provide direct mechanistic evidence that IMPDH is the target responsible for MPA and Compound **3**'s anti-tumor phenotypes and that it is an important driver of BMIC proliferation.

IMPDH expression as a biomarker for patients with lung cancer with BMs

IMPDH is an established druggable target with two isoforms, IMPDH1 and 2, that share 84% peptide sequence homology.[38] *IMPDH1* is generally considered a housekeeping gene, whereas *IMPDH2* is known to be upregulated in highly proliferating cells. Notably, BMICs generally have higher protein levels of both isoenzymes compared to primary lung cancer cells and healthy brain cell controls, as determined by western blot (Figures S4F and S4G). Notably, this difference is only significant for the IMPDH2 isoenzyme.

To investigate whether *IMPDH1* and/or *IMPDH2* could be used as predictive biomarkers for drug response in patients with primary lung cancer (i.e., the primary cancer that accounts for ∼50% of BM[15]), we examined the correlation of *IMPDH1* and *IMPDH2* gene expression with drug sensitivity to MPA across cancer cell lines available in DepMap[43] (Figures S5A and S5F). We found increased MPA drug sensitivity with higher expression of *IMPDH1* in lung cancer cell lines (Pearson correlation coefficient $r = -0.314$, $p = 0.01$). The negative correlation became stronger when subgrouping the data by lung cancer disease subtypes, particularly for non-small cell lung cancer (NSCLC) ($r = -0.884$, $p = 0.116$) and small-cell lung cancer ($r = -0.752$, $p = 0.458$), the lung disease subtypes that most commonly metastasize to the brain.[44] The latter was not statistically significant, likely due to the reduced number of samples in each subgroup.

We next explored the lung tissue expression of *IMPDH1* and *IMPDH2* among normal ($n = 391$), tumor ($n = 1,865$), and metastatic ($n = 8$) tissue samples from data available within TNMplot.[45] We identified that the expression of both genes was increased in the tumor and metastatic tissue compared to normal tissue, with metastatic tissue showing the highest levels of expression (Figures S5G and S5H). Statistically significant differences were obtained from non-parametric Kruskal-Wallis and Dunn test statistical analysis from TNMplot.

We then focused on a study using patients with NSCLC ($n = 30$) who developed BM (available data GSE200563)[46] because it is the most common type of lung cancer, and the patient-derived lung-BM lines used in this work (BT478 and BT530) are of this subtype. We observed that the expression of *IMPDH1* and *IMPDH2* did not correlate within patient samples and between tumor site (Figure S5I). The expression between primary lung carcinoma and its BM-paired sample ($n = 23$) varied (decreased, maintained, or increased) depending on the patient (paired t test $p = 0.317$ and 0.447, respectively, for *IMPDH1* and

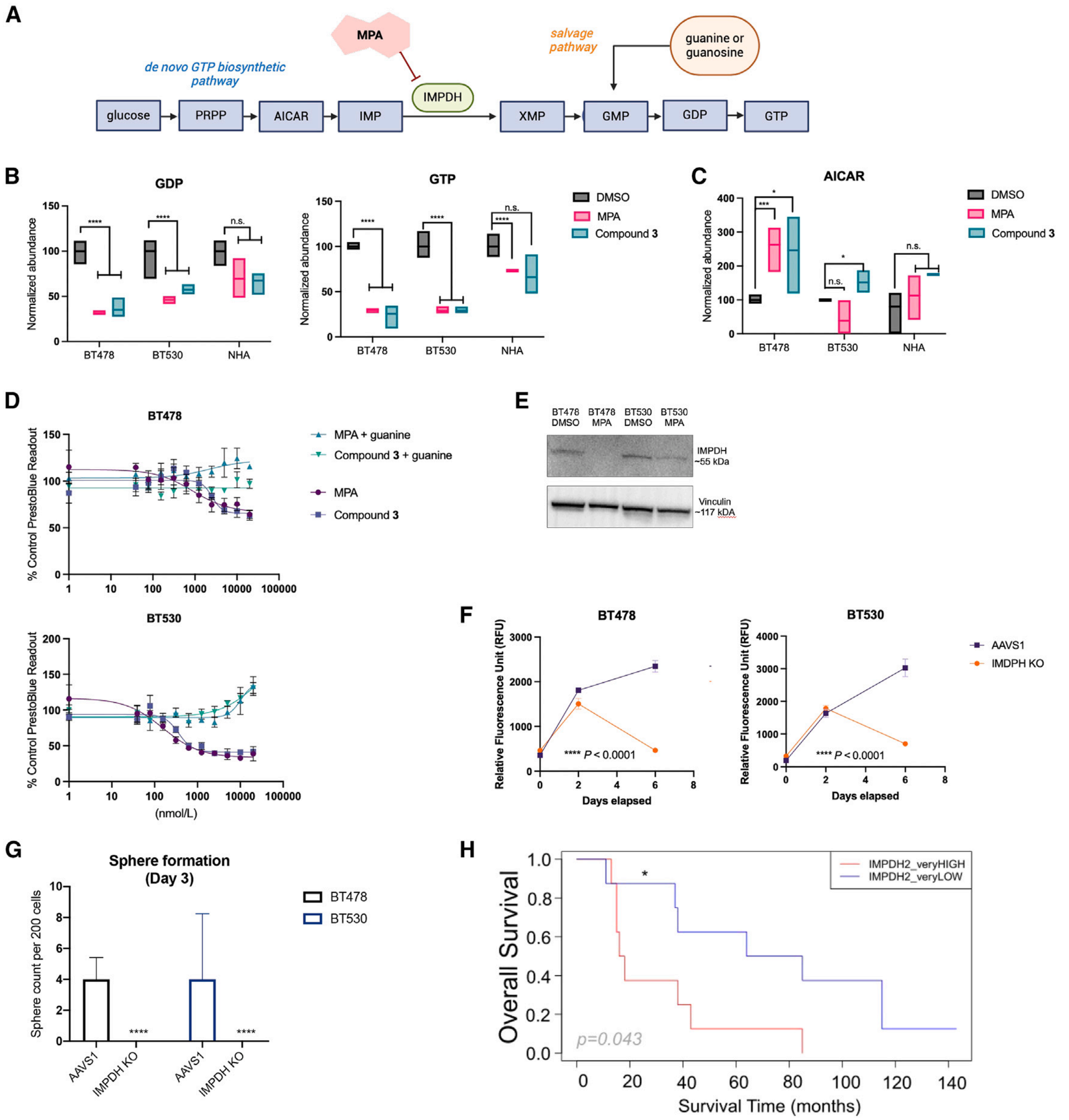

Figure 5. MPA targets the *de novo* GTP synthesis pathway in BMICs

(A) Schematic of the *de novo* GTP synthesis pathway. Created with BioRender.com.

(B) Boxplots depicting relative GDP and GTP levels in patient-derived BMIC lines and normal human astrocytes (NHAs) with either DMSO, MPA, or Compound **3** treatment. Comparisons were made via a two-tailed unpaired t test, $n = 4$ technical replicates, p value is indicated. N.s., not significant, **** = $p < 0.0001$.

(C) Boxplots depicting relative AICAR levels in patient-derived BMIC lines and NHAs with either DMSO, MPA, or Compound **3** treatment. Comparisons were made via a two-tailed unpaired t test, $n = 4$ technical replicates. N.s., not significant.

(D) Dose-response curves of BT478 and BT530 tumor cells treated with MPA or Compound **3** in culture media supplemented with exogenous guanine (12 μM) or vehicle (water). Data are presented as mean ± SD from 4 technical replicates.

(E) Immunoblot confirmation of IMPDH (*IMPDH1* + *IMPDH2*) knockout (KO) in patient-derived lung-BMICs. Cropped from single full blot.

(F) Time-course proliferation assays of AAVS1 and IMPDH KO BT478 and BT530 cells. Comparisons were made via a two-tailed unpaired t test. Data are presented as mean ± SD from 4 technical replicates, **** = $p < 0.0001$.

(G) Representative bar graphs depicting the sphere count per 200 cells of AAVS1 vs. IMPDH KO cells. Comparisons were made via a two-tailed unpaired t test, data are presented as mean ± SD from 4 technical replicates, **** = $p < 0.0001$.

(H) Kaplan-Meier curves showing the overall survival probability (from primary cancer diagnosis to death) in a cohort of 30 patients with lung carcinoma who developed BM, for IMPDH2 very high vs. very low expression (1st and 3rd quartiles cutoff values: <114.86 and >210.74). The Cox regression model for survival analysis was used, p values (p) are shown. The hazard ratio (HR) and 95% confidence interval (CI) are HR = 0.30, CI (0.09–0.96) for IMPDH2 very low compared with very high, or inverted HR = 3.33, CI (1.041–11.11). For the two patients from the cohort who were still alive at the end of the study, the latest survival time point was used for the analysis. Data were obtained from the processed (Q3 method normalization) GEO dataset (GSE200563). Data acquisition, analysis, and visualization performed using R version 4.1.2 and the following packages: GEOquery, survival, and ggplot2.

IMPDH2) (Figures S5J and S5K). No correlation was identified between *IMPDH1* and *IMPDH2* expression in the primary lung carcinoma samples (n = 30) (Pearson r = 0.02, p = 0.10), and a tendency toward a negative correlation for BM (n = 27) was observed, although it was not significant (r = −0.29, p = 0.17) (Figures S5L and S5M).

Finally, the overall survival probability was calculated for the 30 patients with primary lung carcinoma who developed BM using *IMPDH1* and *IMPDH2* expression in the lung cancer tissue (high vs. low expression median cutoff values were 144.72 and 162.75, respectively), and for *IMPDH1* and *IMPDH2* very high vs. very low expression (1st and 3rd quartiles cutoff values: 116.19 and 185.31, and 114.86 and 210.74, respectively) (Figures S5N–S5P and 5H). Notably, very high levels of *IMPDH2* identified a subgroup of patients with significantly shorter overall survival (Kaplan-Meier and Cox regression model for survival analysis, p = 0.043, *IMPDH2* very low: hazard ratio [HR] = 0.30, confidence interval [CI] [0.09–0.96]; *IMPDH2* very high [inverted]: HR = 3.33, CI [1.041–11.11]) (Figure 5H).

While further studies with patient samples are required, these data suggest that IMPDH could be used as both predictive and prognostic biomarkers for patients with lung cancer; higher expression of *IMPDH1* may predict patients with primary lung cancer who could be more responsive to MPA treatment, whereas *IMPDH2* expression could be a prognostic marker for patients with NSCLCwho are at risk for developing BM by identifying those who have a poorer prognosis (i.e., shorter overall survival).

DISCUSSION

BM remains the most common adult brain tumor and the most understudied due to its dismal prognosis and lack of clinically relevant experimental models.[1,47] Current therapies are mainly palliative, highlighting the urgent need for new therapeutic strategies. To address this unmet clinical need, we employed a phenotypic drug screening strategy to identify tool compounds that can be used to unravel promising targets for BM research. Unlike target-based drug screening, phenotypic drug screening intends to identify compounds capable of evoking a desired pharmacological effect (i.e., a compound that kills BMICs without affecting noncancerous brain cell controls).[48] This mode of drug discovery establishes therapeutic relevance earlier in the drug discovery pipeline and enhances the chances of serendipitous discoveries because it does not require prior knowledge of the mechanism of action; this latter point greatly reduces the otherwise common problem of drug off-target effects, which often occurs with target-based drug screening.[49]

Here, we used MPA as a tool compound to reveal IMPDH as a therapeutically tractable target for BM research. We synthesized an analog of MPA (Compound **3**) that was suggested to have enhanced BBB permeability through *in vitro* studies and found, using an *in vivo* PDX model, that this compound increased survival relative to both placebo and MPA. In this study, we show that IMPDH inhibition in BM acts on-target to inhibit *de novo* GTP biosynthesis leading to purine nucleotide depletion.[41] Complete phenotypic rescue with the addition of guanine supports this mechanism of action.

Altered metabolism is a hallmark of cancer[50] and the level of biology closest to the phenotype,[51] making it an attractive target for regulating cancer cell growth. Stem-like glioma cells have been recently reported to reprogram their metabolism to aid in self-renewal, implicating *de novo* GTP synthesis as a cancer dependency in primary brain tumors.[52] The purine nucleotide GTP can be biosynthesized by the salvage pathway or by the *de novo* pathway in cells.[38] Whereas the salvage pathway uses available purine nucleosides to produce purine mononucleotides, *de novo* biosynthesis is an energy-demanding process that is upregulated in many types of cancers. Further, the normal adult brain has lower demands for GTP synthesis and preferentially favors purine salvage,[53,54] which suggests that the salvage pathway is defective or insufficient in BM, thus creating a vulnerability through targeting the *de novo* pathway, rendering IMPDH inhibition as a tractable and nontoxic target for BM with a potentially high therapeutic window.

Purines are the building blocks of DNA and are involved in many cellular processes. This work adds to the growing body of literature implicating MPA as an anti-tumor drug through the suppression of *de novo* purine synthesis,[55] which has also been shown to contribute to the aggressive nature of the primary brain tumors.[41,51] Elevated rates of *de novo* purine synthesis have been shown to maintain the tumorigenic capacity of glioma-initiating cells[56] and contribute to enhanced DNA repair in radiation-resistant glioblastoma.[41,51] We saw a similar dependency for GTP in our study, showing that targeting IMPDH could be beneficial for metastatic brain tumors. Follow-up work should couple these experiments with 13C-glucose stable isotope tracing and flux measurements and further delineate BMIC dependency on GTP synthesis in the context of metastasis.

In other cancers, MPA treatment and subsequent IMPDH inhibition has been shown to interfere with various steps of the cell cycle, which ultimately suppresses cell proliferation.[57] In some cancers, MPA induces differentiation and senescence, with evidence of interfering with cell binding to human umbilical vein endothelial cells, migration into an endothelial cell monolayer, and decreased angiogenesis in the context of vasculitis,[58] which could also be the mechanism behind its blocking of metastasis. Future studies into how IMPDH contributes to not only tumor cell proliferation but also malignant cell invasion and metastasis should be explored.

Clinically, MPA is a Food and Drug Administration (FDA)-approved drug used for the prophylaxis of organ rejection in transplant patients by targeting the increased IMPDH levels in upregulated T cells and B cells following immune response.[38,59] IMPDH activity and *de novo* GTP synthesis is not an inherent mechanism of immune cells, and patients with cancer are typically already taking immunosuppressants during their treatment. Nonetheless, a limitation of our PDX mouse models is the lack of an intact immune system and this should be the subject of future follow-up studies, along with testing the effects of IMPDH inhibitors on established BM tumors and those of varying primary tumors, particularly those of breast and melanoma origin. Notably, other groups have shown that IMPDH inhibition does not have a devastating effect on immune function *in vivo* when used as a cancer treatment,[60] and current clinical trials using MPA

prodrugs are already underway for glioblastoma with no serious toxicities (NCT04477200).[61]

Collectively, our studies implicate *de novo* purine synthesis as a metabolic vulnerability in BM that is targetable through its rate-limiting enzyme, IMPDH. IMPDH inhibitors are already an FDA-approved class of drugs used clinically, making the barrier to clinical translation low,[41,56] which is particularly important for this patient population, whose median survival remains at 4–12 months. Here, we have shown in a proof- of- concept that the BBB-permeable Compound **3** has improved preclinical activity compared to MPA, indicating that an IMPDH inhibitor that can still reach the tumor cells after brain colonization is critical for longer lasting effects. These data, paired with the notion that IMPDH inhibitors are selective toward BMICs but seemingly nontoxic to normal brain tissue, have warranted further research into the metabolic profiles of BMICs and the optimization of more drug-like brain-penetrant IMPDH inhibitors for clinical translation. To facilitate identification of patients at a high risk of developing BM following a primary tumor diagnosis, more focus should be directed on whether IMPDH expression in primary tissues can serve as a predictive biomarker for IMPDH inhibition efficacy on BM, and whether high IMPDH expression can be used as a prognostic biomarker, correlated with bad prognosis. If effective, targeting *de novo* GTP synthesis in BMICs could slow their metastatic ability and serve as a first-in-class anti-BM therapy, while translating to the future development of other anticancer therapies for tumors with the same metabolic dependencies. Overall, the work described here serves to progress BM research toward the goal of improving the current palliative standard of care for patients with BM with a targeted therapy aimed to eliminate BM.

Limitations of the study

In our orthotopic PDX mouse models, we have recapitulated BM formation by orthotopically implanting patient-derived metastatic lung cancer cells into the thoracic cavity. In our drug dosing experiments, we included models where we pre-treated BMICs with MPA prior to engraftment or dosed the mice *in vivo* following injection. The pre-treatment model was necessary because our tool compound, MPA, is predicted to have poor BBB permeability. Our results suggest that metastasis is intercepted in the pre-treatment model, but not in the *in vivo* treatment model, suggesting that BMICs are not being effectively targeted by MPA once they cross the BBB to seed the brain. It could, however, be hypothesized that MPA does not affect the number of BMICs residing in the primary tumor, but rather intercepts their ability to effectively seed the brain, perhaps because slowly proliferating cancer stem cells are less susceptible to the drug than highly proliferative, non-stem tumor cells. Hence, future medicinal chemistry work to develop a brain-potent IMPDH inhibitor should be followed up with these same models, to further elucidate these findings. Altogether, this highlights the importance of IMPDH in the metastatic process for future work. Further, while our metabolomics data suggest that GTP synthesis is blocked with MPA and Compound **3** through a decrease of both GDP and GTP and that IMPDH inhibition is a consistent vulnerability in all BMIC lines tested, the upstream metabolite AICAR accumulates in only some conditions; future studies using isotope tracing would be important to confirm the importance of *de novo* GTP synthesis specifically. Lastly, while our *in silico*, PAMPA, and MDCK-MDR1 cell monolayer assays suggest that Compound **3** has greater BBB permeability compared to MPA, complete *in vivo* pharmacokinetic studies would be necessary to confidently demonstrate that this is the case.

RESOURCE AVAILABILITY

Lead contact

Further information and detail requests regarding this paper can be directed to and will be fulfilled by the lead contact, Dr. Sheila Singh (ssingh@mcmaster.ca).

Materials availability

There are restrictions to the availability of the compounds and uses thereof for brain metastases due to patents.

Data and code availability

- This paper analyzes existing, publicly available data. These accession numbers for the datasets are listed in the key resources table.
- Metabolomics mass spectrometry raw data that support the findings of this study have been deposited in FigShare and are publicly available as the date of publication. Accession numbers are listed in the key resources table.
- This paper does not report original code.
- Any additional information required to reanalyze the data reported in this paper is available from the lead contact upon request.

ACKNOWLEDGMENTS

This study was funded by Canadian Cancer Society Research Institute (S.K.S.), Donations from Boris Family (J.M. and S.K.S.), Donations from McMaster University Department of Surgery (S.K.S.), Ontario Institute of Cancer Research (S.K.S.), Canada Foundation for Innovation (J.R.M.-B.), Natural Sciences and Engineering Research Council of Canada (J.R.M.-B.), Ontario Graduate Fellowship (A.M.K.), MITACS fellowship (A.M.K.), and Brain Tumor Foundation of Canada Summer Scholarship (Taite Boomer Foundation, D.Mobilio). S.K.S holds a Senior Canadian Research Chair in Human Cancer Stem Cell Biology at McMaster University.

AUTHOR CONTRIBUTIONS

Conceptualization, A.M.K., C.V., and S.K.S.; methodology, A.M.K., B.I.B.-A., J.W.J., W.D.G., C.V., J.M., and S.K.S.; investigation, A.M.K., D. Mobilio, B.I.B.-A., J.W.J., M.L.P., N.A., C.Z., A.Q., M.S., E.D.d.A., A.S., and P.A.; visualization, A.M.K., D. Mobilio, L.E., J.W.J., C.Z., E.D.d.A., T.T., and S.K.S.; writing – original draft, A.M.K., J.W.J., and L.E.; writing – review and editing, A.M.K., J.W.J., L.E., S.C., K.Z., C.V., and S.K.S.; project administration, D. McKenna, M.S., P.T.G., J.R.M.-B., C.V., J.M., and S.K.S.; supervision, C.V. and S.K.S.

DECLARATION OF INTERESTS

A.M.K., J.W.J., C.V., J.M., and S.K.S. are listed as co-inventors for a PCT patent that has been filed, relating to this work.

STAR★METHODS

Detailed methods are provided in the online version of this paper and include the following:

- KEY RESOURCES TABLE
- EXPERIMENTAL MODEL AND STUDY PARTICIPANT DETAILS
  - Cell culture
  - *In vivo* preclinical studies

- METHOD DETAILS
  - Connectivity Map analysis
  - *In vitro* functional assays
  - Clonogenic sphere formation assays
  - Limiting dilution assays
  - Migration assay
  - Compounds
  - PAMPA assay
  - MDR1-MDCKII assays
  - Brain tissue binding assay
  - Western Blot
  - Firefly-luciferase lentivirus generation
  - *In vivo* imaging
  - Fluorescence-activated cell sorting
  - Metabolomics mass spectrometry
  - Generation of IMPDH knockout lines
- QUANTIFICATION AND STATISTICAL ANALYSIS
  - Statistical analysis

SUPPLEMENTAL INFORMATION

Supplemental information can be found online at https://doi.org/10.1016/j.xcrm.2024.101755.

Received: December 11, 2023
Revised: May 21, 2024
Accepted: September 6, 2024
Published: October 1, 2024

REFERENCES

1. Patchell, R.A. (2003). The management of brain metastases. Cancer Treat Rev. *29*, 533–540. https://doi.org/10.1016/S0305-7372(03)00105-1.
2. Soffietti, R., Cornu, P., and Delattre, J.Y. (2011). Brain metastases 10.1038/s41572-018-0055-y.
3. Liu, Q., Tong, X., and Wang, J. (2019). Management of brain metastases: history and the present. Chin. Neurosurg. J. *5*, 1. https://doi.org/10.1186/s41016-018-0149-0.
4. Han, C.H., and Brastianos, P.K. (2017). Genetic characterization of brain metastases in the era of targeted therapy. Front. Oncol. *7*, 230. https://doi.org/10.3389/fonc.2017.00230.
5. PK, B., SL, C., and S, S. (2015). Genomic characterization of brain metastases reveals branched evolution and potential therapeutic targets. Cancer Discov. *5*, 1164–1177.
6. Shih, D.J.H., Nayyar, N., Bihun, I., Dagogo-Jack, I., Gill, C.M., Aquilanti, E., Bertalan, M., Kaplan, A., D'Andrea, M.R., Chukwueke, U., et al. (2020). Genomic characterization of human brain metastases identifies drivers of metastatic lung adenocarcinoma. Nat. Genet. *52*, 371–377. https://doi.org/10.1038/s41588-020-0592-7.
7. Luzzi, K.J., MacDonald, I.C., Schmidt, E.E., Kerkvliet, N., Morris, V.L., Chambers, A.F., and Groom, A.C. (1998). Multistep Nature of Metastatic Inefficiency: Dormancy of Solitary Cells after Successful Extravasation and Limited Survival of Early Micrometastases. Am. J. Pathol. *153*, 865–873. https://doi.org/10.1016/S0002-9440(10)65628-3.
8. Ayob, A.Z., and Ramasamy, T.S. (2018). Cancer stem cells as key drivers of tumour progression. J. Biomed. Sci. *25*, 20. https://doi.org/10.1186/s12929-018-0426-4.
9. Hermann, P.C., Huber, S.L., and Heeschen, C. (2008). Cell Cycle Metastatic cancer stem cells: A new target for anti-cancer therapy? Cell Cycle *7*, 188–193. https://doi.org/10.4161/cc.7.2.5326.
10. Baccelli, I., and Trumpp, A. (2012). The evolving concept of cancer and metastasis stem cells. J. Cell Biol. *198*, 281–293. https://doi.org/10.1083/jcb.201202014.
11. Pang, A.W., MacDonald, J.R., Pinto, D., Wei, J., Rafiq, M.A., Conrad, D.F., Park, H., Hurles, M.E., Lee, C., Venter, J.C., et al. (2010). Towards a comprehensive structural variation map of an individual human genome. Genome Biol. *11*, R52. https://doi.org/10.1186/gb-2010-11-5-r52.
12. Singh, M., Venugopal, C., Tokar, T., Brown, K.R., McFarlane, N., Bakhshinyan, D., Vijayakumar, T., Manoranjan, B., Mahendram, S., Vora, P., et al. (2017). RNAi screen identifies essential regulators of human brain metastasis-initiating cells. Acta Neuropathol. *134*, 923–940. https://doi.org/10.1007/s00401-017-1757-z.
13. Singh, M., Venugopal, C., Tokar, T., Mcfarlane, N., Subapanditha, M.K., Qazi, M., Bakhshinyan, D., Vora, P., Murty, N.K., Jurisica, I., and Singh, S.K. (2018). Therapeutic Targeting of the Premetastatic Stage in Human Lung-to-Brain Metastasis. Cancer Res. *78*, 5124–5134. https://doi.org/10.1158/0008-5472.CAN-18-1022.
14. Bassey-Archibong, B., Chokshi, C., Aghaei, N., Kieliszek, A., Tatari, N., McKenna, D., Singh, M., Subapanditha, M., Parmar, A., Mobilio, D., et al. (2023). An HLA-G/SPAG9/STAT3 axis promotes brain metastases. Proc. Natl. Acad. Sci. USA *120*, e2205247120.
15. Singh, M., Manoranjan, B., Mahendram, S., McFarlane, N., Venugopal, C., Singh, S.K., Singh, M., Manoranjan, B., Mahendram, S., McFarlane, N., et al. (2014). Brain metastasis-initiating cells: survival of the fittest. Int. J. Mol. Sci. *15*, 9117–9133. https://doi.org/10.3390/ijms15059117.
16. Kieliszek, A.M., Aghaei, N., Bassey-Archibong, B.I., and Singh, S.K. (2022). Low and steady wins the race: For melanoma brain metastases, is prevention better than a cure? Neuro Oncol. *24*, 226–228. https://doi.org/10.1093/neuonc/noab267.
17. Lamb, J., Crawford, E.D., Peck, D., Modell, J.W., Blat, I.C., Wrobel, M.J., Lerner, J., Brunet, J.-P., Subramanian, A., Ross, K.N., et al. (2006). The Connectivity Map: Using Gene-Expression Signatures to Connect Small Molecules, Genes, and Disease. Science *313*, 1929–1935. https://doi.org/10.1126/science.1132939.
18. Singh, M., Savage, N., and Singh, S.K. (2019). Vivo Murine Models of Brain Metastasis. Methods Mol. Biol. *1869*, 231–238. https://doi.org/10.1007/978-1-4939-8805-1_19.
19. Xiong, B., Wang, Y., Chen, Y., Xing, S., Liao, Q., Chen, Y., Li, Q., Li, W., and Sun, H. (2021). Strategies for Structural Modification of Small Molecules to Improve Blood–Brain Barrier Penetration: A Recent Perspective. J. Med. Chem. *64*, 13152–13173. https://doi.org/10.1021/acs.jmedchem.1c00910.
20. Pajouhesh, H., and Lenz, G.R. (2005). Medicinal chemical properties of successful central nervous system drugs. NeuroRx *2*, 541–553. https://doi.org/10.1602/neurorx.2.4.541.
21. Neumann, I., Fuhrmann, H., Fang, I.-F., Jaeger, A., Bayer, P., and Kovarik, J. (2008). Association between mycophenolic acid 12-h trough levels and clinical endpoints in patients with autoimmune disease on mycophenolate mofetil. Nephrol. Dial. Transplant. *23*, 3514–3520. https://doi.org/10.1093/ndt/gfn360.
22. Nolte, S.M., Venugopal, C., McFarlane, N., Morozova, O., Hallett, R.M., O'Farrell, E., Manoranjan, B., Murty, N.K., Klurfan, P., Kachur, E., et al. (2013). A Cancer Stem Cell Model for Studying Brain Metastases From Primary Lung Cancer. J. Natl. Cancer Inst. *105*, 551–562. https://doi.org/10.1093/jnci/djt022.
23. Hu, Y., and Smyth, G.K. (2009). ELDA: extreme limiting dilution analysis for comparing depleted and enriched populations in stem cell and other assays. J. Immunol. Methods *347*, 70–78. https://doi.org/10.1016/j.jim.2009.06.008.
24. Lopez, T., Wendremaire, M., Lagarde, J., Duquet, O., Alibert, L., Paquette, B., Garrido, C., and Lirussi, F. (2022). Wound Healing versus Metastasis: Role of Oxidative Stress. Biomedicines *10*, 2784. https://doi.org/10.3390/biomedicines10112784.
25. Flier, J.S., Underhill, L.H., and Dvorak, H.F. (1986). Tumors: Wounds That Do Not Heal. N. Engl. J. Med. *315*, 1650–1659. https://doi.org/10.1056/NEJM198612253152606.

CellPress
OPEN ACCESS

26. Kondratyev, M., Kreso, A., Hallett, R.M., Girgis-Gabardo, A., Barcelon, M.E., Ilieva, D., Ware, C., Majumder, P.K., and Hassell, J.A. (2012). Gamma-secretase inhibitors target tumor-initiating cells in a mouse model of ERBB2 breast cancer. Oncogene *31*, 93–103. https://doi.org/10.1038/onc.2011.212.

27. Gwynne, W.D., Shakeel, M.S., Girgis-Gabardo, A., Kim, K.H., Ford, E., Dvorkin-Gheva, A., Aarts, C., Isaac, M., Al-awar, R., and Hassell, J.A. (2020). Antagonists of the serotonin receptor 5A target human breast tumor initiating cells. BMC Cancer *20*, 724. https://doi.org/10.1186/s12885-020-07193-6.

28. Rautio, J., Kumpulainen, H., Heimbach, T., Oliyai, R., Oh, D., Järvinen, T., and Savolainen, J. (2008). Prodrugs: design and clinical applications. Nat. Rev. Drug Discov. *7*, 255–270. https://doi.org/10.1038/nrd2468.

29. Morris, R.E., Hoyt, E.G., Murphy, M.P., Eugui, E.M., and Allison, A.C. (1990). Mycophenolic acid morpholinoethyl ester (RS-61443) is a new immunosuppressant that prevents and halts heart allograft rejection by selective inhibition of T-and B-cell purine synthesis. Transplant. Proc. *22*, 1659–1662.

30. Nelson, P.H., Gu, C.L.L., Allison, A.C., Eugui, E.M., and Lee, W.A. (1988). Preparation of morpholinoethyl esters of mycophenolic acid and pharmaceutical compositions containing them as immunosuppressive and anti-inflammatory agents.

31. Lipsky, J.J. (1996). Mycophenolate mofetil. Lancet *348*, 1357–1359. https://doi.org/10.1016/S0140-6736(96)10310-X.

32. Lee, S., Ku, A.F., Vippila, M.R., Wang, Y., Zhang, M., Wang, X., Hedstrom, L., and Cuny, G.D. (2020). Mycophenolic anilides as broad specificity inosine-5'-monophosphate dehydrogenase (IMPDH) inhibitors. Bioorg. Med. Chem. Lett. *30*, 127543. https://doi.org/10.1016/j.bmcl.2020.127543.

33. Shah, C.P., and Kharkar, P.S. (2018). Newer human inosine 5'-monophosphate dehydrogenase 2 (hIMPDH2) inhibitors as potential anticancer agents. J. Enzym. Inhib. Med. Chem. *33*, 972–977. https://doi.org/10.1080/14756366.2018.1474211.

34. Kerns, E. (2009). Comparison of blood-brain barrier permeability assays: in situ brain perfusion, MDR1-MDCKII and PAMPA-BBB. J. Pharmaceut. Sci. *98*, 1980–1991.

35. Nawar, N., Bukhari, S., Adile, A.A., Suk, Y., Manaswiyoungkul, P., Toutah, K., Olaoye, O.O., Raouf, Y.S., Sedighi, A., Garcha, H.K., et al. (2022). Discovery of HDAC6-Selective Inhibitor NN-390 with *in Vitro* Efficacy in Group 3 Medulloblastoma. J. Med. Chem. *65*, 3193–3217. https://doi.org/10.1021/acs.jmedchem.1c01585.

36. Wang, Q., Rager, J.D., Weinstein, K., Kardos, P.S., Dobson, G.L., Li, J., and Hidalgo, I.J. (2005). Evaluation of the MDR-MDCK cell line as a permeability screen for the blood-brain barrier. Int. J. Pharm. *288*, 349–359. https://doi.org/10.1016/j.ijpharm.2004.10.007.

37. Lee, G., Schlichter, L., Bendayan, M., and Bendayan, R. (2001). Functional Expression of P-glycoprotein in Rat Brain Microglia. J. Pharmacol. Exp. Therapeut. *299*, 204–212.

38. Naffouje, R., Grover, P., Yu, H., Sendilnathan, A., Wolfe, K., Majd, N., Smith, E.P., Takeuchi, K., Senda, T., Kofuji, S., and Sasaki, A.T. (2019). Anti-Tumor Potential of IMP Dehydrogenase Inhibitors: A Century-Long Story. Cancers *11*, 1346. https://doi.org/10.3390/cancers11091346.

39. Looker, A.R., Littler, B.J., Blythe, T.A., Snoonian, J.R., Ansell, G.K., Jones, A.D., Nyce, P., Chen, M., and Neubert, B.J. (2008). Development and Manufacture of the Inosine Monophosphate Dehydrogenase Inhibitor Merimepodib, VX-497. Org. Process Res. Dev. *12*, 666–673. https://doi.org/10.1021/op800060h.

40. Moore, N., and Lyle, S. (2011). Quiescent, slow-cycling stem cell populations in cancer: a review of the evidence and discussion of significance. JAMA Oncol. *2011*, 396076. https://doi.org/10.1155/2011/396076.

41. Zhou, W., Yao, Y., Scott, A.J., Wilder-Romans, K., Dresser, J.J., Werner, C.K., Sun, H., Pratt, D., Sajjakulnukit, P., Zhao, S.G., et al. (2020). Purine metabolism regulates DNA repair and therapy resistance in glioblastoma. Nat. Commun. *11*, 3811. https://doi.org/10.1038/s41467-020-17512-x.

42. Dienel, G.A. (2019). Brain Glucose Metabolism: Integration of Energetics with Function. Physiol. Rev. *99*, 949–1045. https://doi.org/10.1152/physrev.00062.2017.

43. Corsello, S.M., Nagari, R.T., Spangler, R.D., Rossen, J., Kocak, M., Bryan, J.G., Humeidi, R., Peck, D., Wu, X., Tang, A.A., et al. (2020). Discovering the anti-cancer potential of non-oncology drugs by systematic viability profiling. Nat. cancer *1*, 235–248. https://doi.org/10.1038/s43018-019-0018-6.

44. Müller, S.J., Khadhraoui, E., Neef, N.E., Riedel, C.H., and Ernst, M. (2021). Differentiation of brain metastases from small and non-small lung cancers using apparent diffusion coefficient (ADC) maps. BMC Med. Imag. *21*, 70. https://doi.org/10.1186/s12880-021-00602-7.

45. Bartha, Á., and Győrffy, B. (2021). TNMplot.com: A Web Tool for the Comparison of Gene Expression in Normal, Tumor and Metastatic Tissues. Int. J. Mol. Sci. *22*, 2622. https://doi.org/10.3390/ijms22052622.

46. Zhang, Q., Abdo, R., Iosef, C., Kaneko, T., Cecchini, M., Han, V.K., and Li, S.S.-C. (2022). The spatial transcriptomic landscape of non-small cell lung cancer brain metastasis. Nat. Commun. *13*, 5983. https://doi.org/10.1038/s41467-022-33365-y.

47. Gupta, G.P., and Massagué, J. (2006). Cancer Metastasis: Building a Framework. Cell *127*, 679–695. https://doi.org/10.1016/J.CELL.2006.11.001.

48. Kiriiri, G.K., Njogu, P.M., and Mwangi, A.N. (2020). Exploring different approaches to improve the success of drug discovery and development projects: a review. Futur. J. Pharm. Sci. *6*, 27. https://doi.org/10.1186/s43094-020-00047-9.

49. Lin, A., Giuliano, C.J., Palladino, A., John, K.M., Abramowicz, C., Yuan, M.L., Sausville, E.L., Lukow, D.A., Liu, L., Chait, A.R., et al. (2019). Off-target toxicity is a common mechanism of action of cancer drugs undergoing clinical trials. Sci. Transl. Med. *11*, eaaw8412. https://doi.org/10.1126/scitranslmed.aaw8412.

50. Hanahan, D., and Weinberg, R.A. (2011). Hallmarks of Cancer: The Next Generation. Cell *144*, 646–674. https://doi.org/10.1016/j.cell.2011.02.013.

51. Zhou, W., and Wahl, D.R. (2020). Purine metabolism promotes radioresistance and is a therapeutic target in glioblastoma. Mol. Cell. Oncol. *7*, 1834902. https://doi.org/10.1080/23723556.2020.1834902.

52. Bao, S., Wu, Q., McLendon, R.E., Hao, Y., Shi, Q., Hjelmeland, A.B., Dewhirst, M.W., Bigner, D.D., and Rich, J.N. (2006). Glioma stem cells promote radioresistance by preferential activation of the DNA damage response. Nature *444*, 756–760. https://doi.org/10.1038/nature05236.

53. Wen, P.Y., Lee, E.Q., Reardon, D.A., Ligon, K.L., and Alfred Yung, W.K. (2012). Current clinical development of PI3K pathway inhibitors in glioblastoma. Neuro Oncol. *14*, 819–829. https://doi.org/10.1093/neuonc/nos117.

54. Allsop, J., and Watts, R.W.E. (1984). Purine Synthesis and Salvage in Brain and Liver (Springer), pp. 21–26. https://doi.org/10.1007/978-1-4757-0390-0_5.

55. Hu, J., Locasale, J.W., Bielas, J.H., O'Sullivan, J., Sheahan, K., Cantley, L.C., Vander Heiden, M.G., and Vitkup, D. (2013). Heterogeneity of tumor-induced gene expression changes in the human metabolic network. Nat. Biotechnol. *31*, 522–529. https://doi.org/10.1038/nbt.2530.

56. Wang, X., Yang, K., Xie, Q., Wu, Q., Mack, S.C., Shi, Y., Kim, L.J.Y., Prager, B.C., Flavahan, W.A., Liu, X., et al. (2017). Purine synthesis promotes maintenance of brain tumor initiating cells in glioma. Nat. Neurosci. *20*, 661–673. https://doi.org/10.1038/nn.4537.

57. Benjanuwattra, J., Chaiyawat, P., Pruksakorn, D., and Koonrungsesomboon, N. (2020). Therapeutic potential and molecular mechanisms of mycophenolic acid as an anticancer agent. Eur. J. Pharmacol. *887*, 173580. https://doi.org/10.1016/j.ejphar.2020.173580.

58. Huang, Y., Liu, Z., Huang, H., Liu, H., and Li, L. (2005). Effects of mycophenolic acid on endothelial cells. Int. Immunopharm. *5*, 1029–1039. https://doi.org/10.1016/j.intimp.2005.01.015.

59. Allison, A.C., and Eugui, E.M. (2000). Mycophenolate mofetil and its mechanisms of action. Immunopharmacology *47*, 85–118. https://doi.org/10.1016/s0162-3109(00)00188-0.

60. Liu, X., Sato, N., Yabushita, T., Li, J., Jia, Y., Tamura, M., Asada, S., Fujino, T., Fukushima, T., Yonezawa, T., et al. (2023). IMPDH inhibition activates TLR-VCAM1 pathway and suppresses the development of MLL-fusion leukemia. EMBO Mol. Med. *15*, e15631. https://doi.org/10.15252/emmm.202115631.

61. Umemura, Y., Sun, Y., Junck, L., Leung, D., Kim, M., Al-Holou, W., Sagher, O., Heth, J., Lyssiotis, C., Marini, B., et al. (2021). DDRE-19. PHASE 0/I TRIAL OF MYCOPHENOLATE MOFETIL COMBINED WITH RADIATION TO OVERCOME GLIOBLASTOMA TREATMENT RESISTANCE BY TARGETING DE-NOVO PURINE METABOLISM. Neurooncol. Adv. *3*, i10. https://doi.org/10.1093/noajnl/vdab024.041.

62. Suk, Y., Kieliszek, A., Mobilio, D., Venugopal, C., and Singh, S.K. (2022). Derivation and culturing of neural stem cells from human embryonic brain tissue. STAR Protoc. *3*, 101628. https://doi.org/10.1016/J.XPRO.2022.101628.

63. Singh, M., Bakhshinyan, D., Venugopal, C., and Singh, S.K. (2017). Preclinical Modeling and Therapeutic Avenues for Cancer Metastasis to the Central Nervous System. Front. Oncol. *7*, 220. https://doi.org/10.3389/FONC.2017.00220.

64. Singh, S., and Venugopal, C. (2019). Brain Tumor Stem Cells: Methods and Protocols, 1st ed, S.K. Singh and C. Venugopal, eds. (Humana Press). https://doi.org/10.1007/978-1-4939-8805-1.

65. Singh, M., Garg, N., Venugopal, C., Hallett, R., Tokar, T., McFarlane, N., Mahendram, S., Bakhshinyan, D., Manoranjan, B., Vora, P., et al. (2015). STAT3 pathway regulates lung-derived brain metastasis initiating cell capacity through miR-21 activation. Oncotarget *6*, 27461–27477. https://doi.org/10.18632/oncotarget.4742.

66. Adile, A.A., Bakhshinyan, D., Venugopal, C., and Singh, S.K. (2019). In Vitro Assays for Screening Small Molecules. Methods Mol. Biol. *1869*, 189–196. https://doi.org/10.1007/978-1-4939-8805-1_16.

67. Qazi, M.A., Vora, P., Venugopal, C., Adams, J., Singh, M., Hu, A., Gorelik, M., Subapanditha, M.K., Savage, N., Yang, J., et al. (2018). Cotargeting Ephrin Receptor Tyrosine Kinases A2 and A3 in Cancer Stem Cells Reduces Growth of Recurrent Glioblastoma. Cancer Res. *78*, 5023–5037. https://doi.org/10.1158/0008-5472.CAN-18-0267.

68. Venugopal, C., Hallett, R., Vora, P., Manoranjan, B., Mahendram, S., Qazi, M.A., McFarlane, N., Subapanditha, M., Nolte, S.M., Singh, M., et al. (2015). Pyrvinium Targets CD133 in Human Glioblastoma Brain Tumor-Initiating Cells. Clin. Cancer Res. *21*, 5324–5337. https://doi.org/10.1158/1078-0432.CCR-14-3147.

69. Manoranjan, B., Chokshi, C., Venugopal, C., Subapanditha, M., Savage, N., Tatari, N., Provias, J.P., Murty, N.K., Moffat, J., Doble, B.W., and Singh, S.K. (2020). A CD133-AKT-Wnt signaling axis drives glioblastoma brain tumor-initiating cells. Oncogene *39*, 1590–1599. https://doi.org/10.1038/s41388-019-1086-x.

70. Seyfrid, M., Bobrowski, D., Bakhshinyan, D., Tatari, N., Venugopal, C., and Singh, S.K. (2019). In Vitro Self-Renewal Assays for Brain Tumor Stem Cells (Humana Press), pp. 79–84. https://doi.org/10.1007/978-1-4939-8805-1_7.

71. Gottlieb, H.E., Kotlyar, V., and Nudelman, A. (1997). NMR Chemical Shifts of Common Laboratory Solvents as Trace Impurities. J. Org. Chem. *62*, 7512–7515. https://doi.org/10.1021/JO971176V.

72. Singh, S.K., and Venugopal, C. (2019). In Brain Tumor Stem Cells (Springer). https://doi.org/10.1007/978-1-4939-8805-1.

73. Still, W.C., Kahn, M., and Mitra, A. (1978). Rapid chromatographic technique for preparative separations with moderate resolution. J. Org. Chem. *43*, 2923–2925. https://doi.org/10.1021/jo00408a041.

74. Arndt-Jovin, D.J., and Jovin, T.M. (1989). Fluorescence labeling and microscopy of DNA. Methods Cell Biol. *30*, 417–448.

75. Gwynne, W.D., Suk, Y., Custers, S., Mikolajewicz, N., Chan, J.K., Zador, Z., Chafe, S.C., Zhai, K., Escudero, L., Zhang, C., et al. (2022). Cancer-selective metabolic vulnerabilities in MYC-amplified medulloblastoma. Cancer Cell *40*, 1488–1502.e7. https://doi.org/10.1016/J.CCELL.2022.10.009.

76. Hart, T., Tong, A.H.Y., Chan, K., Van Leeuwen, J., Seetharaman, A., Aregger, M., Chandrashekhar, M., Hustedt, N., Seth, S., Noonan, A., et al. (2017). Evaluation and Design of Genome-Wide CRISPR/SpCas9 Knockout Screens. G3 (Bethesda). *7*, 2719–2727. https://doi.org/10.1534/G3.117.041277.

77. Hart, T., Chandrashekhar, M., Aregger, M., Steinhart, Z., Brown, K.R., MacLeod, G., Mis, M., Zimmermann, M., Fradet-Turcotte, A., Sun, S., et al. (2015). High-Resolution CRISPR Screens Reveal Fitness Genes and Genotype-Specific Cancer Liabilities. Cell *163*, 1515–1526. https://doi.org/10.1016/j.cell.2015.11.015.

STAR★METHODS

KEY RESOURCES TABLE

REAGENT or RESOURCE	SOURCE	IDENTIFIER
Antibodies		
IMPDH Antibody (F-6)	Santa Cruz	Cat#sc-166551; RRID:AB_2127354
mouse anti-GAPDH	Abcam	Cat#ab8245; RRID:AB_2107448
APC-conjugated anti-human TRA-1-85 (CD147)	Miltenyi Biotec	Cat#130-128-900; RRID:AB_2921968
HRP conjugated secondary anti mouse	BioRad	Cat#1706516; RRID:AB_11125547
Bacterial and virus strains		
NEB Stable Competent E. coli	NEB	C3040
Biological samples		
MDA-MB-231	ATCC	Cat#HTB-26
Normal human astrocytes	Lonza Bioscience	Discontinued
HEK293T	ATCC	Cat#CRL-11268
Chemicals, peptides, and recombinant proteins		
Mycophenlic acid	Tocris	Cat#1505
PrestoBlue Cell Viability Reagent	ThermoFisher Scientific	Cat#A13261
SYTOX^{TM} Green Nucleic Acid Stain	Invitrogen	Cat#S7020
Incucyte® Nuclight Rapid Red Dye for Live-Cell Nuclear Labeling	Sartorius	Cat#4717
7AAD Viability dye	Beckman Coulter	Cat#A07704
Liberase Blendzyme 3	Millipore Sigma	Cat#5401119001
Heparin Solution		Cat#07980
Human Recombinant Basic Fibroblast Growth Factor (bFGF)	STEMCELL Technologies	Cat#78003
Human Recombinant Epidermal Growth Factor (EGF)	STEMCELL Technologies	Cat#78006
Antibiotic-Antimycotic (100X)	Wisent Bioproducts	Cat#450-115-EL
Lipofectamine 3000	ThermoFisher	Cat#L3000075
Fetal Bovine Serum, heat inactivated (FBS)	Wisent	Cat#098-150
PBS	ThermoFisher	Cat#10010049
DMEM	ThermoFisher	Cat#11995073
EDTA	Millipore Sigma	Cat#20158
Guanine	Sigma-Aldrich	Cat#G11950
Paraformaldehyde	Electron Microscopy Biosciences	Cat#RT15700
D-firefly luciferin potassium salt	Perkin Elmer	Cat#122799
Critical commercial assays		
Bradford Assay	BioRad	Cat#5000112
Deposited data		
Lung-brain metastasis RNA seq data	Gene Expression Omnibus	GSE110495
Breast- and melanoma-brain metastasis RNA seq data	Gene Expression Omnibus	GSE220156
Metabolomics data	FigShare	https://doi.org/10.6084/m9.figshare.22246258
Experimental models: Cell lines		
Patient-dervied BM lines	This manuscript	N/A
Neural Stem Cells	This manuscript	N/A
CRUK0748, CRUK0733	Provided by Prof. Charles Swanton	N/A

(*Continued on next page*)

Continued

REAGENT or RESOURCE	SOURCE	IDENTIFIER
Experimental models: Organisms/strains		
NOD-scid IL2Rgammanull (NSG) Mouse	The Jackson Laboratory	RRID:IMSR_JAX:005557
Recombinant DNA		
pMD2.G	Addgene	Cat#12259; RRID:Addgene_12259
psPAX2	Addgene	Cat#12260; RRID:Addgene_12260
Toronto KnockOut (TKO) CRISPR Library - Version 3	Addgene	Cat#:90294; RRID:Addgene_52961
Firefly Luciferase	Addgene	RRID:Addgene_118017
Software and algorithms		
Image Scope	Aperio	https://aperio-imagescope.software.informer.com
Living Image	Perkin Elmer	https://www.perkinelmer.com/es/lab-products-and-services/resourcesin-vivo-imaging-software-downloads.html
R	The R project for Statistical Computing	https://www.r-project.org/
GraphPad Prism	Graph Pad	https://www.graphpad.com/
ImageJ	NIH	https://imagej.nih.gov/
FlowJo v10.8	FlowJo LLC	https://www.flowjo.com/
BioRender	BioRender	https://biorender.com/
ELDA: Extreme Limiting Dilution Analysis	Walter+Eliza Hall Bioinformatics -Insitute of Medical Research	https://bioinf.wehi.edu.au/software/elda/
Aperio ScanScope	Leica Biosystems	N/A
FLUOstar Omega Fluorescence 556 Microplate reader	BMG labtech	N/A
Other		
10 mL Hamilton syringe	Hamilton	Cat#76350-01
NeuroCult NS-A Proliferation Kit	Stem Cell Technologies	Cat#05751

EXPERIMENTAL MODEL AND STUDY PARTICIPANT DETAILS

Cell culture

BM cell lines from primary lung (BT478, BT530), breast (BT923, BT930) and melanoma (BT673, BT917) cancers are derived from primary patient samples with written consent from the patients and approved by the Hamilton Health Sciences McMaster Health Sciences Research Ethics Board (REB #07366), in compliance with Canada's Tri-Council Policy Statement on the Ethical Conduct for Research Involving Humans and International Ethical guidelines for Biomedical Research Involving Human Subjects. MDA-MB-231 was purchasedfrom American Type Culture Collection and used to generate a brain metastasis derivate following injection into the mammary fat pad and isolation from the brain at human endpoint. Cell lines are maintained in NeuroCult Complete (NCC) media consisting of NeuroCult NS-A Basal Medium (Stemcell technology #05750) and supplemented with 50 mL of NeuroCult Supplement, 20 ng/mL epidermal growth factor (EGF), 10 ng/mL fibroblast growth factor (FGF), 2 μg/mL heparin and 1% penicillin-streptomycin. Human fetal neural stem cells (hNSCs) are derived in-house using a previously described protocol.[62] Normal human astrocytes are purchased from American Type Culture Collection. All cell lines were maintained at 37°C with a humidified atmosphere of 5% CO_2.

Patient-derived primary lung tumor cell lines CRUK0748 and CRUK0733 were obtained as kind gifts from our collaborator Prof. Charles Swanton. We tagged both cell lines with firefly luciferase and used our orthotopic lung-BM animal model to anticipate BM formation (or lack thereof) as was seen in the human patients from which the samples were biopsied from. Following orthotopic injection of cells, mice succumb to primary tumor burden before their brain tumors can grow to a fatal size.[12,63] Nonetheless, we used *ex vivo* bioluminescent imaging to confirm that the mice orthotopically injected with (metastatic) CRUK0748 cells developed BM prior to succumbing to their primary lung tumor burden, while the mice injected with (non-metastatic) CRUK0733 cells did not.

In vivo preclinical studies

All animal experiments were performed in accordance with the Canadian Council on Animal Care (CCAC) under animal utilization protocol (19-01-01) approved by the Animal Research Ethics Board (AREB). Human tissues were isolated using protocols approved by the Human Integrated Research Ethics Board (HIREB).

Sex considerations were factored into our research design and analysis. To the best of our knowledge, no studies have found any sex-related differences pertaining to response to an IMPDH inhibitor treatment. However, apart from breast cancer, significantly higher rates of brain metastases have been reported in males compared to females in nearly all primary cancer types. In this study, we included both male and female mice.

All experimental procedures involving animal work has been reviewed and approved by McMaster University Animal Research Ethics Board. Non-obese diabetic-severe combined immunodeficient IL2rγ^{null} (NSG) mice are used for all experiments. Equal numbers of male and female mice were used for all experiments. Healthy mice were 6–8 weeks at time of use. Mice are anesthetized by gas anesthesia using isoflurane (4% induction, 2.5% maintenance) before procedure. Cells were engineered to express firefly luciferase and were injected intracardially, orthotopically, or intracranially as previously described.[13,14,63–65] MPA and Compound **3** were administered by oral gavage (100 mg/kg). Mice are monitored weekly for signs of illness until endpoint.

METHOD DETAILS

Connectivity Map analysis

The Broad Institute's original CMap was used to identify possible drug candidates that could affect the expression of the deregulated genes revealed by transcriptome analyses of premetastatic BMICs. Over 1200 small molecules are assessed in this analysis using Bioconductor package PharmoacoGx. This analysis reveals 380 drugs with the ability to affect one or more of the 3951 deregulated genes in lung-, breast-, and melanoma-BMICs. Drugs are filtered by resulting connectivity score (connectivity score <0, to denote their ability to revert gene deregulation) and associated significance ($p < 0.01$). The 48 drugs that fit these criteria are sourced and assayed in a preliminary drug screen to determine which drugs are effective in inhibiting BMIC proliferation. The lung-BMIC RNA-Seq data used for this analysis has been previously described and accessible through GEO Series accession number GSE110495.[13] The breast- and melanoma-BMIC RNA-Seq data used for this analysis has also been previously described and is accessible through GEO Series accession number GSE220156.[14]

In vitro functional assays

For preliminary drug screening, drugs were plated at a concentration of 10 μM in a 96-well plate, in triplicate at a density of 1000 cells/well, and incubated at 37°C with a humidified atmosphere of 5% CO_2 for three days. Vehicle controls for cell death are used in each functional experiment. Following treatment, PrestoBlue (20 μL, Invitrogen), a resazurin-based cell viability reagent and fluorescence indicator of cell metabolism, was added to each well to estimate proliferation approximately 2 h prior to measuring fluorescence intensity via FLUOstar Omega Fluorescence 556 Microplate reader (BMG LABTECH) at an excitation and emission wavelength of 540 nm and 590 nm, respectively. Results were analyzed using Omega analysis software.

Dose-response assays were conducted using the same protocol apart from drugs being plated using 2-fold serial dilutions (20 μM - 39 nM) as previously described.[66] The half maximal inhibitory concentration (IC_{50}) is determined by plotting percent cell viability by the logarithmic concentration of drug. IC_{80} concentrations will be used for subsequent functional assays (cell proliferation, sphere formation), as determined by the following formula:

$$IC_F = \frac{100 - F^{\frac{1}{H}}}{F} \times IC_{50}$$

where F = fraction of maximal response and H = hill slope.

Cell proliferation assays were conducted using the same protocol, except for drugs being plated at their IC_{80} for a four-day incubation period. All results were illustrated and analyzed for significance using GraphPad Prism 8 software.

Clonogenic sphere formation assays

To assess tumor sphere forming capacity under clonogenic conditions, neurospheres were dissociated into single cells and plated at a low density of 200 cells/well in low-binding tissue culture-treated 96-well plates in serum-free media as previously described.[14,66–69] Cells were incubated with drug (IC_{80}) or DMSO control, in triplicate, and incubated at 37°C with a humidified atmosphere of 5% CO_2. The number of spheres per well were manually counted seven days later. The results were illustrated and analyzed for significance using GraphPad Prism 8 software.

Limiting dilution assays

In the limiting dilution assay, cells were plated at a range of different cell concentrations (200 cells/well- 1 cell/well) in a low-binding 96-well plate in triplicates. The plate was incubated at 37°C with a humidified atmosphere of 5% CO2. Seven days later, the number of wells per condition that contained sphere-colonies under 10× magnification were counted. The frequency of BMICs within a given cell population was determined by linear regression analysis. Data was displayed as a scatterplot graph and the corresponding trend line; on the Y axis the percentage of wells without detectable spheres and on the X axis the number of seeded cells per well. Based on the Poisson distribution, the frequency of BMICs in the sample is the value corresponding to 37% of wells without detectable spheres.[23,70]

Migration assay

Cells were plated at a density of 15,000 to 25,000 cells (depending upon the cell line) per 70 μL media supplemented with 10% FBS into two separate wells of a bi-silicon structure within a 48-well plate. The cells were allowed to adhere for 24 h at 37C within a humidified atmosphere of 5% CO_2 to form a monolayer of cells. After 24 h the silicon inserts were detached from the 48-well plate, leaving behind two monolayers of cells that are separated by an empty 'wound' and enabling of cell migration into the exclusion zone. The media was then removed from the well and the cells were washed with pre-warmed PBS and replenished with 1 mL of media containing 250 nm SYTOX green, 2.5% FBS and MPA (IC_{80}) or vehicle control. The plate was inserted into the Incucyte *in vitro* imaging system where the 'wound' was imaged periodically over time. The Incucyte scan type chosen was 300ms adherent cell-by-cell alongside phase and green imaging channels within a 10× objective. The images were uploaded to ImageJ where the wound area was calculated to determine the wound coverage percentage.

Compounds

Chemical shifts in 1H NMR and 13C NMR spectra are reported in parts per million (ppm) relative to tetramethylsilane (TMS), with calibration to TMS (d_H, d_C 0.0) or the residual solvent peaks according to values reported by Gottlieb et al. (chloroform: d_H 7.26, d_C 77.16).[71] When peak multiplicities are given, the[72] following abbreviations are used: s, singlet; d, doublet; t, triplet; q, quartet; sept., septet; dd, doublet of doublets; m, multiplet; br, broad; app., apparent; gem, geminal. 1H NMR spectra were acquired at 400 or 700 MHz with a default digital resolution (Brüker parameter: FIDRES) of 0.22 and 0.15 Hz/point, respectively. Coupling constants reported herein therefore have uncertainties of ±0.4 Hz and ±0.3 Hz, respectively. All assignments of protons and carbons relied on data from 2-dimensional NMR experiments including COSY, HMQC, and HMBC. The 13C NMR spectra provided herein (13C{1H} DEPTQ-135; Brüker pulse program deptqgpsp) show CH and CH3 carbon signals below the baseline and C and CH2 carbons above the baseline. Melting points (mp) are uncorrected. Reactions were carried out at room temperature (rt) if temperature is not specified. Compounds purified by normal-phase flash chromatography[73] used Teledyne CombiFlash Rf+ and NextGen 300+ purification systems (www.teledyneisco.com) with pre-packed silica cartridges (either 40–63 mM or 20–40 mM particle size). High-resolution mass spectrometry (HRMS) data was obtained using a Brüker micrOTOF II system with electrospray ionization (ESI) and paired with an Agilent HPLC and UV detector.

Mycophenolic acid (MPA, [24280-93-1]) was purchased from AmBeed (Arlington Heights, Illinois, www.ambeed.com). Mycophenolate mofetil (MMF, [128794-94-5]) and 2-aminoethylpyrrolidine were purchased from Aaron Chemicals (San Diego, California, www.aaronchem.com). tert-Butyldimethylchlorosilane (TBS-Cl) and N-methylpiperazine were purchased from Oakwood Chemical (Estill, South Carolina, www.oakwoodchemical.com). 1-Boc-4-(2-hydroxyethyl)piperazine, N-(2-hydroxyethyl)pyrrolidine, 4-dimethylaminopyridine (DMAP), and 1-(3-dimethylaminopropyl)-3-ethylcarbodiimide hydrochloride (EDC·HCl) were purchased from AK Scientific (Union City, California, www.aksci.com). N-(3-Aminopropyl)azetidine was purchased from Enamine (Kyiv, Ukraine, www.enaminestore.com). Tetrabutylammonium fluoride (TBAF) was purchased as a solution in THF from Sigma-Aldrich (www.sigmaaldrich.com). Imidazole was purchased from Fisher Scientific (www.fishersci.ca). Chemical syntheses are outlined in the supplementary information.

PAMPA assay

The permeability of the compounds was evaluated using the parallel artificial membrane permeability (PAMPA) assay, as previously described.[35] The PAMPA assembly was consisted of the acceptor plate (MultiScreen IP Filter Plate, Millipore Sigma, Canada) and the donor plate (96 well Collection Plate, Millipore Sigma, Canada). The artificial membrane solution was prepared as 15 mg/mL of polar brain lipid from porcine (Sigma Aldrich, Oakville, Canada) in a solution of 60% chloroform/40% dodecane. Seven μL of the mixture was pipetted into each acceptor plate well (top compartment). Thereafter, 300 μL of PBS (1 × PBS, pH 7.4, 5% DMSO) solution was added to each well of the acceptor plate and 300 μL of drug-containing donor solutions (50 μM compounds in 1 × PBS, pH 7.4, 5% DMSO) to each well of the donor plate (bottom compartment) in triplicate. The acceptor plate was placed into the donor plate and the assembly was incubated at room temperature for 16 h. After incubation, aliquots of 10 μL from each well of acceptor and donor plate were transferred into a 96-well plate and 190 μL of acetonitrile (containing IS: 300 nM Dexamethasone, 100 nM Phenacetin), was added into each well. The plate was vortexed at 750 rpm for 2 min and was centrifuged at 7,000 g for 10 min. The concentration of the compounds was determined by LC/MS/MS. The effective permeability (P_e), in units of centimeter per second, was calculated using the following equation:

$$Log\,P_e = Log\left\{C \times \left[-Ln\left(1 - \frac{[drug]_{acceptor}}{[drug]_{equilibrium}}\right)\right]\right\}$$

Where: C = VD × VA/[(VD + VA) × t × A]; VD = volume of donor compartment (0.30 mL); VA = volume of acceptor compartment (0.30 mL); A = filter area (0.24 cm^2 for Multi-Screen Permeability Filter plate); and t = incubation time (in seconds).

MDR1-MDCKII assays

MDR1-MDCKII permeability assays were performed by Wuxi AppTec Co. Briefly, MDR1-MDCK1 cells were seeded onto polycarbonate membranes in 96-well insert system plates and allowed to develop into monolayers. MPA and Compound 3 (2 μM in

10 mM HEPES pH 7.4, 1% DMSO) were applied to either the apical or basolateral side of the monolayer. The plate was incubated for 2.5 h (37°C, 5% CO2) and the media was sampled on either side and the compound present was quantified by LC-MS/MS and the concentrations were used to calculate the efflux ratio. Digoxin (10 μM), nadolol (2 μM), and metoprolol (2 μM) were used as controls.

Brain tissue binding assay

Brain homogenate binding assays were performed by Wuxi AppTec Co. Briefly, CD-1 pooled mouse brain homogenate (Cat: MSE00BRAINYZA) was obtained and treated with MPA or Compound 3 at a final concentration of 2 μM. The samples were applied to a dialysis well plate and sealed with dialysis membrane. Dialysis buffer (100 mM sodium phosphate pH 7.4, 150 mM NaCl) was applied to opposite side of the membrane (receiver well of the plate), and samples were incubated at 37°C with 5% CO2 for 4 h. Following completion of incubation period, samples were recovered from each side of the dialysis membrane and processed by protein precipitation, and analysis by LC-MS/MS for compound concentration. A corresponding set of samples was also prepared and stop solution (acetonitrile containing 200 ng/mL tolbutamide and 200 ng/mL labetalol) was immediately added, and samples were obtained from each side of the dialysis plate for mass spectrometric analysis.

Western Blot

Protein lysates were loaded and resolved on sodium dodecyl sulfate (SDS) polyacrylamide gel followed by electro-transfer onto a polyvinylidene difluoride (PVDF) membrane. Protein concentrations were quantified using the Bradford Assay (BioRad). Membranes were blocked with 100% methanol for 30 s, allowed to dry at room temperature, and incubated with the respective antibody overnight at 4°C. Mouse monoclonal anti-IMPDH (Santa Cruz Biotechnology; catalog # sc:166551) and mouse monoclonal anti-GAPDH (Abcam; cataologue #ab8245) were used at a 1:1000 dilution. For development, the anti-IMPDH antibody was used with ThermoFisher SuperSignal West Femto Maximum Sensitivity Substrate while the anti-GAPDH antibody was used with RioRad Clarify ECL reagents. Immunoblots were visualized with ImageLab software.

Firefly-luciferase lentivirus generation

A lentiviral vector expressing Firefly Luciferase (Addgene, RRID:Addgene_118017) was used for this study. Replication-incompetent lentivirus was produced by co-transfection of the Firefly Luciferase vector and packing vectors pMD2G and psPAX2 in HEK293T cells at ~80% confluency using Lipofectamine 3000 reagent (ThermoFisher) as per manufacturer's instructions. Viral supernatant was harvested every 24 h for a total of three days and concentrated by PEGit (System Biosciences) as per manufacturer's instructions. The viral pellet was resuspended in 1.0 mL of DMEM, aliquoted, and stored at −80°C. BMIC lines were transduced with lentiviral vectors and treated with puromycin after 48 h of transduction as a selection marker to develop stable cell lines.

In vivo imaging

Bioluminescent imaging was performed using an IVIS Spectrum *In Vivo* Imaging System (PerkinElmer) as per the manufacturer's instructions. Imaging and quantification of signals is controlled by the analysis software Living Image (Xenogen). Mice were weighed and injected intraperitoneally with 10μL/g of 15 mg/mL solution of D-Luciferin firefly solution (PerkinElmer) in phosphate buffered saline (Invitrogen) 10 min before being imaged, and anesthetized (4% induction, 2.5% maintenance isoflurane). Mice were then placed onto a warmed stage inside the instrument and imaged for a maximum of 3 min depending on the tumor size. Regions of interest were quantified by bioluminescent signal (photons per second) using Living Image software for a standardized comparison between images.

Fluorescence-activated cell sorting

BMICs were dissociated into single cell suspensions and resuspended in phosphate buffered saline (PBS, Wisent Bio) with 2 mM EDTA. Cells were stained with APC-conjugated anti-human TRA-1-85 (CD147; Cat # 130-128-900, Miltenyi Biotec) and incubated for 15 min at room temperature. The viability dye 7-Aminoactinomycin D (7-AAD; Cat # 00-6993-50, eBioscience) was used to exclude dead cells; incubation with 7-AAD allows for penetration of compromised membranes and binding to DNA.[74] Live cells were analyzed using Summit 5.4 software on MoFlo XDP cell sorter (Beckman Coulter) to confirm human BMIC metastasis to the brain.

Metabolomics mass spectrometry

For metabolomics profiling, 10^6 cells (BT478, BT530, NHAs) were cultured in the presence of MPA, Compound 3 (IC_{80}) or vehicle control, for 6 h, and then collected, washed with PBS, and flash frozen in liquid nitrogen. Metabolites were extracted with a solution of cold acetonitrile/methanol/water (2:2:1) from the cell pellets and protein precipitation was performed by three cycles of freeze/thawing and sonication. The LC-MS metabolomics analysis was performed as previously described.[75] Briefly, a UHPLC-MS system consisting of an Agilent 6550 qToF coupled to an Agilent 1290 binary pump UHPLC system was used. The source parameters were as follows: Gas temperature, 150 °C at 14 L/min and 45 psig; Sheath gas temperature, 325 °C at 12 L/min; Capillary and nozzle voltages were set to −2.0 kV iFunnel conditions were changed from default to-30 V DC, High pressure funnel drop −100 V and RF voltage of 110 V, low pressure funnel drop −50 V and RF voltage of 60 V. Chromatographic separation was achieved by ion-paired chromatography. In brief, 2 mL of each sample was injected onto Agilent ZORBAX Extend-C18 (150 mm 3 2.1 mm i.d.; 1.8 mm) column using

tributylamine (TBA) as an ion paring agent (solvent A: 3% methanol, 97% water 10 mM TBA, 15 mM Acetic acid, solvent B: 100% methanol). The linear gradient employed was as follows: 0–2.5 min 99% A, 2.5–7.5 min decrease to 80% A, 7.5–13 min to 55% B and finally 13–15 min to 99% B and held for 1 min. The column was re-generated for 2 min at 1% B. The flow rate was set to 250 mL/min. The column temperature was maintained at 25°C. Skyline was used for data processing of metabolites in our library of standards using known retention times and MS/MS spectra. Integrated peak areas for the metabolites was exported for further statistical and metabolite enrichment analysis by using online MetaboAnalyst (https://www.metaboanalyst.ca/).

Generation of IMPDH knockout lines

Guide RNAs (gRNAs) targeting AAVS1 (5′-GGGGCCACTAGGGACAGGAT-3′) and IMPDH1 (5′- ACCGCGGTGTGTAACTCACAGCCA-3′) and IMPDH2 (5′-aCCGTCCATGGGAGAGGAAACCAG-3′) were obtained from TKOv3[76] and cloned into a single-gRNA lentiCRISPRv2 construct (Addgene 52961). Sequences were verified using Sanger sequencing. Each plasmid was packaged independently into lentivirus using second-generation packaging constructs as described previously.[77] BMICs were infected with lentivirus containing single-gRNA lentiCRISPRv2 constructs targeting AAVS1 or IMPDH1 or IMPDH2 (three gRNAs). Twenty-four hours post-infection, virus-containing media was replaced with fresh media containing puromycin (1–2 μg/mL) (ThermoFisher, Cat#A1113803) for 48–72 h. The knockout efficiency was validated by Western Blotting for evaluation of IMPDH protein expression.

QUANTIFICATION AND STATISTICAL ANALYSIS

Statistical analysis

Replicates from at minimum three samples are used for all applicable experiments for mean comparisons. Data collected from respective *in vitro* experiments are represented using GraphPad Prism 8 software. Student t tests and two-way ANOVA analyses are conducted using the same software, with a p-value <0.05 deemed as statistically significant. For *in vivo* studies, medium survival differences were measured using Kaplan-Meier survival curves and significance determined by the Log-rank test.

Cell Reports Medicine, Volume *5*

Supplemental information

De novo GTP synthesis is a metabolic vulnerability for the interception of brain metastases

Agata M. Kieliszek, Daniel Mobilio, Blessing I. Bassey-Archibong, Jarrod W. Johnson, Mathew L. Piotrowski, Elvin D. de Araujo, Abootaleb Sedighi, Nikoo Aghaei, Laura Escudero, Patrick Ang, William D. Gwynne, Cunjie Zhang, Andrew Quaile, Dillon McKenna, Minomi Subapanditha, Tomas Tokar, Muhammad Vaseem Shaikh, Kui Zhai, Shawn C. Chafe, Patrick T. Gunning, J. Rafael Montenegro-Burke, Chitra Venugopal, Jakob Magolan, and Sheila K. Singh

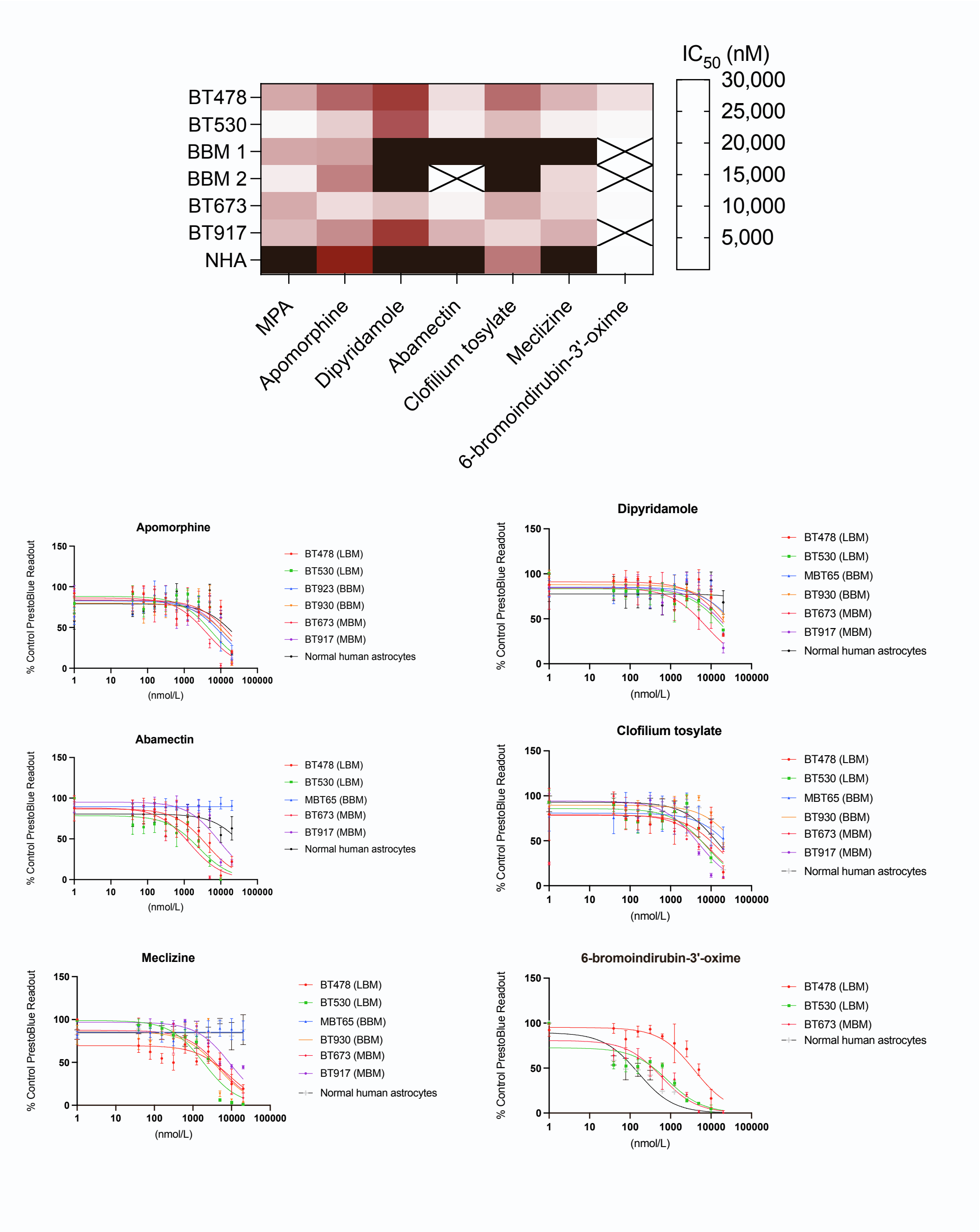

Fig S1. Dose-response curves for effective anti-BMIC compounds, related to Figure 1. Seven compounds shown anti-BMIC activity against a patient-derived lung-BMIC line (BT478) in a preliminary screen of 48 CMap-reveled compounds (*see Fig 1e).* All seven compounds were explored in dose-response assays against multiple BMIC lines and normal human astrocytes. Activity in these assays is summarized in a heat map.

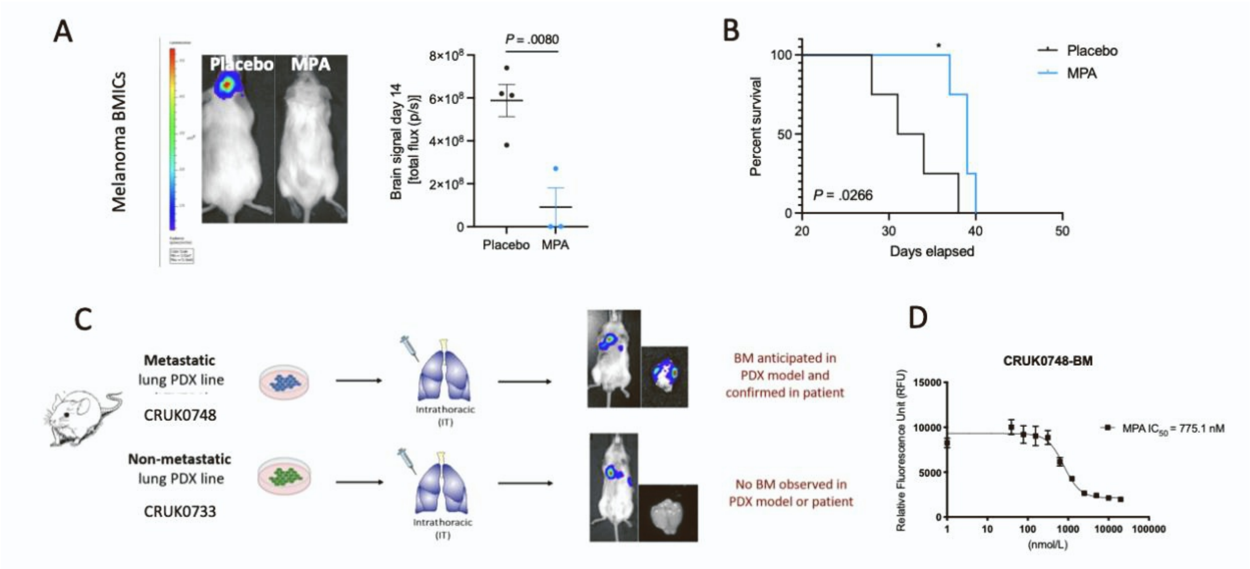

Fig S2. PDX models mimic patient data, related to Figure 3. a, Representative IVIS bioluminescence images of mice 14-days post injection and brain tumor burden comparisons between placebo and MPA groups. IVIS = *In vivo* imaging system. Comparisons were made via a two-tailed unpaired t-test and data are presented as mean ± SED from 4-5 technical replicates. *P* value is indicated. **b** *Kaplan-Meier* survival analysis of placebo and MPA groups following intracranial engraftment of patient-derived BMICs. Comparisons were made via a Log-rank (Mantel-Cox) test, *P* value is indicated**. c**, CRUK0748, a patient- derived lung tumor cell line that metastasized to the brain of the patient it was retrieved from, was confirmed to metastasize to the brains of mice in our animal models following intrathoracic injection. CRUK0733, a patient-derived lung tumor cell line that has not metastasized to the brain of the patient it was retrieved from, did not metastasize in our animal model**. d,** MPA demonstrates a dose-response anti-proliferative effect on BMICs isolated from the brains of mice following CRUK0748 metastasis to the brain**.**

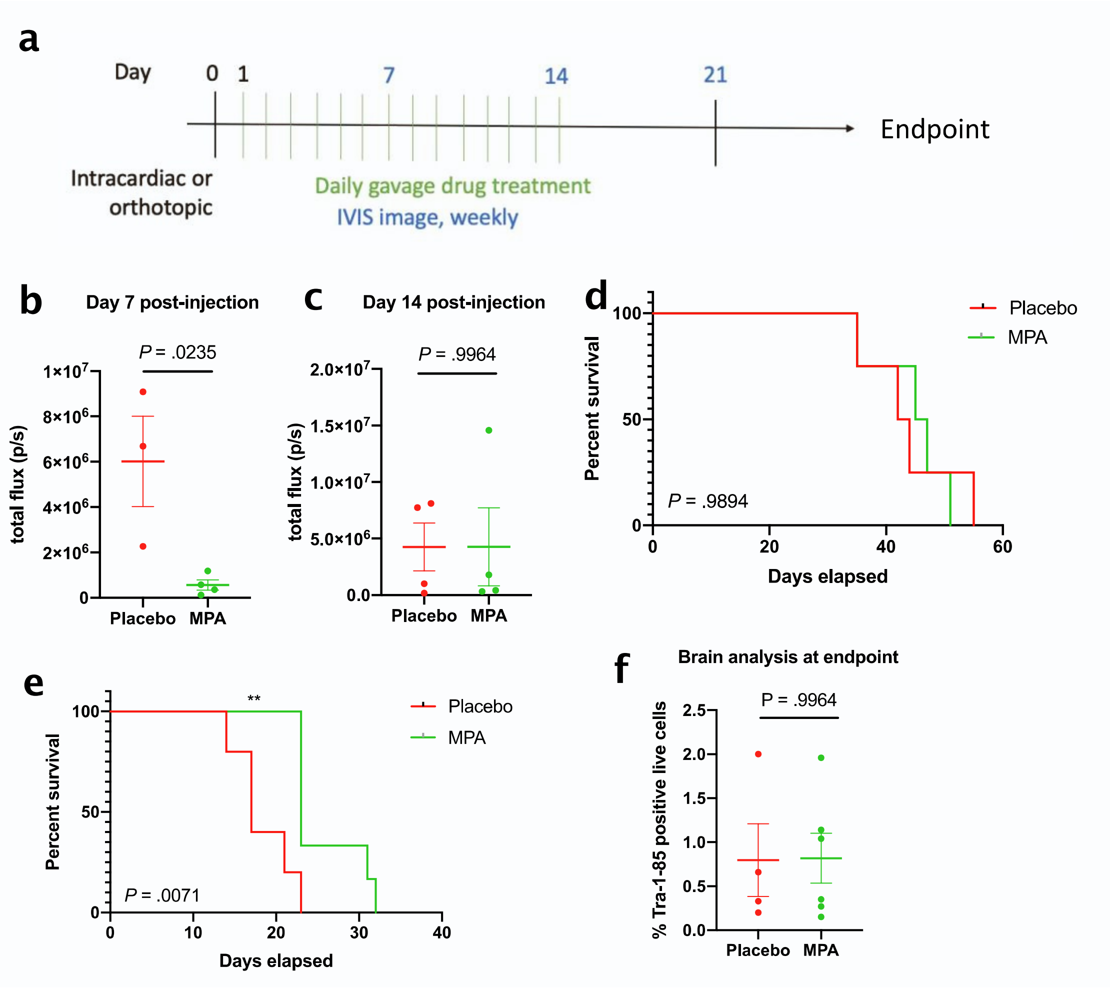

Fig S3. MPA must cross the blood brain barrier to effectively slow the progression of BM over time, related to Figures 3 and 4. a, Mice were either orthotopically (intrathoracically) or intracardiac injected with lung BMICs, followed by daily oral gavage administration of MPA (100 mg/kg) or placebo for 14 days. b, Following intracardiac-injection and subsequent treatment, mice treated with MPA showed a significant decrease in brain tumor burden seven days post-injection by IVIS, (c), no significant reduction in tumor burden following treatment completion (IVIS), and (d) did not experience a survival benefit compared to placebo. e, Following orthotopic injection and subsequent treatment as described In (a), mice treated with MPA showed a significant increase in survival compared to placebo, but (f) had the same number of human cells detected in their brains following humane endpoint by flow cytometry. n.s. = not significant.

Table S1: Bi-directional permeability via MDR1-MDCKII cells models, related to Figure 4. To explore active transport/efflux mechanisms, we employed a MDCK-MDR1 cell monolayer model. Metoprolol is used as a control compound with high permeability and nadolol is used as a control compound with low permeability.

	Mean P_{app} (10^{-6} cm/s) A to B	Mean P_{app} (10^{-6} cm/s) B to A	Efflux Ratio	Rank of P_{app}
**Mycophenolic Acid** (MPA)	26.0	10.0	0.384	High
**Compound 3**	14.8	12.5	0.848	High
**Digoxin** (P-gp substrate)	0.556	11.8	21.2	Low
**Nadolol** (Low permeability)	0.198	Not Detected	-	Low
**Metoprolol** (High Permeability)	29.5	Not Detected	-	High

Table S2: Brain homogenate binding, related Figure 4. Mouse brain homogenate was treated with each compound followed by equilibrium dialysis and mass spectrometry to determine the fraction of bound (65.9%) and un-bound drug (34.1%) for MPA. The mass of Compound **3** could not be detected following completion of the assay, and a proper bound/unbound fraction could not be determined.

Brain homogenate binding

	% Unbound	% Bound
**Mycophenolic Acid** (MPA)	34.1 ± 3.0	65.9 ± 3.8
**Compound 3**	Not Recovered	Not Recovered
**Propranolol** (Control Compound with High Binding)	2.3 ± 0.0	97.6 ± 0.3

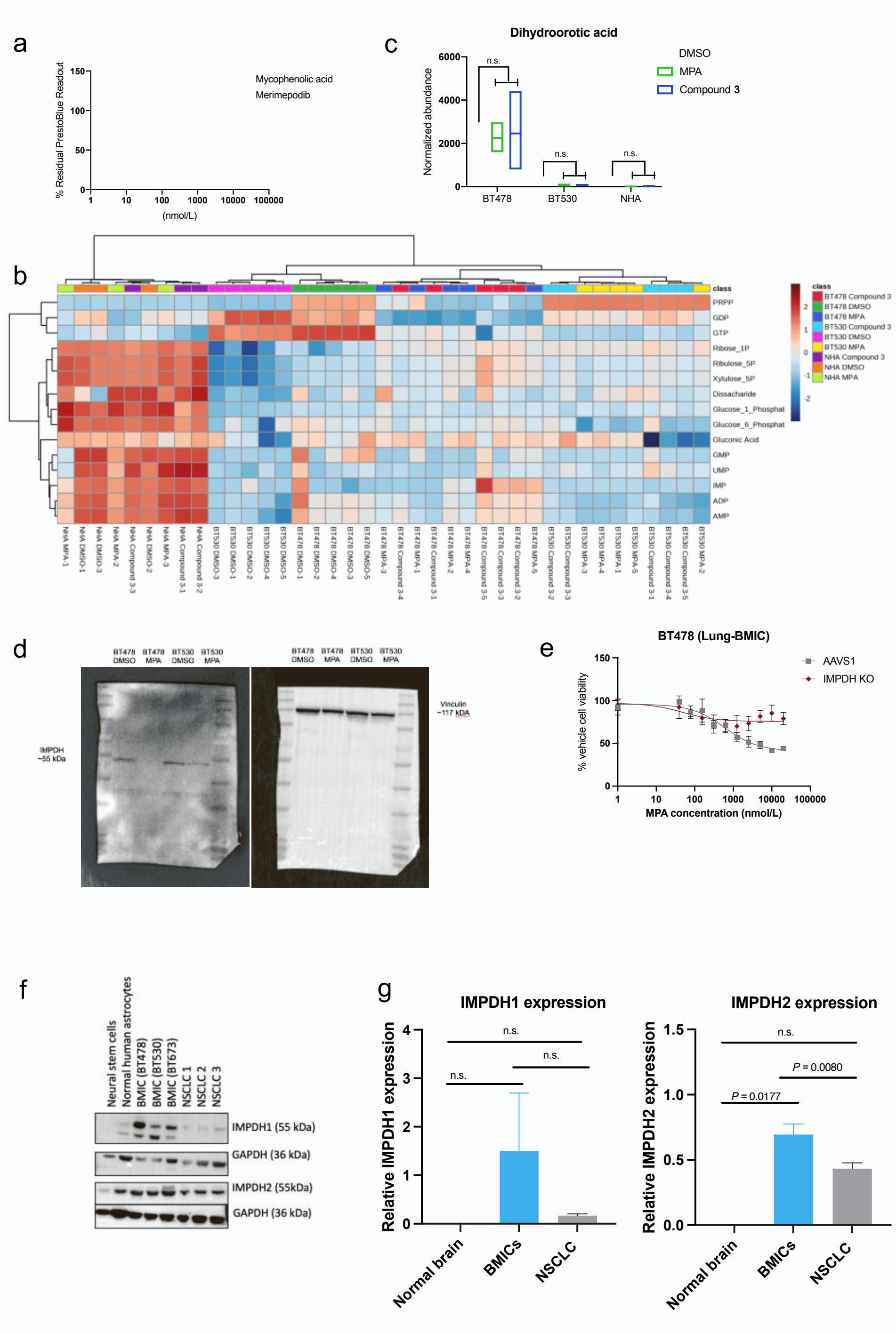

Fig S4. Mechanistic experiments, related to Figure 5. a, Comparative dose-response assay against a patient-derived lung BMIC (BT478) using mycophenolic acid and merimepodib, a selective-IMPDH inhibitor. **b,** LCMS-based metabolomics of the differential polar metabolome of vehicle-, MPA-, or Compound 3 (MLSS2008)-treated BM cells in comparison to normal human astrocytes. Heatmap uses Euclidean distance with Ward's method *via* metaboanalyst. **c**, Box plots depicting relative dihydroorotic acid levels in patient-derived BMIC lines and normal human astrocytes (NHA) with either DMSO-, MPA-, or Compound 3-treatment. N.s. = not significant. **d,** Uncropped blots shown in Figure 5e. **e,** Dose-response assays using AAVS1 and IMPDH KO BT478 cells**. f**, Immunoblot of IMPDH1 and IMPDH2 in normal brain cells (neural stem cells and normal human astrocytes), primary non-small cell lung cancer (NSCLC) cells (CRUK0935, CRUK0883, CRUK0733), and patient-derived BMICs (BT478, BT530, BT673). Cropped from single full blot. **g,** quantification of western blot band intensity is normalized to loading control bands using ImageLab software.

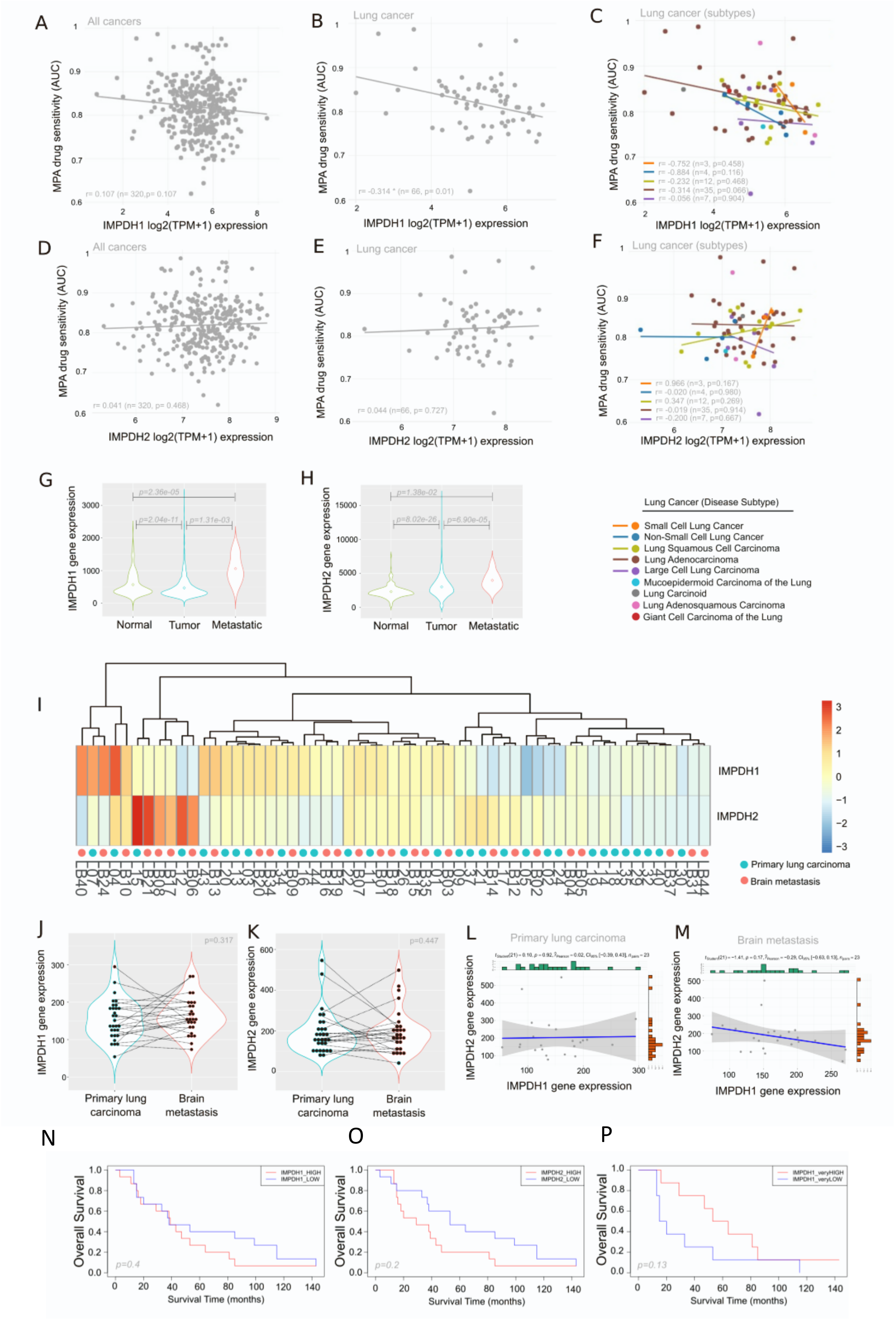

Fig S5. IMPDH as a potential biomarker for lung cancer, related to Figure 5.

(**a-f**) Correlation of mycophenolic acid (MPA) drug sensitivity (area under the dose-response curve (AUC)) based on IMPDH expression across all cancer cell lines (**a**, **d**), lung cancer cell lines (**b**, **e**) and lung cancer cell lines grouped by disease subtype (**c**, **f**) for IMPDH1 (**a-c**) and IMPDH2 (**d-f**) genes. The number of samples (*n*), the Pearson correlation coefficient (r) and p-value (p) are shown. Data was obtained from DepMap portal (Broad Institute) (Drug sensitivity AUC (PRISM repurposing secondary screen); Expression 22Q2 Public, log2(TPM + 1)). (**g-h**) Differential gene expression analysis in normal (*n* = 391), tumor (*n* = 1865) and metastatic (*n* = 8) patient samples (subset for lung tissue) for IMPDH1 (**g**) and IMPDH2 (**h**) gene expression. Krustal Wallis and Dunn test statistical analysis for non-parametric data as shown by TNMplot. Gene chip data were obtained from TNMplot.com. (**i**) Heatmap of IMPDH1 and IMPDH2 gene expression in a cohort of patients with primary lung carcinoma (NSCLC) (*n* = 30) that developed brain metastasis (*n* = 27). Scale (Z-score by gene). (**j-k**) Differential expression of paired primary lung carcinoma and brain metastasis patient samples (*n* = 23) for (**j**) IMPDH1 and (**k**) IMPDH2. Shapiro-Wilk normality test and Paired t-test were performed. (**l-m**) Correlation of IMPDH1 with IMPDH2 gene expression in primary lung carcinoma (*n* = 30) (**l**) and brain metastasis (*n* = 27) (**m**) patient samples. The number of samples (*n*), the Pearson correlation coefficient (r) and p-value (p) are shown. (**i-m**) Data was obtained from the processed (Q3 method normalization) GEO dataset (GSE200563). Data acquisition, analysis and visualisation performed using R version 4.1.2 and the following packages: GEOquery, pheatmap, ggplot2 and ggstatsplot. (**n-p**) Kaplan−Meier curves showing the overall survival probability (from primary cancer diagnosis to death) in a cohort of 30 patients with lung carcinoma that developed brain metastasis, for primary tumor IMPDH1 high vs low expression (median cut-off value: 144.72) (**n**), for IMPDH2 high vs low expression (median cut-off value: 162.75) (**o**), and for IMPDH1 very high vs very low expression (1st and 3rd Quartiles cut-off values: <116.19 and >185.31) (**p**). The Cox regression model for survival analysis was used, p-values (p) are shown. Data was obtained from the processed (Q3 method normalization) GEO dataset (GSE200563). Data acquisition, analysis and visualisation performed using R version 4.1.2 and the following packages: GEOquery, survival, and ggplot2.

Methods S1. Chemical Syntheses, related to Figure 4.

(*E*)-6-(4-*tert*-Butyldimethylsilyloxy-6-methoxy-7-methyl-3-oxo-1,3-dihydroisobenzofuran-5-yl)-4-methylhex-4-enoic acid (S1)

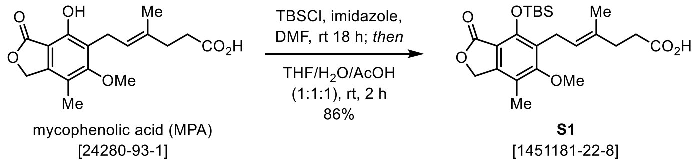

As a variation of the procedure developed by Cholewinski et al.,[1] DMF (20 mL, 0.3 M) was added under an atmosphere of argon to a 100 mL round bottom flask containing mycophenolic acid (2.0 g, 6.24 mmol, 1 equiv). To the solution was added imidazole (2.76 g, 40.6 mmol, 6.5 equiv) followed by TBSCl (3.76 g, 24.96 mmol, 4.0 equiv) and the reaction mixture was stirred at room temperature for 18 hours. The reaction mixture was diluted with water (42 mL) and EtOAc (30 mL). The layers were separated and the aqueous was extracted with ethyl acetate (30 mL). The combined organics were washed once with 1% HCl and three times with water, dried with Na_2SO_4, and concentrated under reduced pressure to obtain a colourless oil. The oil was then dissolved in a 1:1:1 solvent mixture of THF:H_2O:AcOH (30 mL) and stirred at room temperature for two hours. The reaction was diluted with water and extracted with ethyl acetate. The combined organics were washed with water twice, dried with Na_2SO_4, and concentrated under reduced pressure to obtain a colourless oil which solidified under vacuum. The white solids were vacuum filtered and washed excessively with hexanes to obtain TBS-protected mycophenolic acid **S1** (2.34 g, 5.38 mmol, 86%) as a white solid without the need for further purification. R_f = 0.66 (60:39:1 EtOAc/Hex/AcOH). Mp 129–131 °C (lit.[Error! Bookmark not defined.] 94−96 °C). ^{1}H NMR (400 MHz, $CDCl_3$): d 5.20 (tq, *J* = 6.4, 1.3 Hz, 1H), 5.06 (s, 2H), 3.73 (s, 3H), 3.38 (d, *J* = 6.4 Hz, 2H), 2.44−2.36 (m, 2H), 2.33−2.24 (m, 2H), 2.15 (s, 3H), 1.75 (d, *J* = 1.3 Hz, 3H), 1.02 (s, 9H), 0.24 (s, 6H). ^{13}C NMR (100 MHz, $CDCl_3$) δ 179.1, 169.4, 163.3, 151.9, 146.2, 133.5, 127.7,

124.0, 118.1, 111.8, 67.8, 60.9, 34.2, 32.8, 26.2 (3C), 23.8, 18.9, 16.5, 11.6, −3.4 (2C). LCMS (ESI) *m/z*: 457.2017 calcd for $C_{23}H_{34}O_6SiNa^+$ [M + Na]$^+$; Found 457.2038. Spectral data were consistent with those previously reported.Error! Bookmark not defined.

2-(4-Boc-piperazino)ethyl (*E*)-6-(4-*tert*-Butyldimethylsilyloxy-6-methoxy-7-methyl-3-oxo-1,3-dihydroisobenzofuran-5-yl)-4-methylhex-4-enoate (S2)

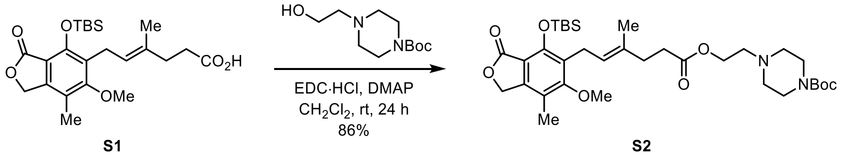

To a 10 mL round bottom flask containing TBS-protected mycophenolic acid **S1** (0.200 g, 0.46 mmol, 1 equiv) under an atmosphere of argon was added CH_2Cl_2 (2 mL, 0.23 M). To the solution was added 4-(2-hydroxyethyl)-1-Boc-piperazine (0.12 g, 0.55 mmol, 1.2 equiv), DMAP (0.005 g, 0.046 mmol, 0.1 equiv), and EDC·HCl (0.10 g, 0.55 mmol, 1.2 equiv) and the reaction was stirred for 17 hours. The reaction mixture was concentrated under reduced pressure and diluted with water ethyl acetate. The layers were separated and the aqueous was extracted with ethyl acetate. The combined organics were washed twice with water and brine, dried with Na_2SO_4, and concentrated under reduced pressure. The product was purified by column chromatography (85% EtOAc/Hexanes) providing the ester **S2** as a colourless oil (0.26 g, 0.4 mmol, 86%). R_f = 0.25 (70% EtOAc/Hex). ^{1}H NMR (400 MHz, $CDCl_3$): d 5.18 (tq, *J* = 6.4, 1.4 Hz, 1H), 5.07 (s, 2H), 4.15 (t, *J* = 5.9 Hz, 2H), 3.74 (s, 3H), 3.42−3.37 (m, 6H), 2.58 (t, *J* = 5.9 Hz, 2H), 2.45−2.34 (m, 6H), 2.31−2.24 (m, 2H), 2.15 (s, 3H), 1.75 (d, *J* = 1.4 Hz, 3H), 1.45 (s, 9H), 1.03 (s, 9H), 0.24 (s, 6H). ^{13}C NMR (100 MHz, $CDCl_3$): d 173.2, 169.2, 163.2, 154.7, 151.7, 146.1, 133.7, 127.6, 123.6, 118.0, 111.6, 79.6, 67.6, 61.6, 60.7, 56.7, 53.1 (2C), 44.1 (br), 43.0 (br), 34.4, 33.0, 28.4 (3C), 26.1 (3C), 18.7, 16.4, 11.4, −3.5 (2C). LCMS (ESI) *m/z*: 647.3722 calcd for $C_{34}H_{55}N_2O_8Si^+$ [M + H]$^+$; Found 647.3696.

Mycophenolic acid, 2-(4-Boc-piperazino)ethyl ester (2)

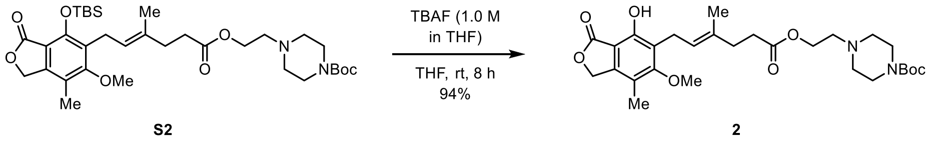

To a 5 mL round bottom flask containing **S2** (0.075 g, 0.12 mmol, 1 equiv) under an atmosphere of argon was added THF (0.6 mL, 0.23 M). TBAF as a 1.0 M solution in THF (0.14 mL, 0.14 mmol, 1.2 equiv) was added and the reaction stirred at room temperature for eight hours. The reaction was diluted with water and extracted with ethyl acetate. The combined organics were dried with Na_2SO_4 and concentrated under reduced pressure. The product was purified by column chromatography eluting with 85% EtOAc/Hexanes providing the free phenol **2** as a colourless oil (0.058 g, 0.1 mmol, 94%). R_f = 0.25 (70% EtOAc/Hex). ^{1}H NMR (400 MHz, $CDCl_3$): d 7.96 (brs, 1H), 5.0 (dq, *J* = 6.9, 1.4 Hz, 1H), 5.17 (s, 2H), 4.12 (t, *J* = 5.8 Hz, 2H), 3.75 (s, 3H), 3.44−3.38 (m, 4H), 3.36 (d, *J* = 6.9 Hz, 2H), 2.58 (t, *J* = 5.8 Hz, 2H), 2.47−2.37 (m, 6H), 2.31−2.24 (m, 2H), 2.13 (s, 3H), 1.78 (d, *J* = 1.4 Hz, 3H), 1.44 (s, 9H). ^{13}C NMR (100 MHz, $CDCl_3$): d 173.3, 172.9, 163.7, 154.8, 153.8, 144.2, 134.1, 123.0, 122.3, 116.7, 106.5, 79.7, 70.1, 62.0, 61.1, 56.8, 53.4 (2C), 44.2 (br), 43.0 (br), 34.8, 33.1, 28.5 (3C), 22.8, 16.2, 11.7. LCMS (ESI) *m/z*: 533.2857 calcd for $C_{28}H_{41}N_2O_8^+$ [M + H]$^+$; Found 533.2870.

2-(1-Pyrrolidinyl)ethyl (*E*)-6-(4-*tert*-Butyldimethylsilyloxy-6-methoxy-7-methyl-3-oxo-1,3-dihydroisobenzofuran-5-yl)-4-methylhex-4-enoate (S3)

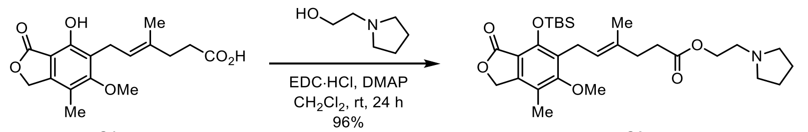

To a 100 mL round bottom flask containing TBS-protected mycophenolic acid **S1** (2.29 g, 5.28 mmol, 1 equiv) under an atmosphere of argon was added CH_2Cl_2 (23 mL, 0.23 M). To the solution, was added *N*-(2-hydroxyethyl)pyrrolidine (0.74 mL, 6.34 mmol, 1.2 equiv), DMAP (0.064 g, 0.53 mmol, 0.1 equiv), and EDC·HCl (1.21 g, 6.34 mmol, 1.2 equiv) and the reaction was stirred for 24 hours. The reaction mixture was concentrated under reduced pressure and diluted with 70 mL water and 70 mL ethyl acetate. The layers were separated and the aqueous was extracted with ethyl acetate. The combined organics were washed with water (2 × 40 mL), brine (2 × 40 mL), dried with Na_2SO_4, and concentrated under reduced pressure providing ester **S3** as a colourless oil (2.70 g, 5.07 mmol, 96%) that was used without any further purification. R_f = 0.23 (4% MeOH/CH_2Cl_2). ^{1}H NMR (400 MHz, $CDCl_3$): d 5.16 (tq, *J* = 5.1, 1.4 Hz, 1H), 5.04 (s, 2H), 4.15 (t, *J* = 5.9 Hz, 2H), 3.71 (s, 3H), 3.35 (d, *J* = 6.3 Hz, 2H), 2.71 (t, *J* = 5.9 Hz, 2H), 2.60−2.57 (brs, 4H), 2.40−2.36 (m, 2H), 2.28−2.24 (m, 2H), 2.13 (s, 3H), 1.82−1.76 (m, 4H), 1.73 (brs, 3H), 1.00 (s, 9H), 0.21 (s, 6H). ^{13}C NMR (100 MHz, $CDCl_3$): d 173.3, 169.2, 163.2, 151.7, 146.1, 133.7, 127.7, 123.6, 118.0, 111.7, 67.7, 62.9, 60.8, 54.6, 54.5, 34.4, 33.0, 26.1, 23.7, 23.5, 18.8, 16.4, 11.5, −3.4 (2C). LCMS (ESI) *m/z*: 532.3089 calcd for $C_{29}H_{46}N_2O_6Si^+$ [M + H]$^+$; Found 532.3111.

Mycophenolic acid, 2-(1-pyrrolidinyl)ethyl ester (3)

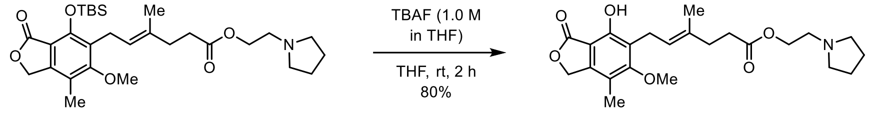

To a 100 mL round bottom flask containing **S3** (2.66 g, 5.0 mmol, 1 equiv) under an atmosphere of argon was added THF (22 mL, 0.23 M). TBAF as a 1.0 M solution in THF (6 mL, 6 mmol, 1.2 equiv) was added and the reaction stirred at room temperature for two hours. The reaction was diluted with water and extracted with ethyl acetate. The combined organics were dried with Na_2SO_4 and concentrated under reduced pressure. The product was purified by column chromatography eluting with 6% MeOH/DCM providing the free phenol **3** as a white solid (1.67 g, 4.0 mmol, 80%). R_f = 0.32 (6% MeOH/CH_2Cl_2). Mp 82–83 °C. ^{1}H NMR (400 MHz, $CDCl_3$): d 7.7−6.7 (brs, 1H), 5.21 (tq, *J* = 6.9, 1.3 Hz, 1H), 5.17 (s, 2H), 4.12 (t, *J* = 5.9 Hz, 2H), 3.75 (s, 3H), 3.37 (d, *J* = 6.9 Hz, 2H), 2.71 (t, *J* = 5.9 Hz, 2H), 2.63−2.54 (m, 4H), 2.44−2.38 (m, 2H), 2.33−2.26 (m, 2H), 2.13 (s, 3H), 1.82−1.75 (m, 7H). ^{13}C NMR (100 MHz, $CDCl_3$): d 173.5, 172.9, 163.7, 154.2, 144.2, 134.0, 123.2, 122.6, 116.4, 106.6, 70.0, 63.6, 61.1, 54.9 (2C), 54.6, 34.8, 33.2, 23.6 (2C), 22.8, 16.2, 11.6. LCMS (ESI) *m/z*: 418.2224 calcd for $C_{23}H_{32}NO_6^+$ [M + H]$^+$; Found 418.2234.

Mycophenolic acid, 4-methylpiperazine amide (4)

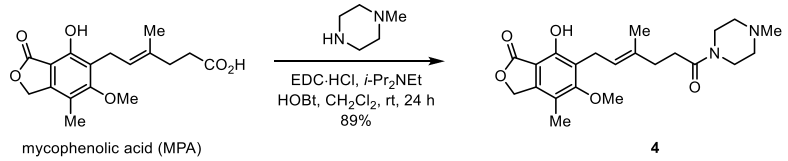

Diisopropylethylamine (165 mL, 0.95 mmol) was added to a suspension of mycophenolic acid (100 mg, 0.31 mmol), EDC·HCl (90 mg, 0.47 mmol), HOBt (63 mg, 0.47 mmol), and *N*-methylpiperidine (38 mg, 0.38 mmol) in CH_2Cl_2 (6 mL). After stirring the reaction mixture overnight at rt for 24 h, it was diluted with CH_2Cl_2 (20 mL), washed with saturated Na_2CO_3 (20 mL), dried over Na_2SO_4, and concentrated under reduced pressure. Flash chromatography (0→15% MeOH/CH_2Cl_2) provided amide **4** as a pale yellow oil (112 mg, 0.28 mmol, 89%). ^{1}H NMR (400 MHz, $CDCl_3$): d 5.22 (dd, *J* = 6.8, 1.3 Hz, 2H), 5.20 (s, 2H), 3.76 (s, 3H), 3.63−3.56 (m, 2H), 3.48−3.42 (m, 2H), 3.39 (d, *J* = 6.8 Hz, 2H), 2.43−2.26 (m, 8H), 2.29 (s, 3H), 2.15 (s, 3H), 1.82 (d, *J* = 1.3 Hz, 3H). ^{13}C NMR (100 MHz, $CDCl_3$): d 173.1, 171.3, 163.8, 153.8, 144.1, 135.0, 122.5, 122.4, 116.9, 106.6, 70.2, 61.2, 55.3, 54.9, 46.2, 45.6, 41.6, 35.2, 32.1, 22.8, 16.6, 11.7.

Mycophenolic acid, 3-(azetidin-1-yl)propyl amide (5)

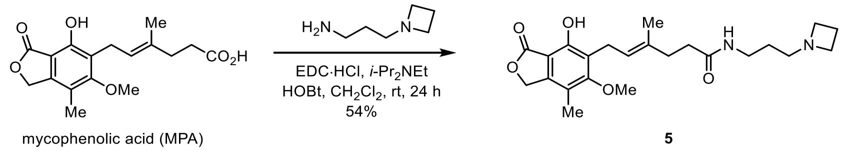

Diisopropylethylamine (165 mL, 0.95 mmol) was added to a suspension of mycophenolic acid (100 mg, 0.31 mmol), EDC·HCl (90 mg, 0.47 mmol), HOBt (63 mg, 0.47 mmol), and 3-(azetidin-1-yl)propylamine (42 mg, 0.37 mmol) in CH_2Cl_2 (6 mL). After stirring the reaction mixture overnight at rt for 24 h, it was diluted with CH_2Cl_2 (20 mL), washed with saturated Na_2CO_3 (20 mL), dried over Na_2SO_4, and concentrated under reduced pressure. Flash chromatography (0→10% MeOH/CH_2Cl_2) provided amide **5** as a pale yellow oil (70 mg, 0.17 mmol, 54%). ^{1}H NMR (400 MHz, $CDCl_3$): d 7.20 (t, *J* = 6.1 Hz, 1H), 5.21 (tq, *J* = 6.9, 1.5 Hz, 1H), 5.19 (s, 2H), 4.00 (appt, *J* = 7.3 Hz, 4H), 3.75 (s, 3H) 3.37 (d, *J* = 6.9 Hz, 2H), 3.28 (appq, *J* = 6.1 Hz, 2H), 3.06 (appt, *J* = 6.7 Hz, 2H), 2.52 (app pent, *J* = 8.0 Hz, 2H), 2.34−2.24 (m, 4H), 2.14 (s, 3H), 1.84 (pent, *J* = 6.5 Hz, 2H), 1.80 (s, 3H). ^{13}C NMR (100 MHz, $CDCl_3$): d 173.8, 173.0, 163.8, 153.9, 144.3, 134.8, 122.9, 122.4, 116.8, 106.6, 70.1, 61.2, 53.9 (2C), 52.8, 35.9, 35.3, 35.0, 24.2, 22.8, 16.4, 16.2, 11.7.

2-(1-Pyrrolidinyl)ethyl (*E*)-6-(4-*tert*-butyldimethylsilyloxy-6-methoxy-7-methyl-3-oxo-1,3-dihydroisobenzofuran-5-yl)-4-methylhex-4-enoate (S4)

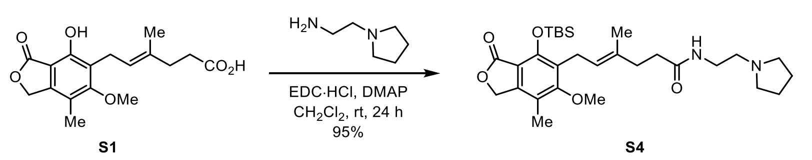

To a 10 mL round bottom flask containing TBS-protected mycophenolic acid **S1** (0.20 g, 0.46 mmol, 1 equiv) under an atmosphere of argon was added CH_2Cl_2 (2 mL, 0.23 M). To the solution was added 2-(pyrrolidin-1-yl)ethylamine (0.063 g, 0.55 mmol, 1.2 equiv), DMAP (0.005 g, 0.046 mmol, 0.1 equiv), and EDC·HCl (0.10 g,

0.55 mmol, 1.2 equiv) and the reaction was stirred for 17 hours. The reaction mixture was concentrated under reduced pressure and diluted with water ethyl acetate. The layers were separated and the aqueous was extracted with ethyl acetate. The combined organics were washed twice with water and brine, dried with Na_2SO_4, and concentrated under reduced pressure. The crude was purified by column chromatography (15% $MeOH/CH_2Cl_2$) providing amide **S4** a yellow oil (0.23 g, 0.43 mmol, 95%). R_f = 0.38 (20% $MeOH/CH_2Cl_2$). ^1H NMR (400 MHz, $CDCl_3$): d 7.04 (brs, 1H), 5.17 (brt, J = 6.4 Hz, 1H), 5.06 (s, 2H), 3.73 (s, 3H), 3.46 (appq, J = 5.5 Hz, 2H), 3.36 (d, J = 6.4 Hz, 2H), 3.03−2.92 (m, 4H), 2.89 (appt, J = 5.6 Hz, 2H), 2.35−2.26 (brs, 4H), 2.14 (s, 3H), 2.02−1.92 (m, 4H), 1.75 (d, J = 1.4 Hz, 3H), 1.01 (s, 9H), 0.22 (s, 6H). ^{13}C NMR (100 MHz, $CDCl_3$): d 173.4, 169.4, 163.4, 151.8, 146.2, 134.3, 127.8, 123.5, 118.1, 111.7, 67.8, 60.9, 55.5, 54.3 (2C), 36.6, 35.2 (2C), 26.2 (3C), 23.8, 23.5 (2C), 18.9, 16.5, 11.6, −3.4 (2C). LCMS (ESI) *m/z*: 531.3249 calcd for $C_{29}H_{47}N_2O_5Si^+$ [M + H]$^+$; Found 531.3244.

Mycophenolic acid, 2-(1-pyrrolidinyl)ethyl amide (6)

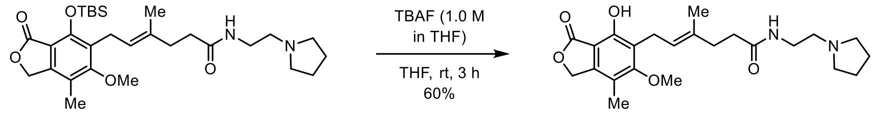

S4 **6**

To a 5 mL round bottom flask containing TBS-protected phenol **S4** (0.10 g, 0.19 mmol, 1 equiv) under an atmosphere of argon was added THF (0.94 mL, 0.23 M). TBAF as a 1.0 M solution in THF (0.23 mL, 0.23 mmol, 1.2 equiv) was added and the reaction stirred at room temperature for three hours. The reaction was diluted with water and extracted with ethyl acetate. The combined organics were dried with Na_2SO_4 and concentrated under reduced pressure. The product was purified by column chromatography eluting with 15% $MeOH/CH_2Cl_2$ providing the free phenol **6** as a white solid (0.047 g, 0.11 mmol, 60%). R_f = 0.38 (6% $MeOH/CH_2Cl_2$). ^1H NMR (400 MHz, $CDCl_3$): d 7.60 (brs, 1H), 5.23 (tq, J = 6.9, 1.4 Hz, 1H), 5.19 (s, 2H), 3.75 (s, 3H), 3.58 (appq, J = 5.5 Hz, 2H), 3.37 (d, J = 6.9 Hz, 2H), 3.33−3.10 (brs, 4H), 3.12 (appt, J = 5.5 Hz, 2H), 2.40−2.27 (m, 4H), 2.14 (s, 3H), 2.13−2.05 (m, 4H), 1.81 (d, J = 1.4 Hz, 3H). ^{13}C NMR (175 MHz, $CDCl_3$): d 173.8, 173.0, 163.8, 154.2, 144.3, 134.7, 123.1, 122.5, 116.6, 106.6, 70.1, 61.2, 55.9, 54.5 (2C), 36.0, 35.3, 35.0, 23.4 (2C), 22.8, 16.3, 11.7. LCMS (ESI) *m/z*: 417.2384 calcd for $C_{23}H_{33}N_2O_5^+$ [M + H]$^+$; Found 417.2391.

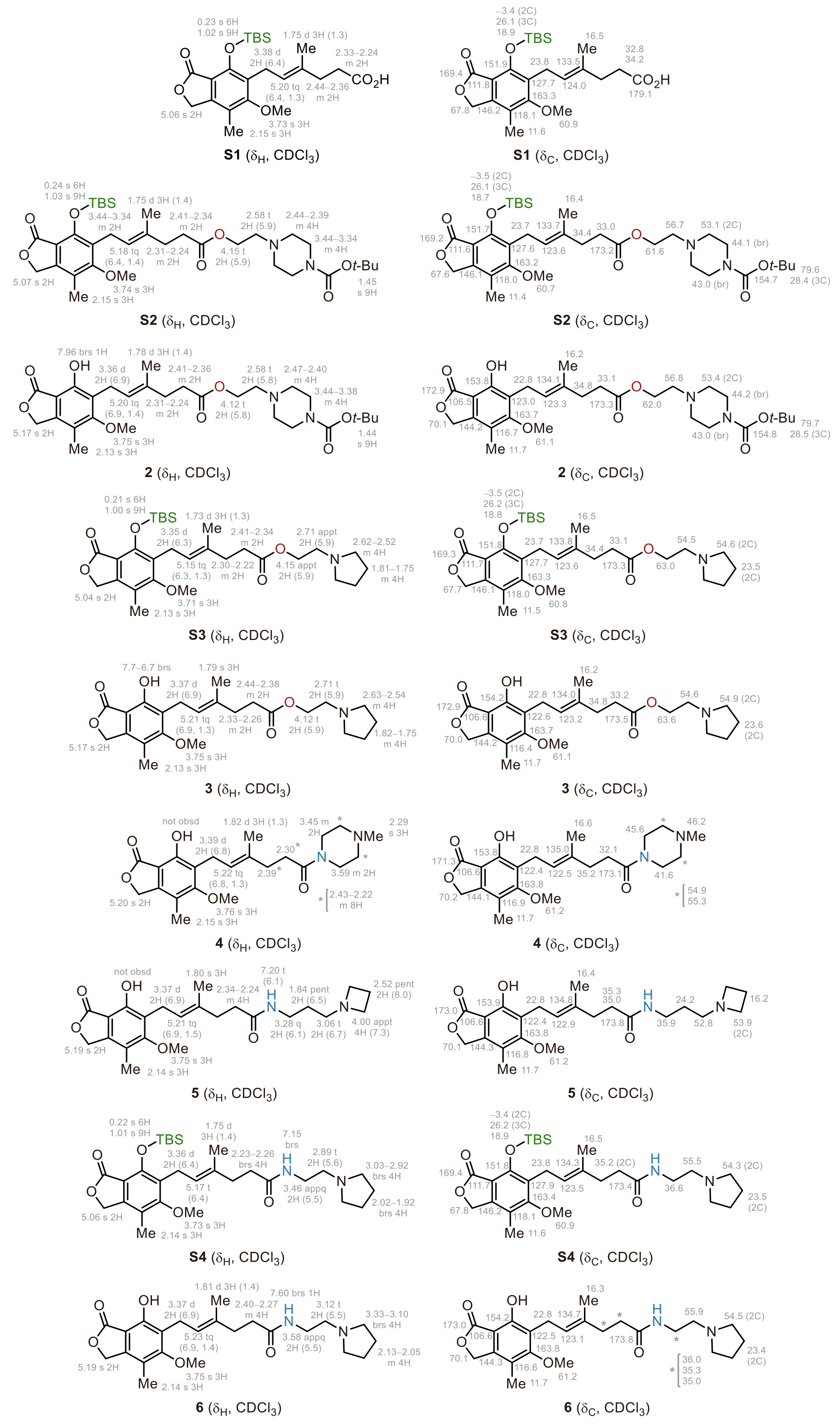

Figure S6. NMR assignments for mycophenolic esters and amides, related to Figure 4.

Data S1. ^{1}H and ^{13}C NMR Spectra, related to Figure 4.

(*E*)-6-(4-TBSO-6-methoxy-7-methyl-3-oxo-1,3-dihydroisobenzofuran-5-yl)-4-methylhex-4-enoic acid (**S1**) (^{1}H NMR; 400 MHz; $CDCl_3$)

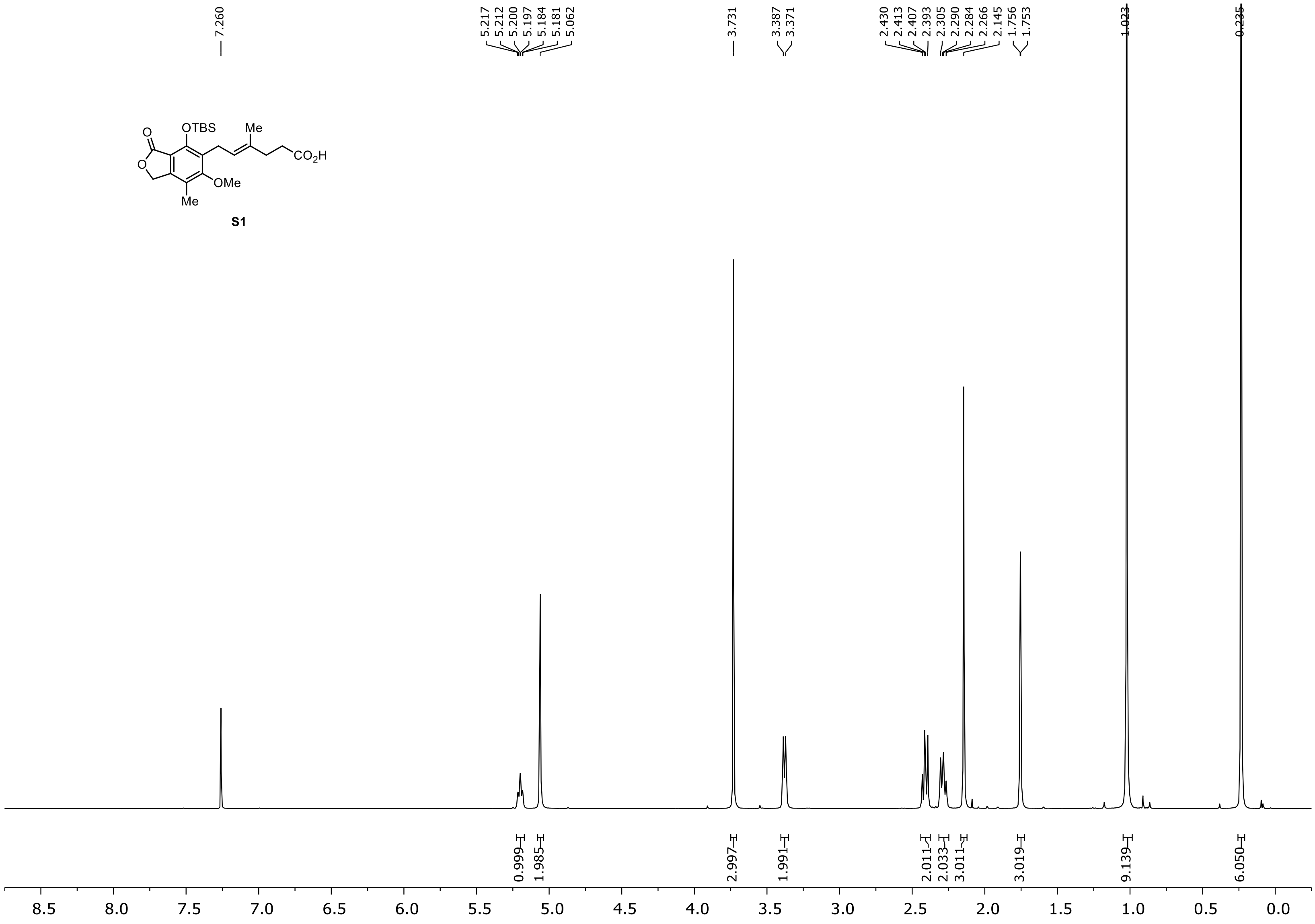

(*E*)-6-(4-*tert*-Butyldimethylsilyloxy-6-methoxy-7-methyl-3-oxo-1,3-dihydroisobenzofuran-5-yl)-4-methylhex-4-enoic acid (**S1**) (^{13}C NMR; 100 MHz; $CDCl_3$)

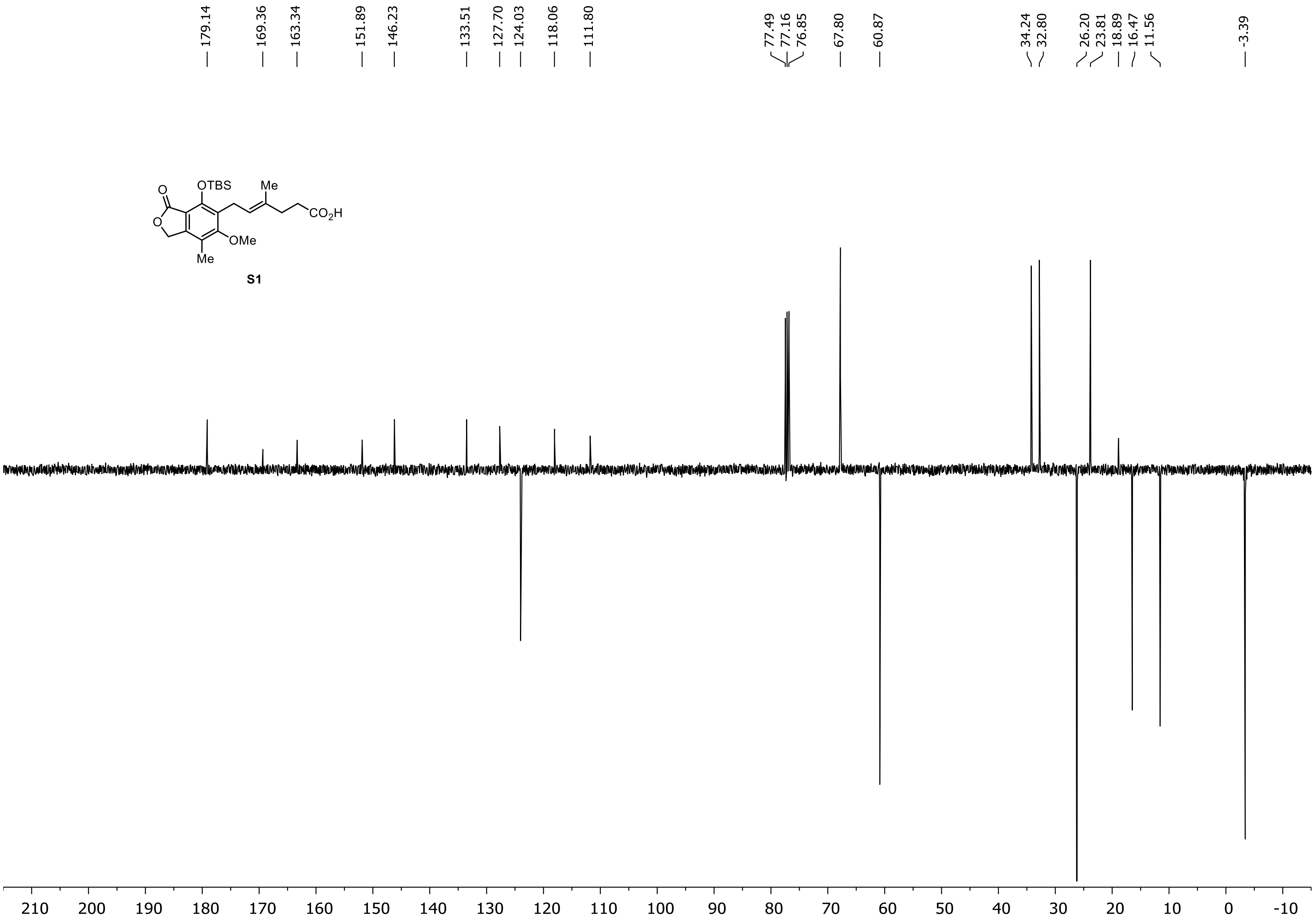

2-(4-Boc-piperazino)ethyl (*E*)-6-(4-*tert*-Butyldimethylsilyloxy-6-methoxy-7-methyl-3-oxo-1,3-dihydroisobenzofuran-5-yl)-4-methylhex-4-enoate (**S2**) (^{1}H NMR; 400 MHz; $CDCl_3$)

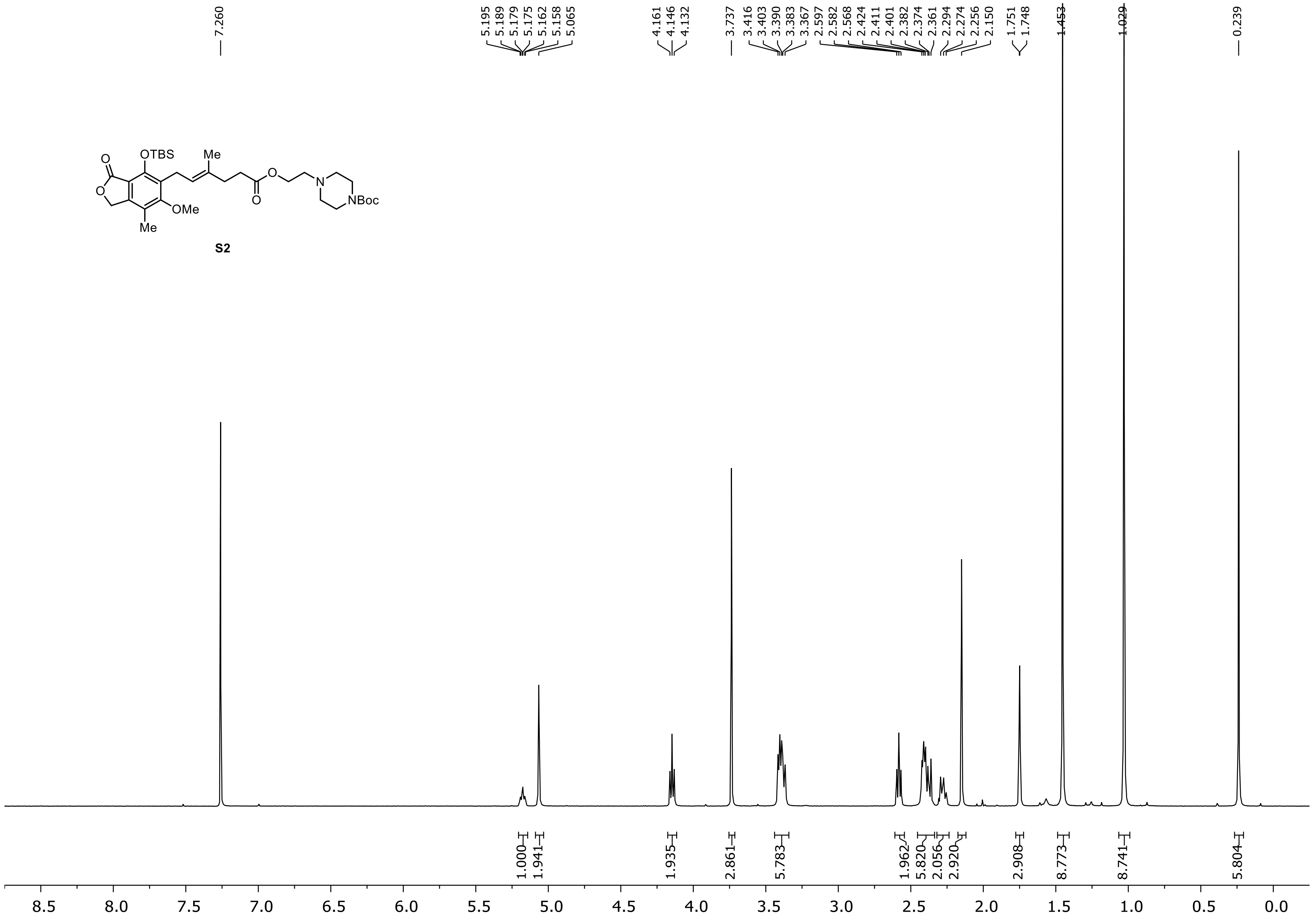

2-(4-Boc-piperazino)ethyl (*E*)-6-(4-*tert*-Butyldimethylsilyloxy-6-methoxy-7-methyl-3-oxo-1,3-dihydroisobenzofuran-5-yl)-4-methylhex-4-enoate (**S2**) (^{13}C NMR; 100 MHz; $CDCl_3$)

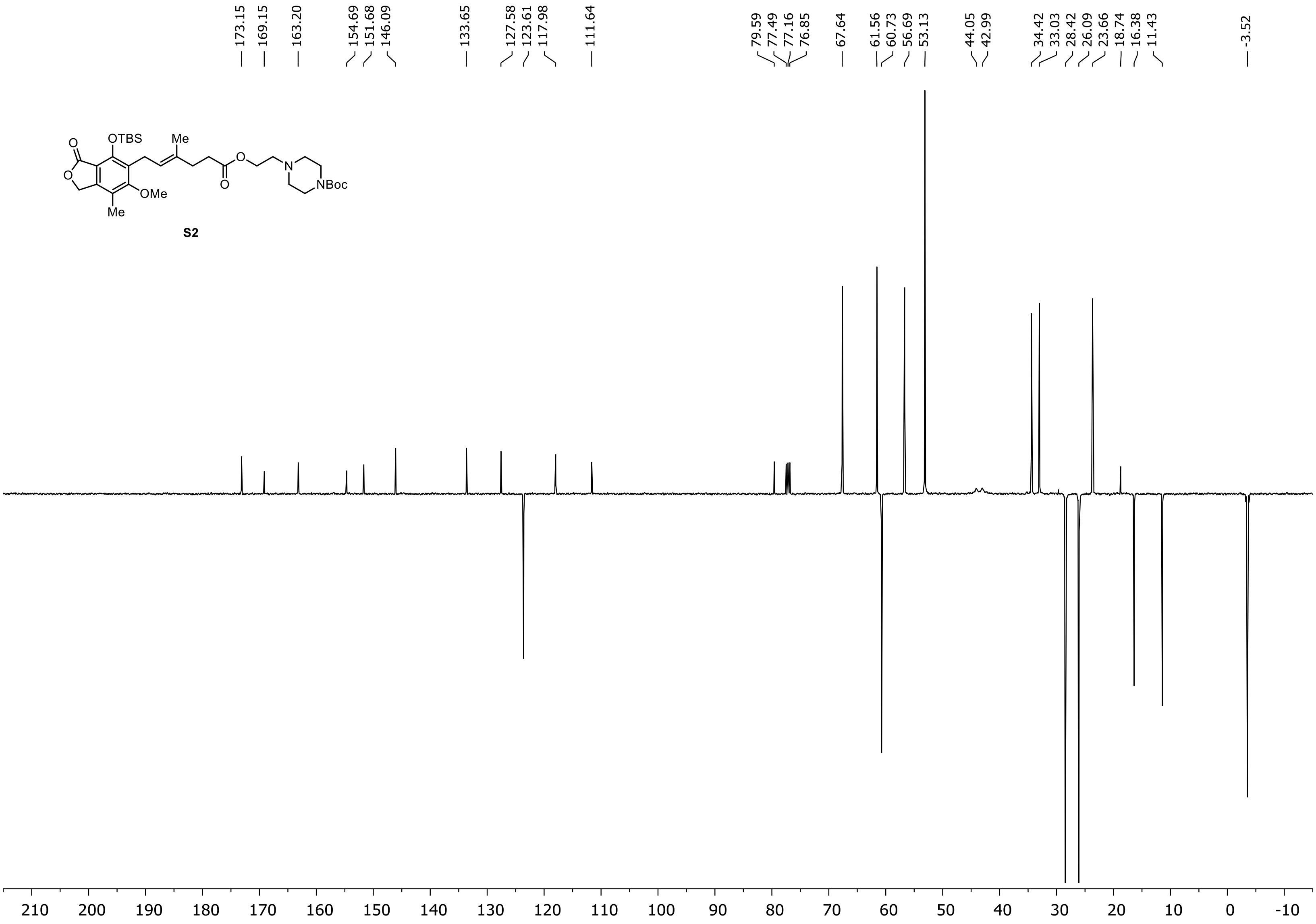

Mycophenolic acid, 2-(4-Boc-piperazino)ethyl ester (**2**) (^{1}H NMR; 400 MHz; $CDCl_3$)

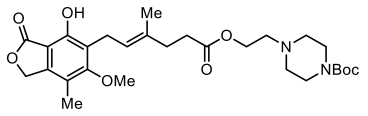

2

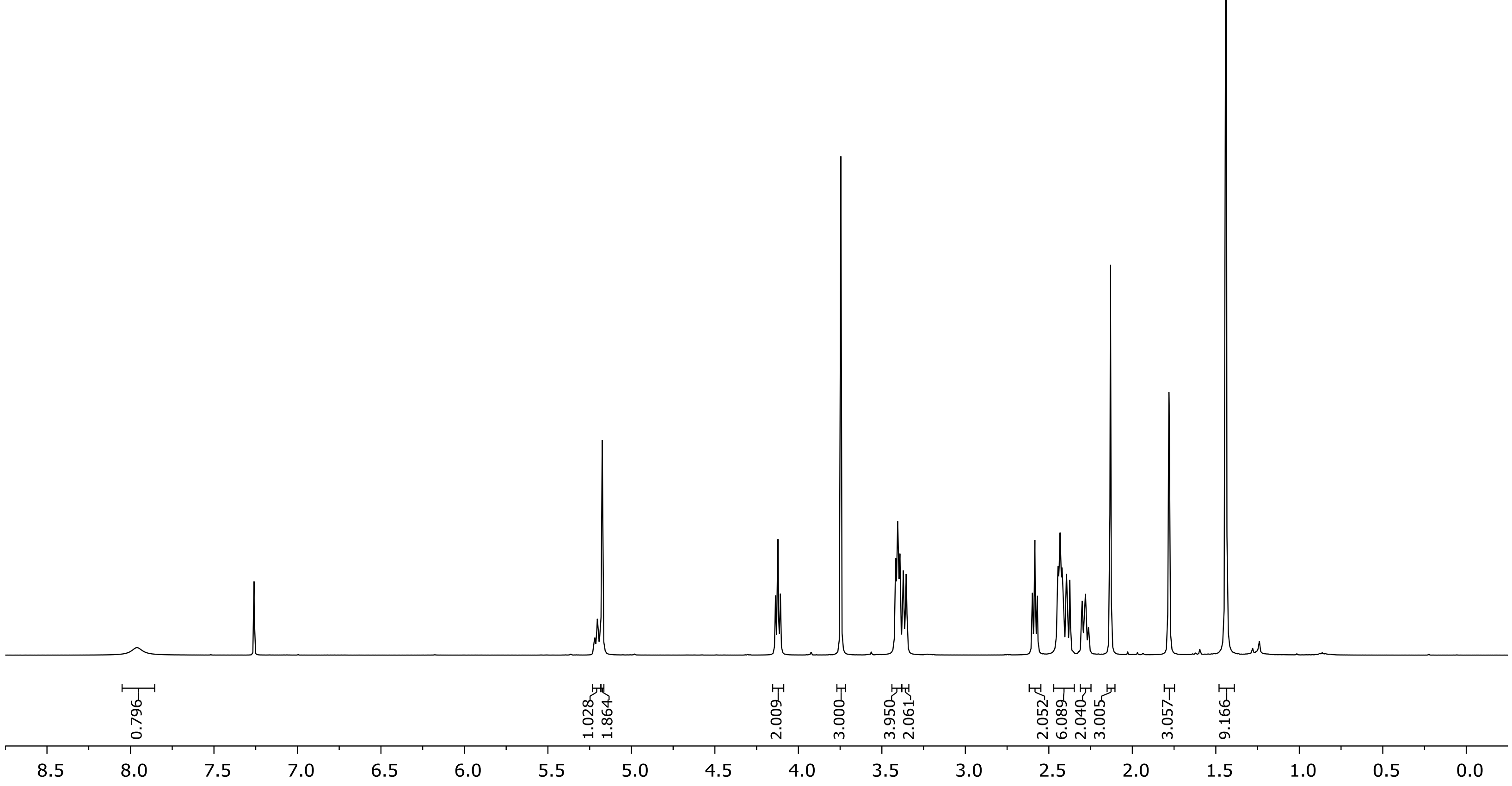

Mycophenolic acid, 2-(4-Boc-piperazino)ethyl ester (**2**) (^{13}C NMR; 100 MHz; $CDCl_3$)

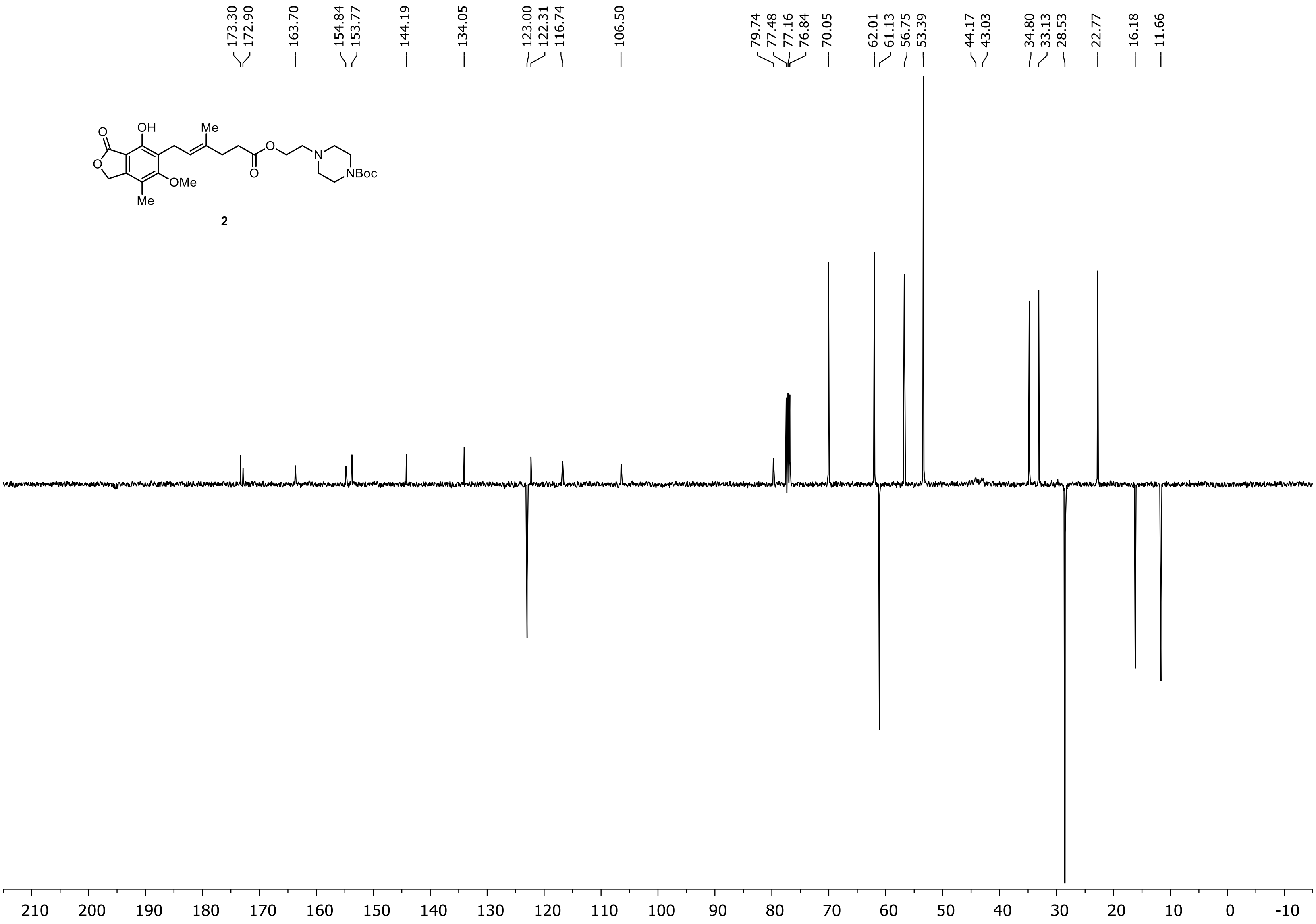

2-(1-Pyrrolidinyl)ethyl (*E*)-6-(4-*tert*-Butyldimethylsilyloxy-6-methoxy-7-methyl-3-oxo-1,3-dihydroisobenzofuran-5-yl)-4-methylhex-4-enoate (**S3**) (^{1}H NMR; 400 MHz; $CDCl_3$)

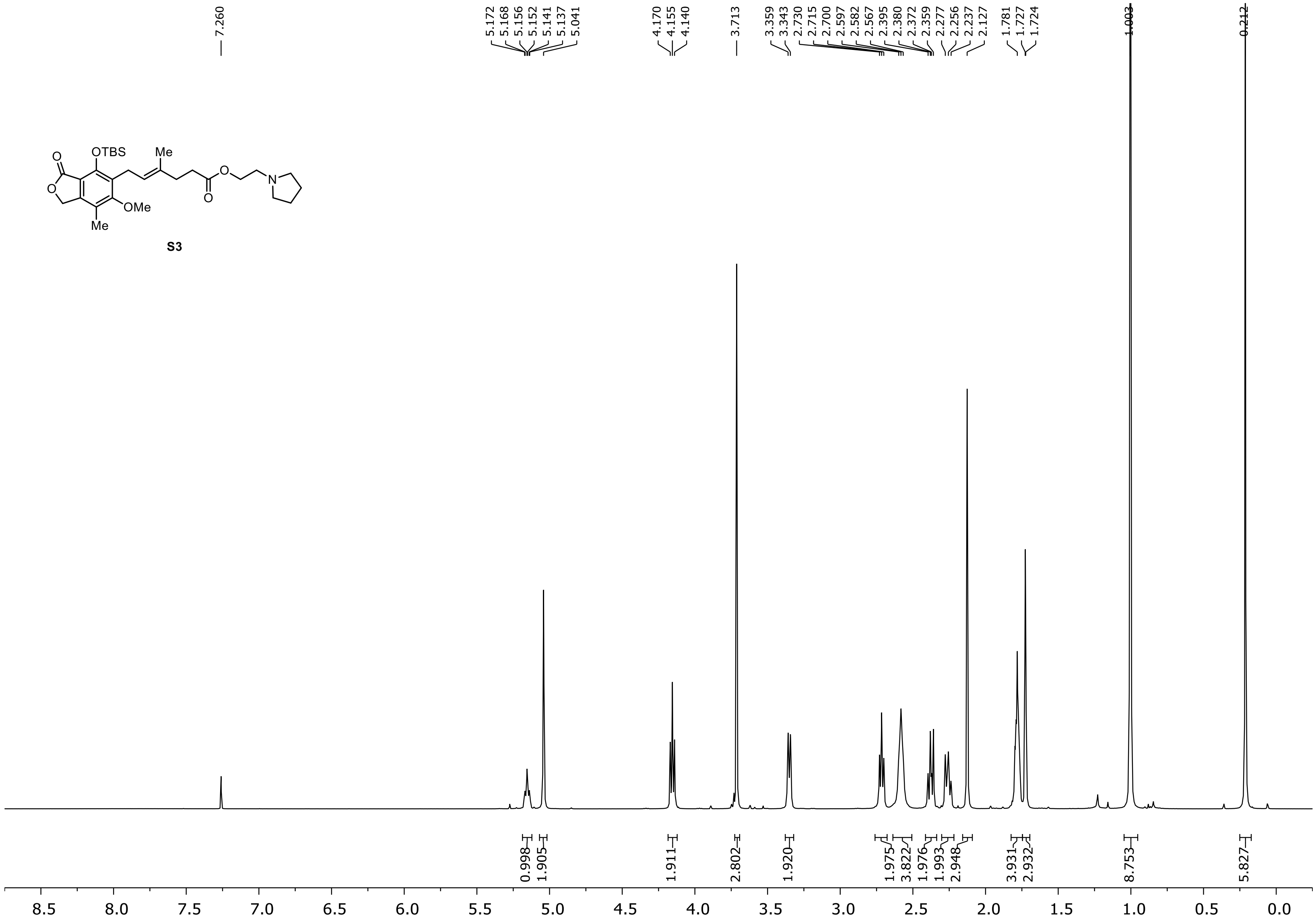

2-(1-Pyrrolidinyl)ethyl (*E*)-6-(4-*tert*-Butyldimethylsilyloxy-6-methoxy-7-methyl-3-oxo-1,3-dihydroisobenzofuran-5-yl)-4-methylhex-4-enoate (**S3**) (^{13}C NMR; 100 MHz; $CDCl_3$)

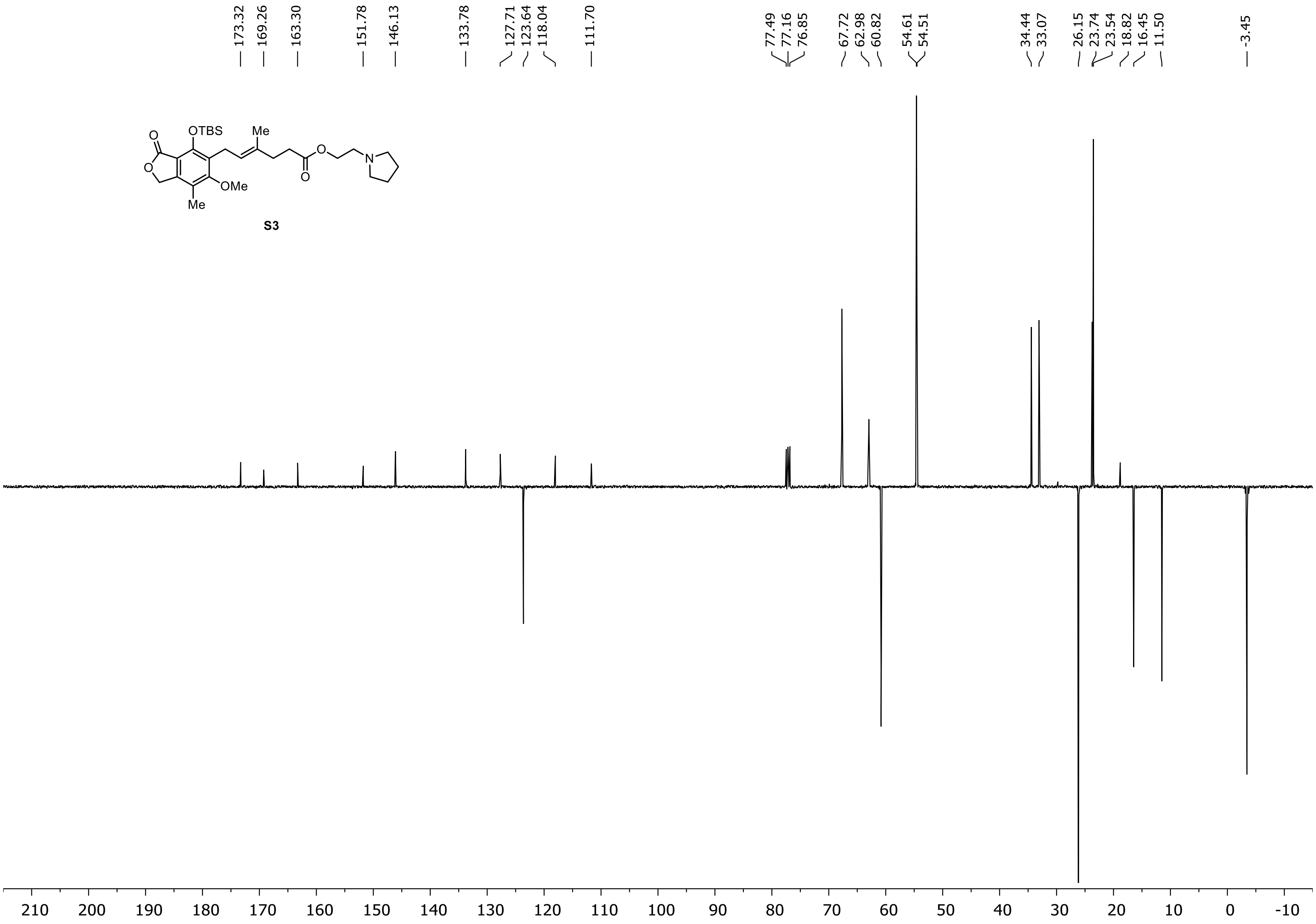

Mycophenolic acid, 2-(1-pyrrolidinyl)ethyl ester (**3**) (^{1}H NMR; 400 MHz; $CDCl_3$)

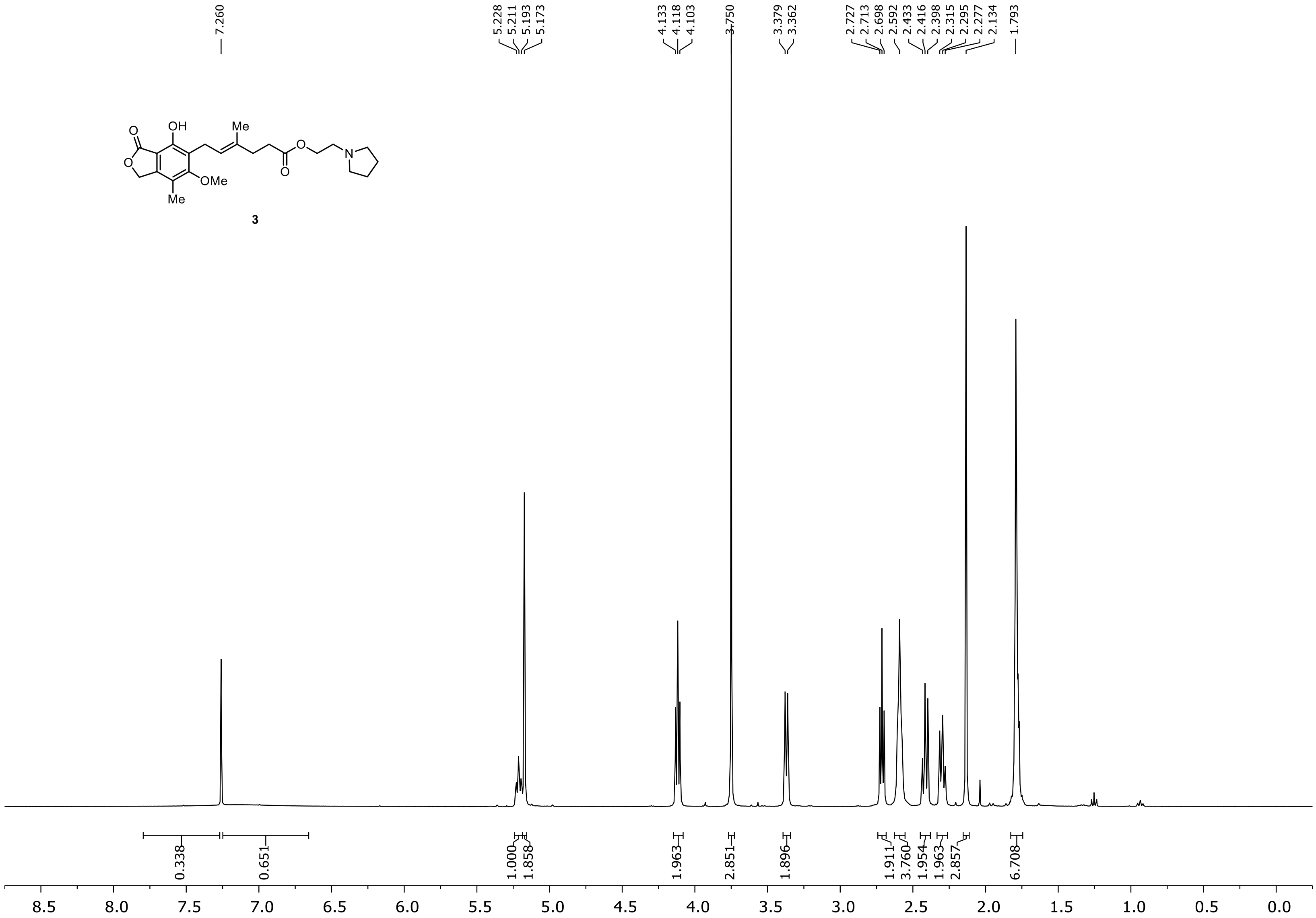

Mycophenolic acid, 2-(1-pyrrolidinyl)ethyl ester (**3**) (^{13}C NMR; 100 MHz; $CDCl_3$)

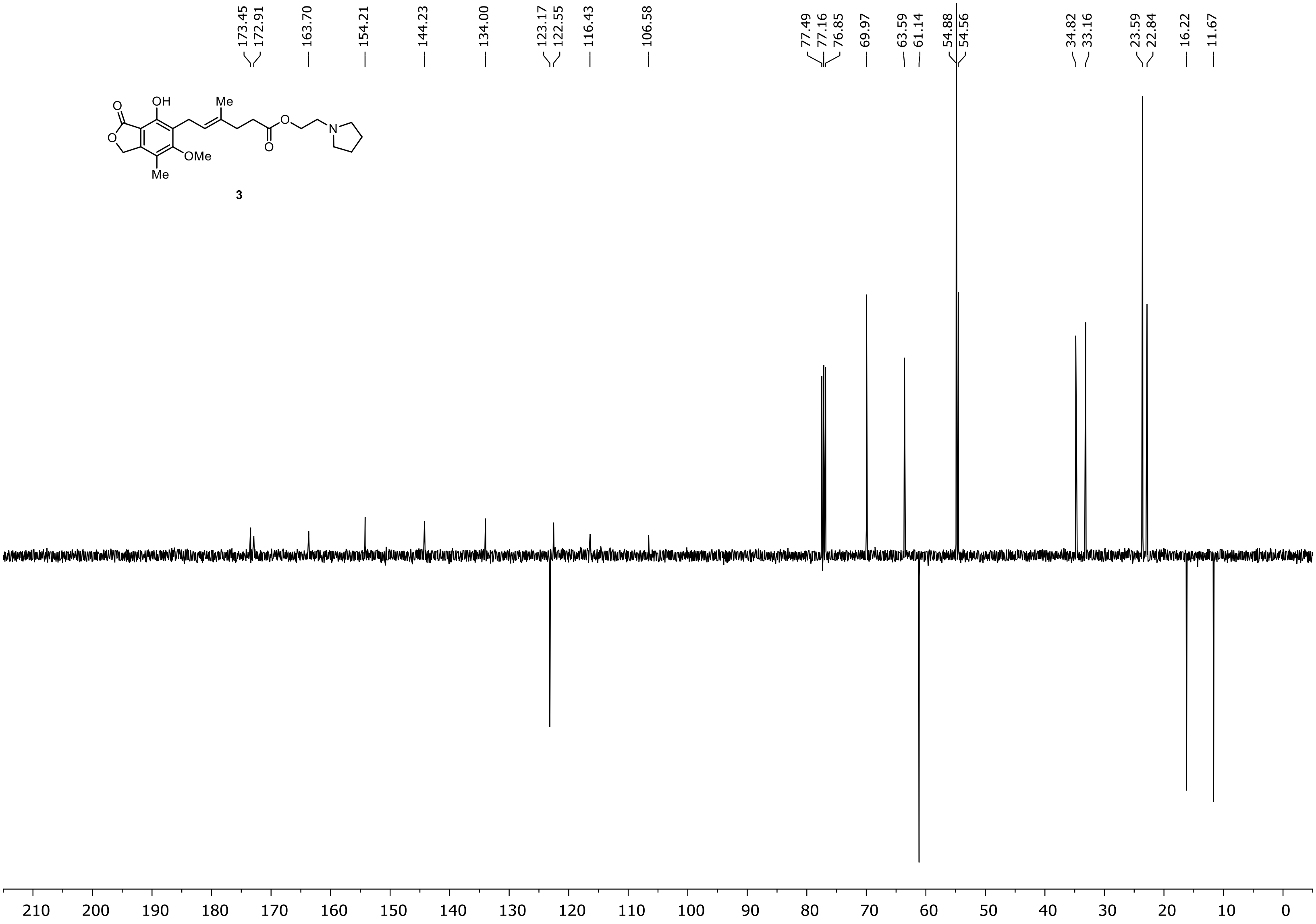

Mycophenolic acid, 4-methylpiperazine amide (**4**) (^{1}H NMR; 400 MHz; $CDCl_3$)

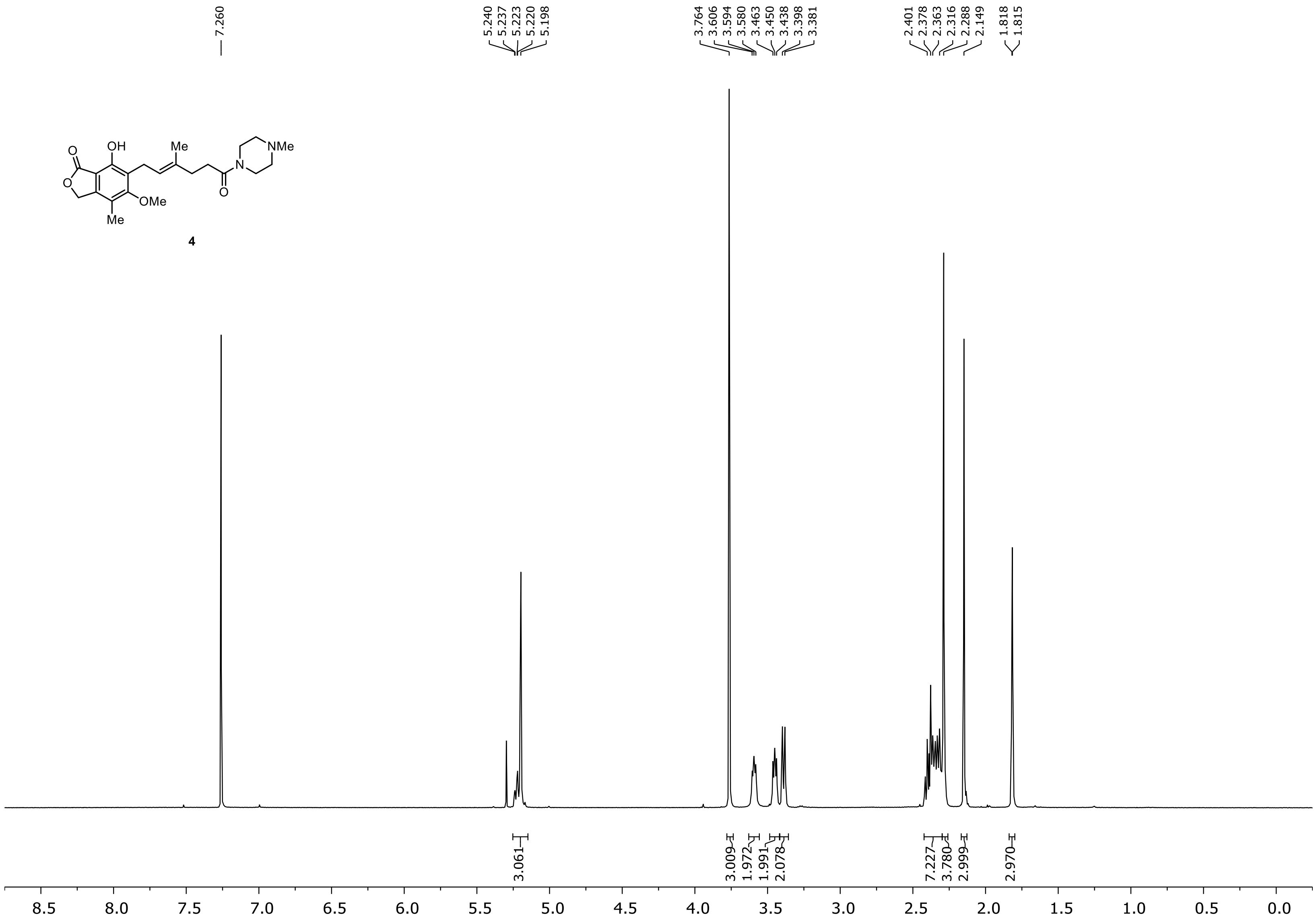

Mycophenolic acid, 4-methylpiperazine amide (**4**) (^{13}C NMR; 100 MHz; $CDCl_3$)

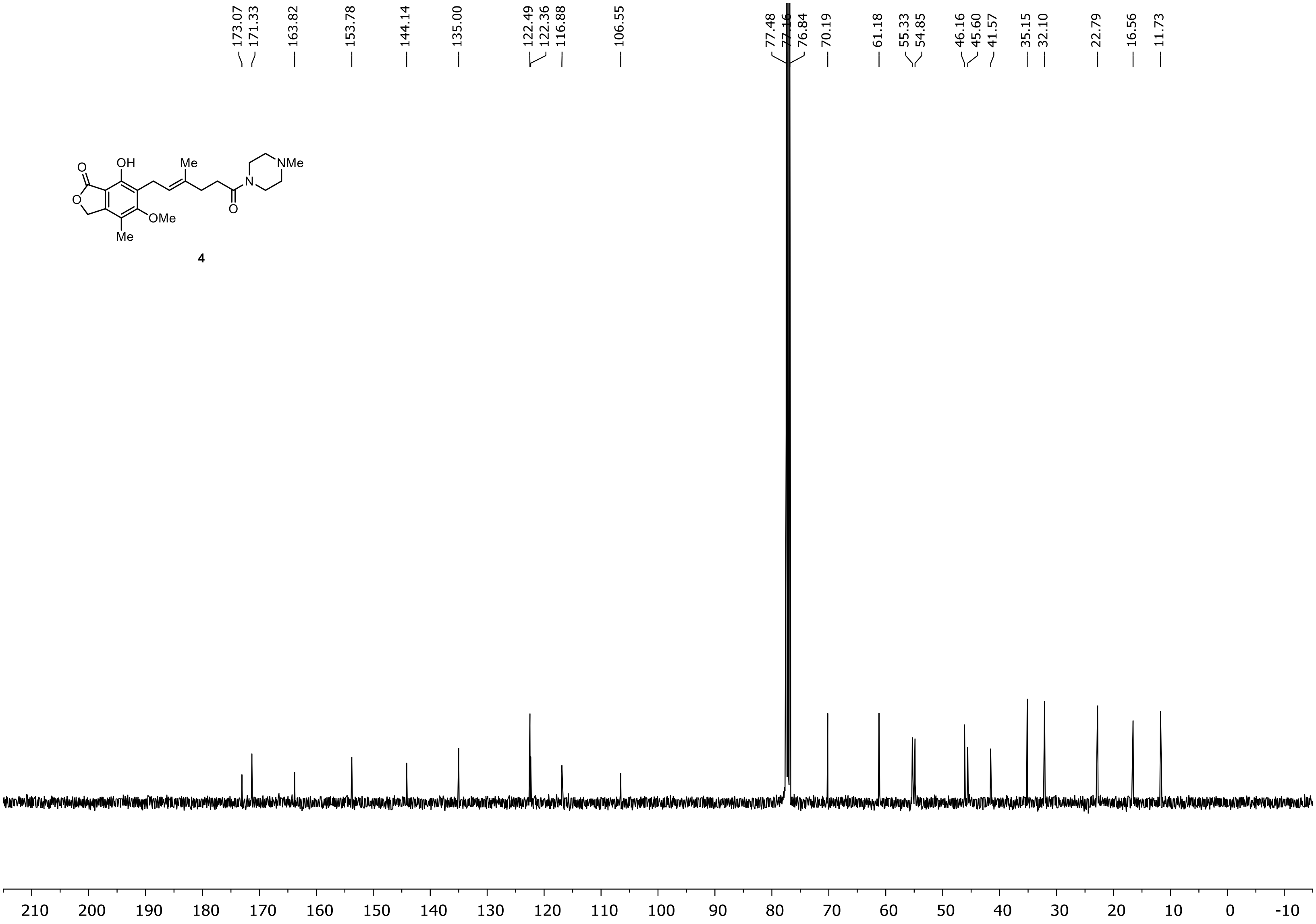

Mycophenolic acid, 4-methylpiperazine amide (**4**) (HSQC; $CDCl_3$)

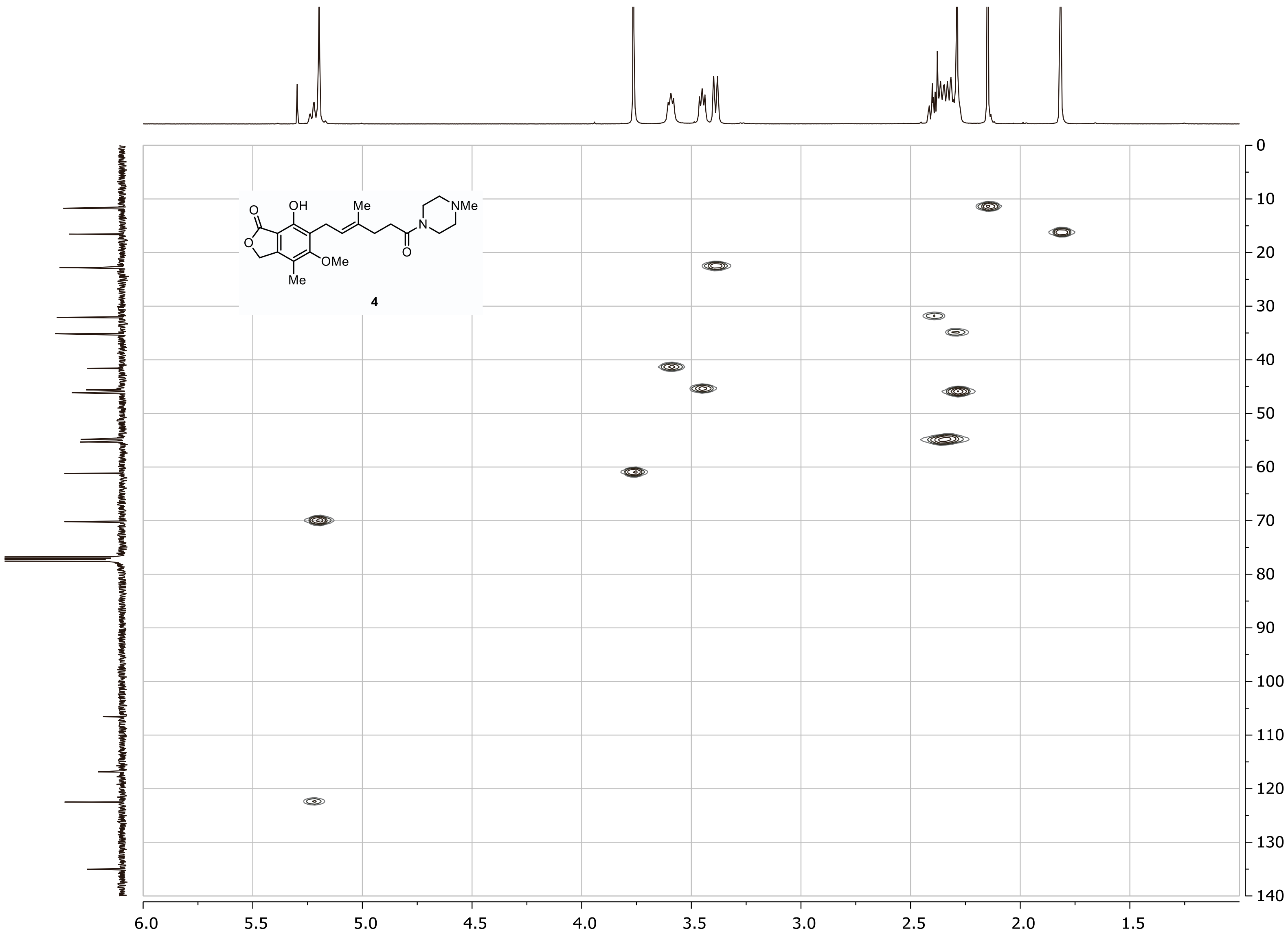

Mycophenolic acid, 4-methylpiperazine amide (**4**) (HMBC; $CDCl_3$)

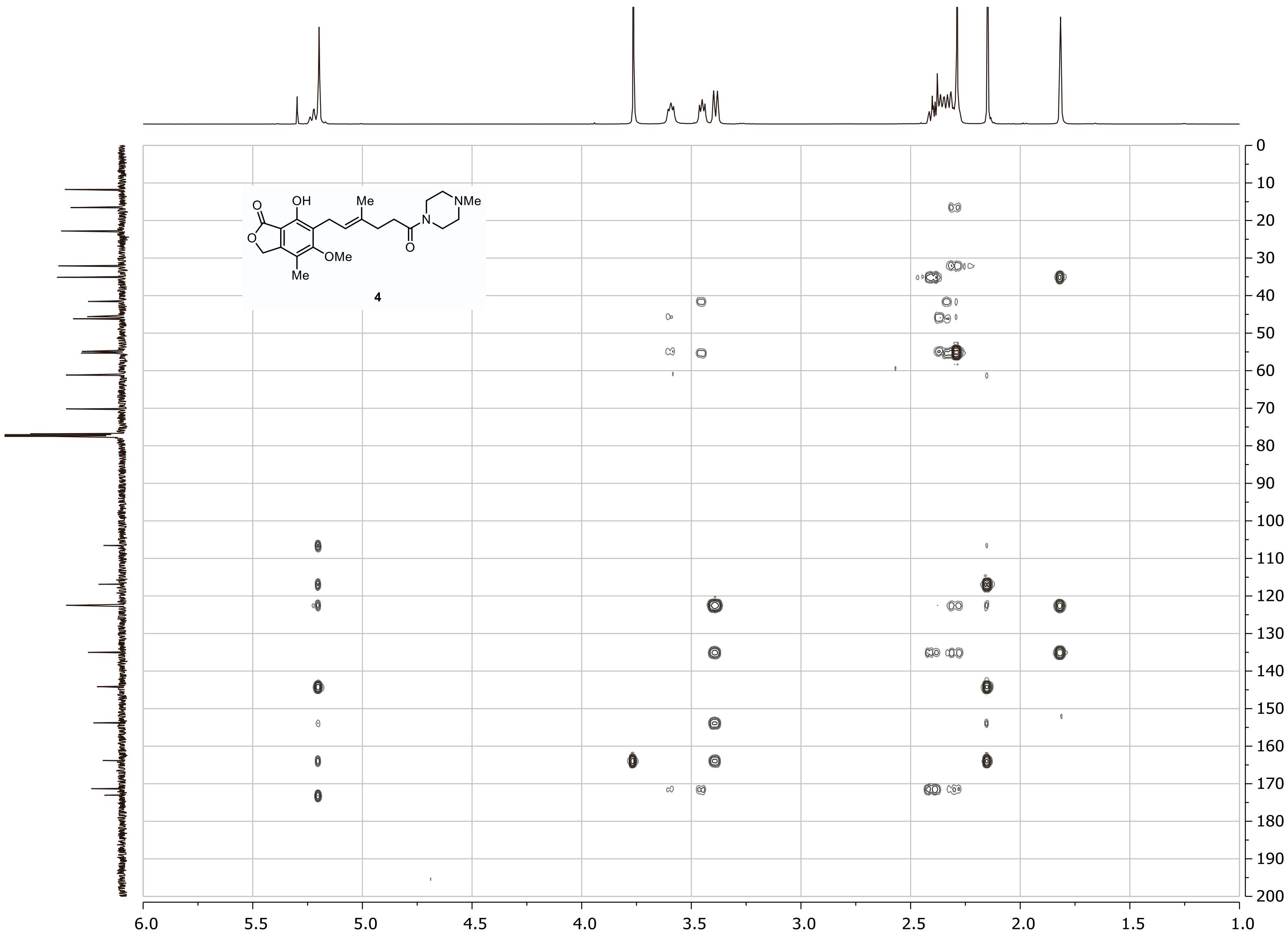

Mycophenolic acid, 3-(azetidin-1-yl)propyl amide (**5**) (^{1}H NMR; 400 MHz; $CDCl_3$)

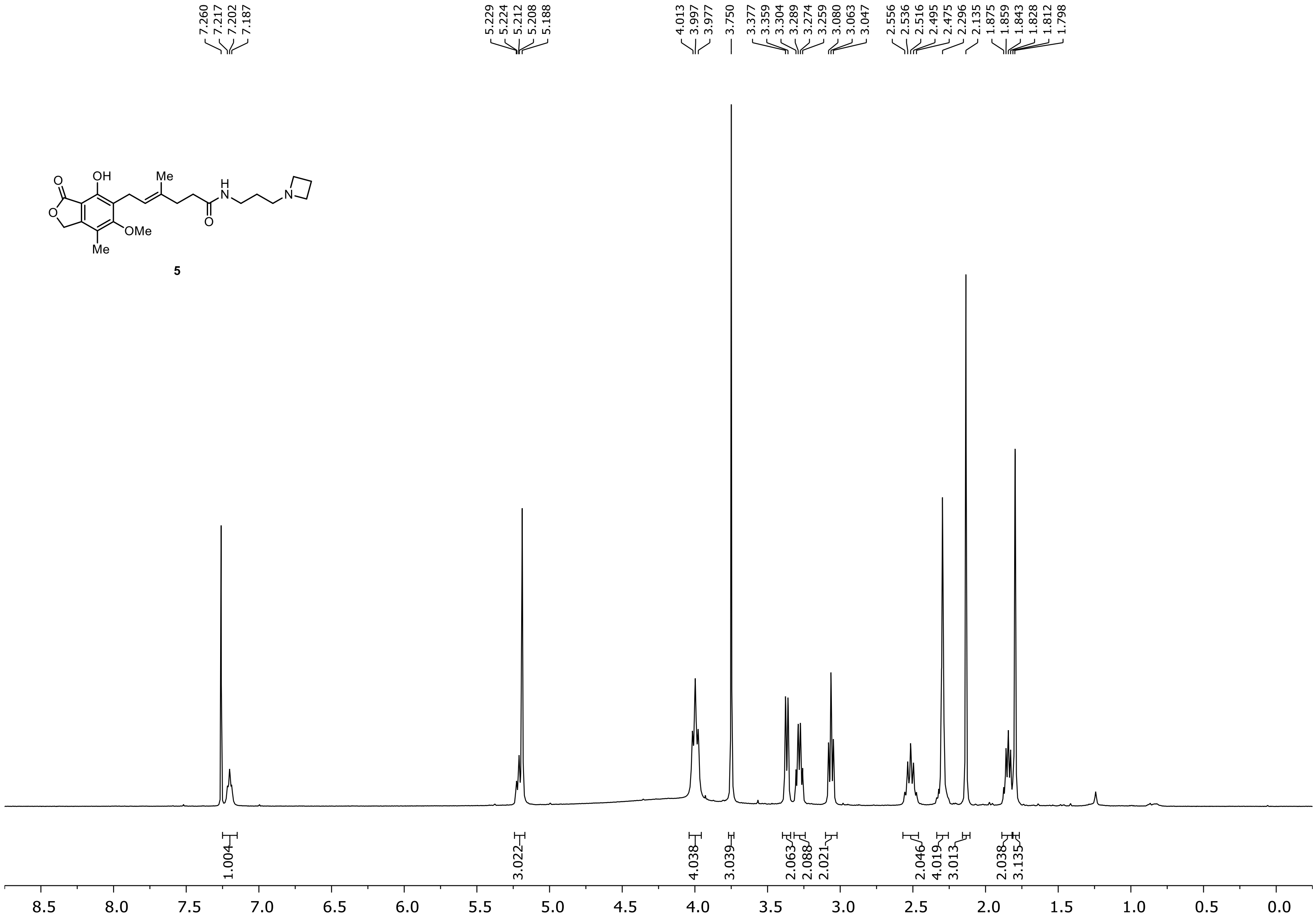

Mycophenolic acid, 3-(azetidin-1-yl)propyl amide (**5**) (^{13}C NMR; 100 MHz; $CDCl_3$)

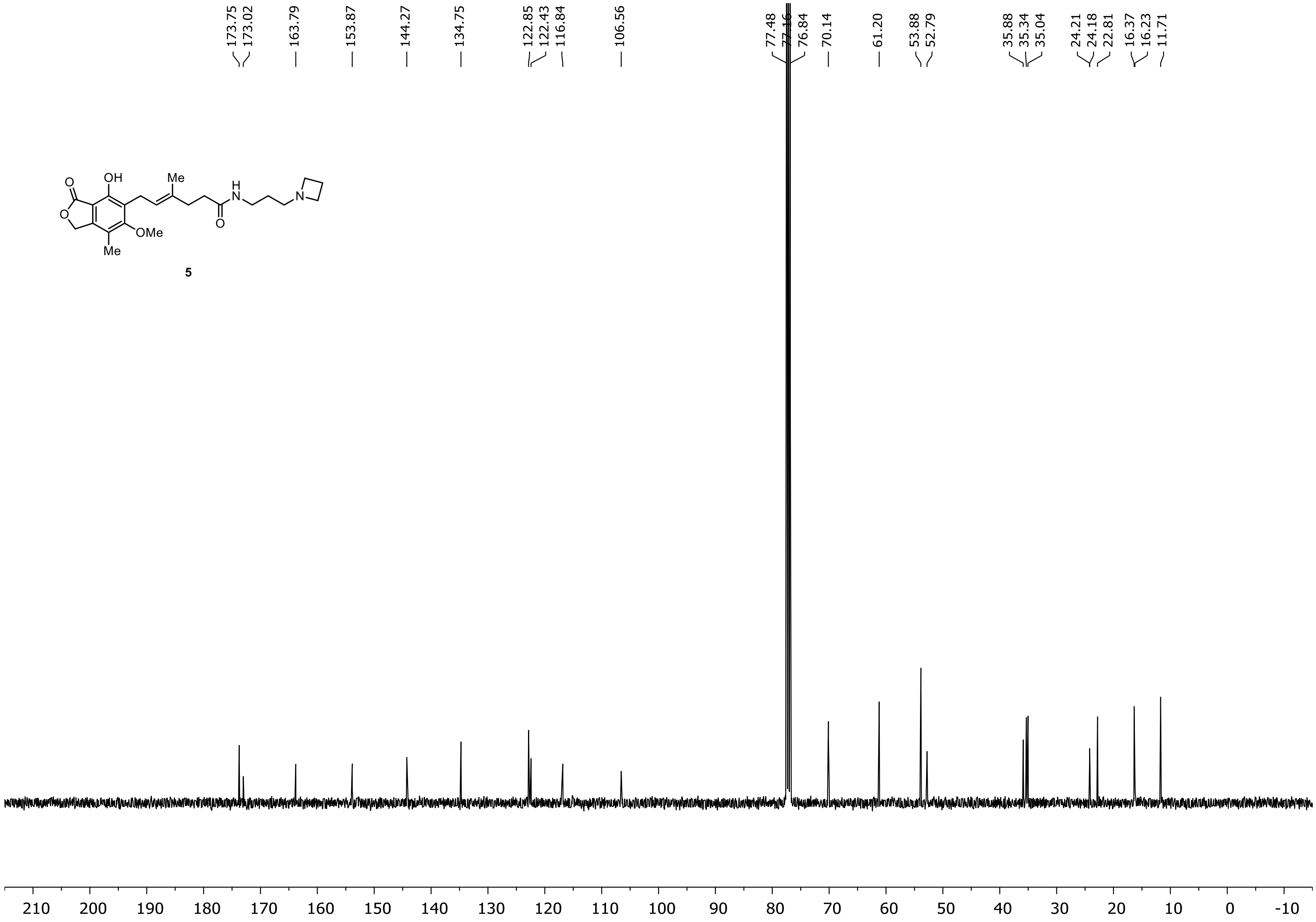

Mycophenolic acid, 3-(azetidin-1-yl)propyl amide (**5**) (HSQC; $CDCl_3$)

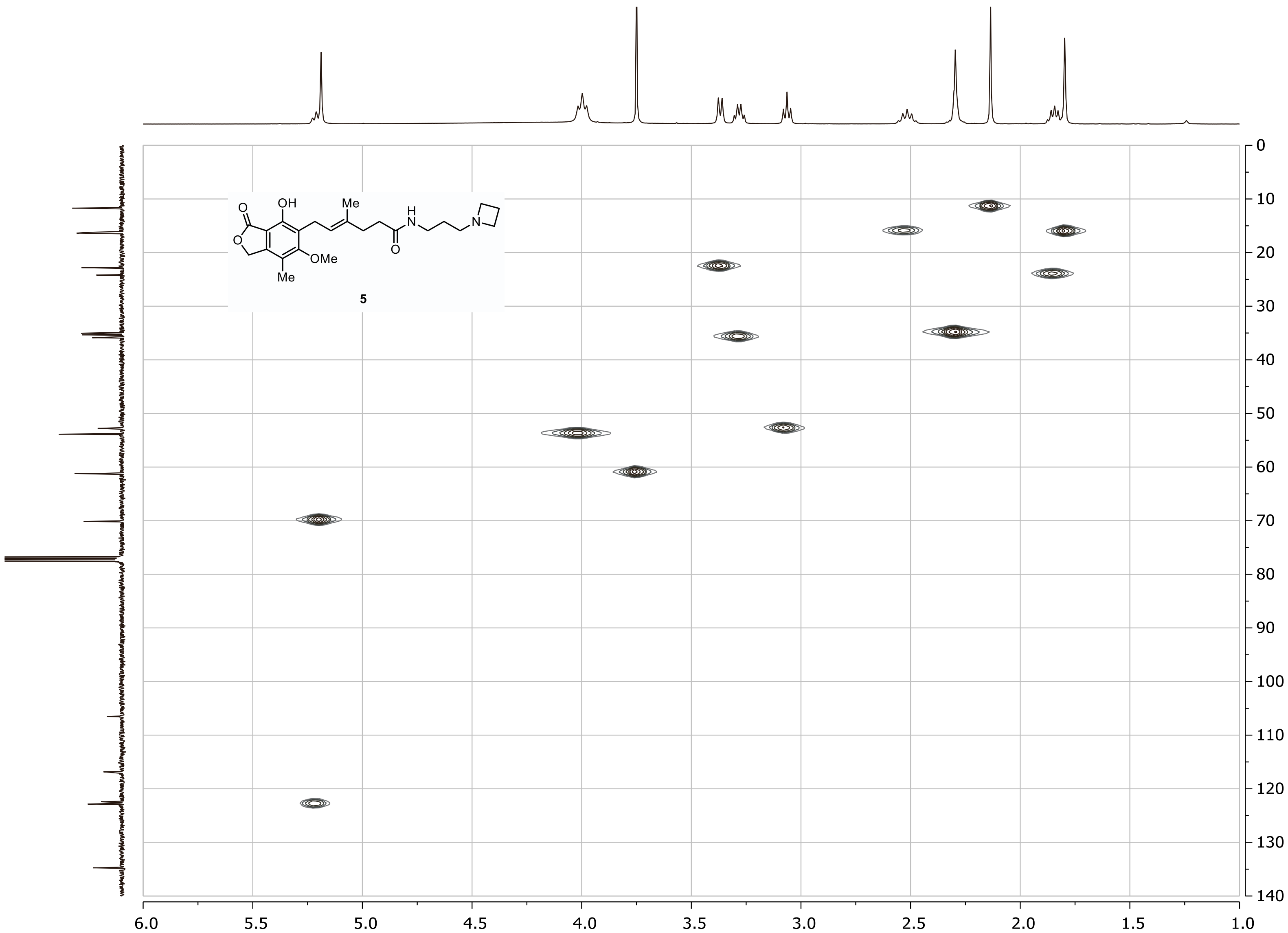

Mycophenolic acid, 3-(azetidin-1-yl)propyl amide (**5**) (HMBC; $CDCl_3$)

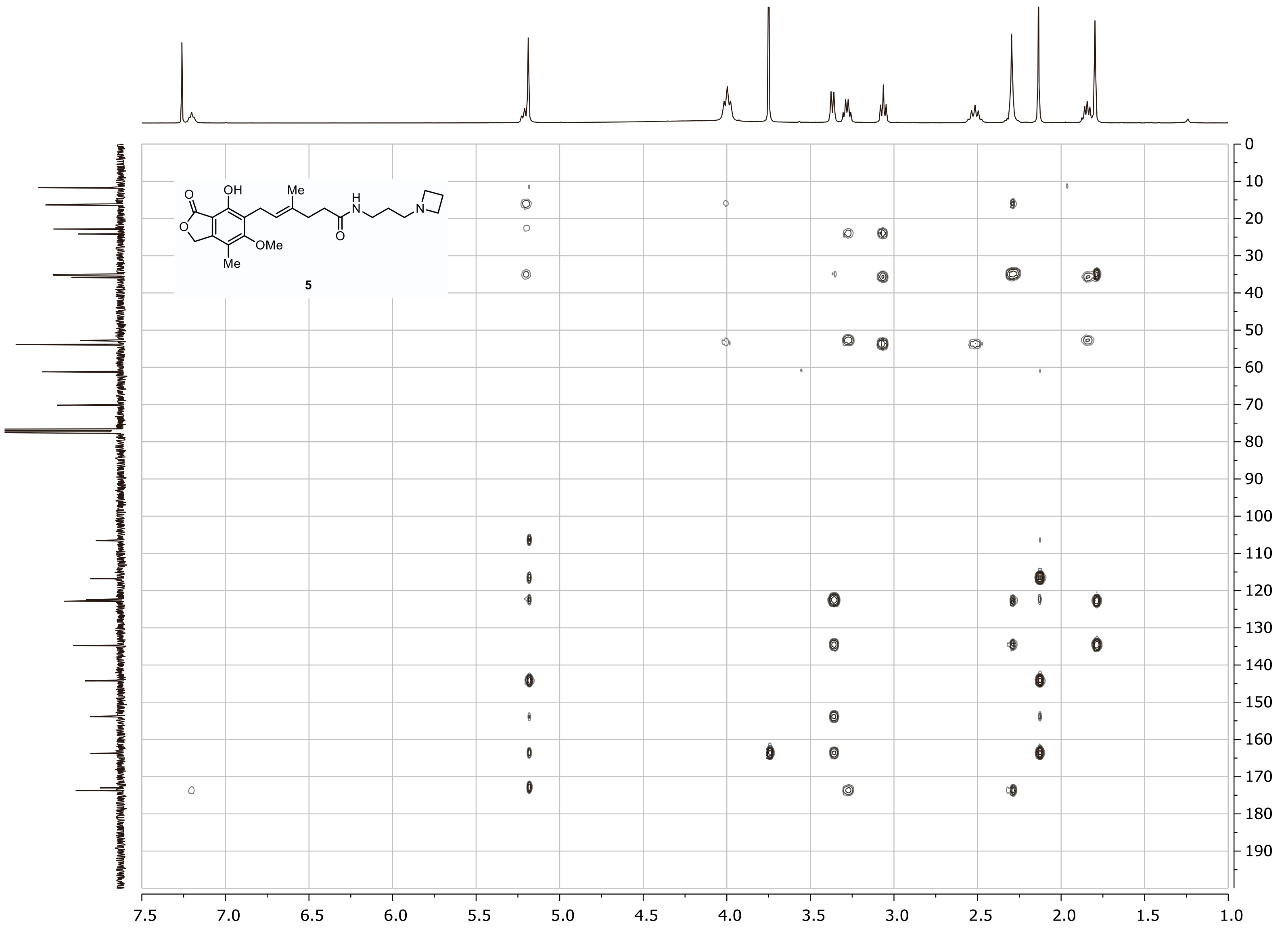

2-(1-Pyrrolidinyl)ethyl (*E*)-6-(4-*tert*-butyldimethylsilyloxy-6-methoxy-7-methyl-3-oxo-1,3-dihydroisobenzofuran-5-yl)-4-methylhex-4-enoate (**S4**) (^{1}H NMR; 400 MHz; $CDCl_3$)

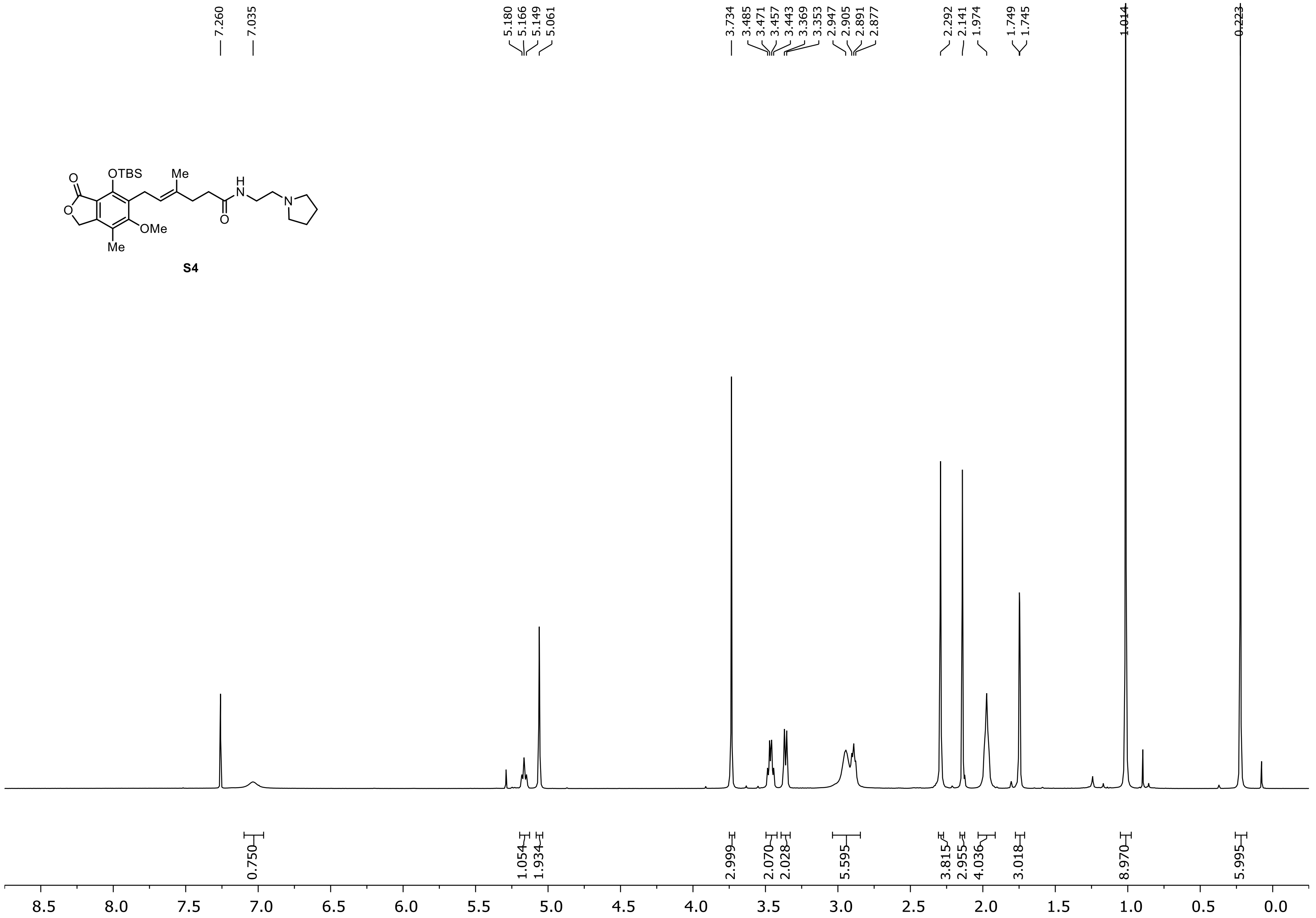

2-(1-Pyrrolidinyl)ethyl (*E*)-6-(4-*tert*-butyldimethylsilyloxy-6-methoxy-7-methyl-3-oxo-1,3-dihydroisobenzofuran-5-yl)-4-methylhex-4-enoate (**S4**) (^{13}C NMR; 100 MHz; $CDCl_3$)

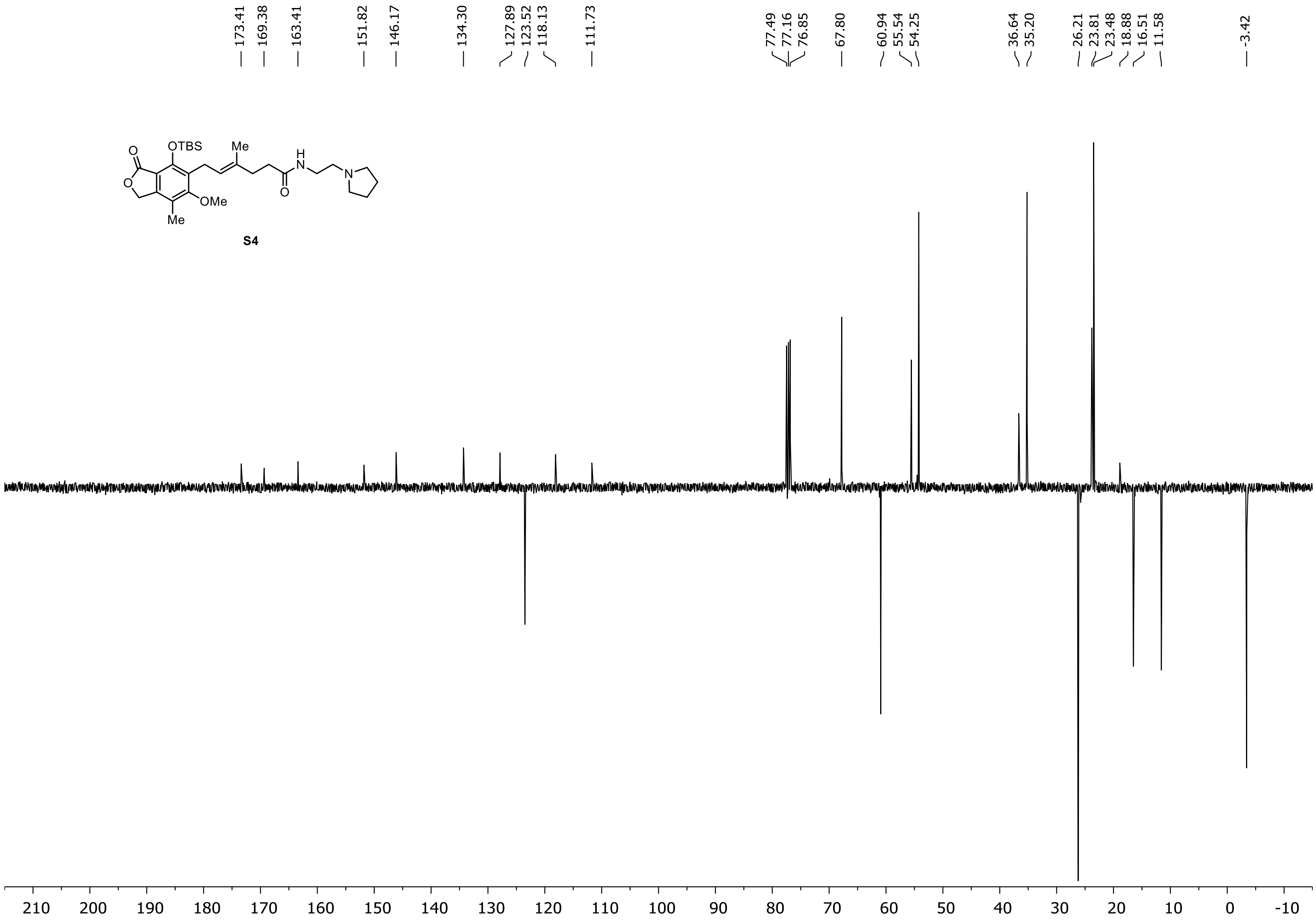

Mycophenolic acid, 2-(1-pyrrolidinyl)ethyl amide (**6**) (^{1}H NMR; 400 MHz; $CDCl_3$)

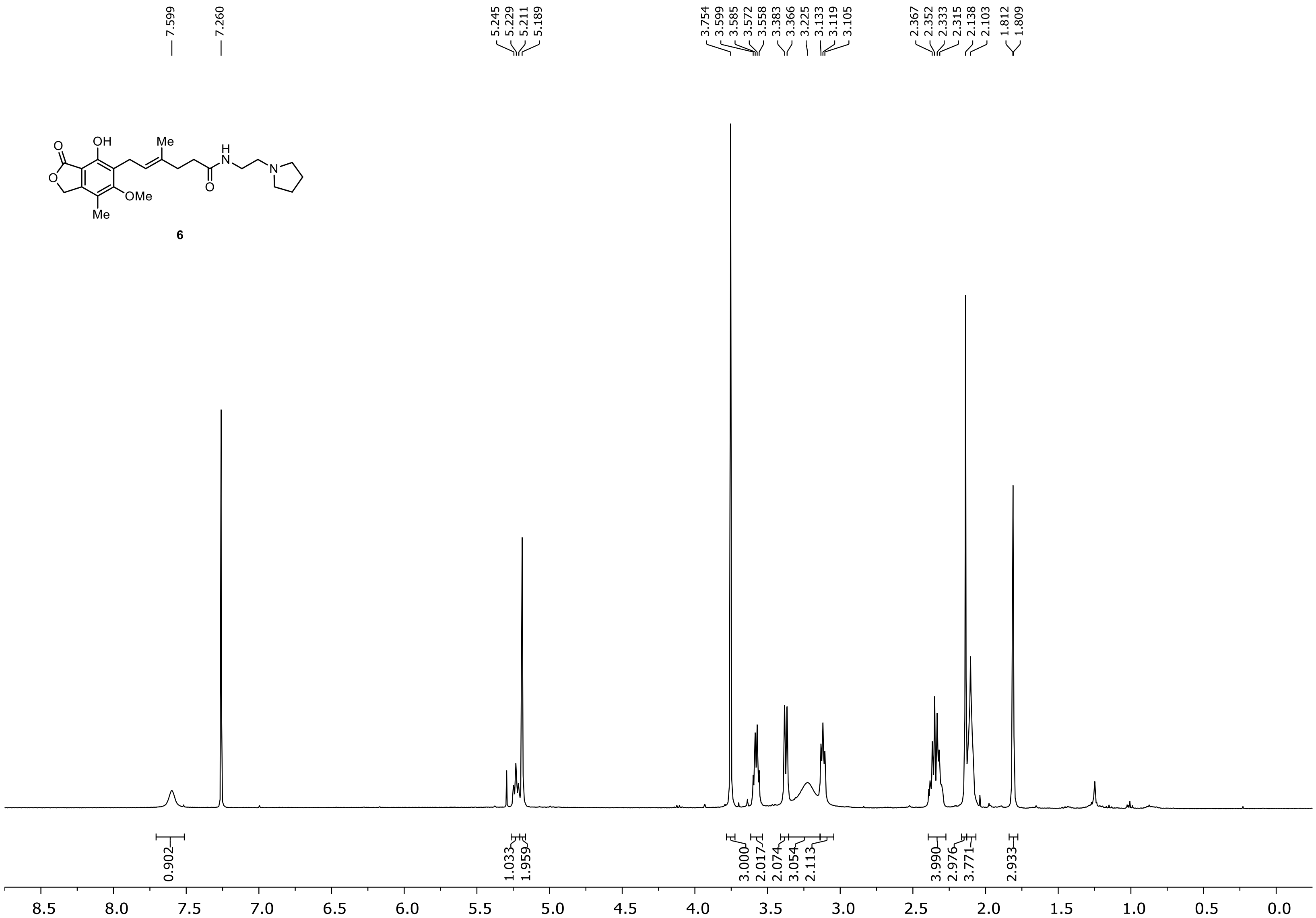

6

Mycophenolic acid, 2-(1-pyrrolidinyl)ethyl amide (**6**) (^{13}C NMR; 100 MHz; $CDCl_3$)

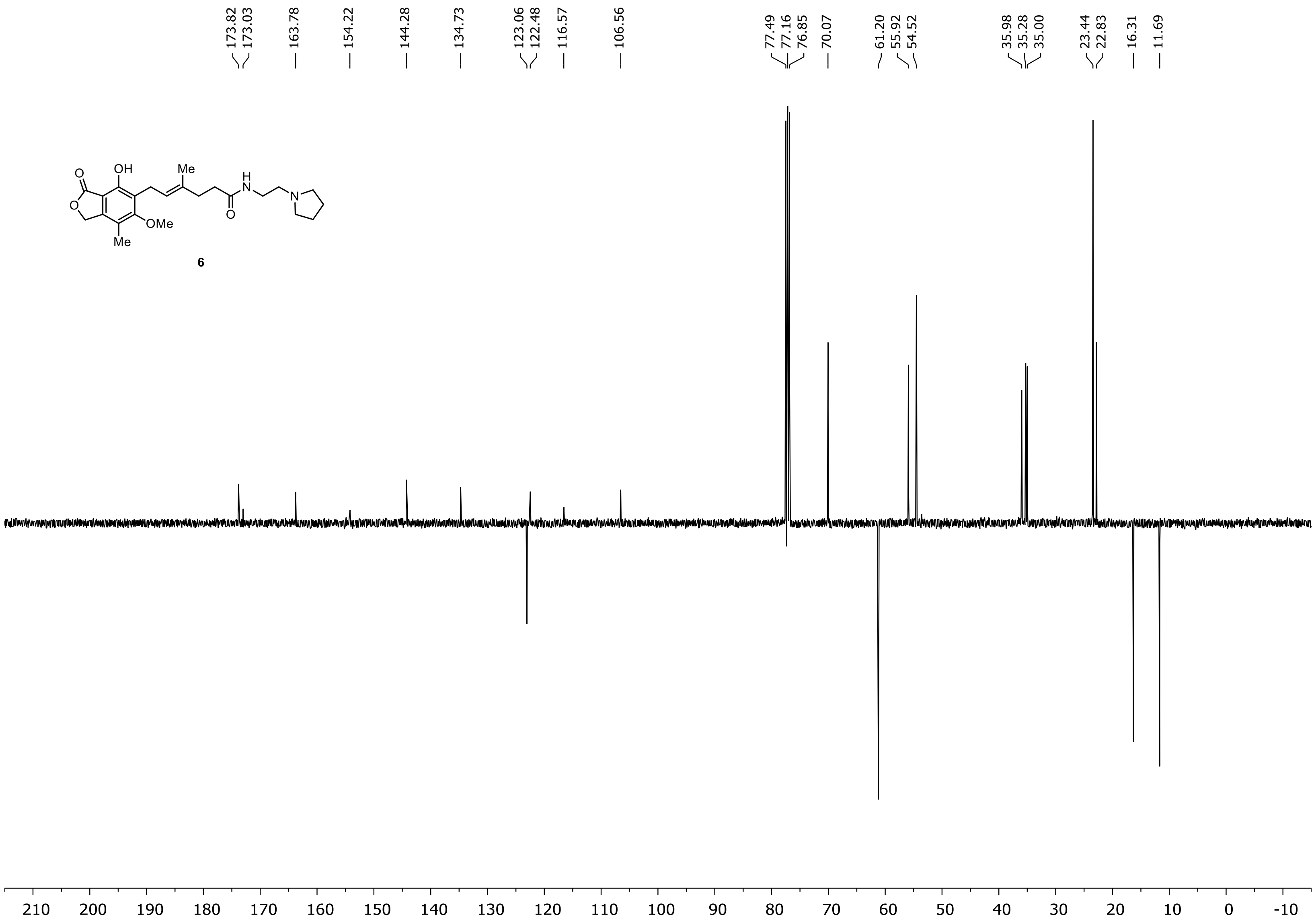

Supplemental References

S1. Cholewiński, G., Iwaszkiewicz-Grześ, D., Prejs, M., Głowacka, A., and Dzierzbicka, K. (2015). Synthesis of the inosine 5′-monophosphate dehydrogenase (IMPDH) inhibitors. J. Enzyme Inhib. Med. Chem. *30*, 550–563. 10.3109/14756366.2014.951349.
